# Longitudinal Pointwise Visual Field Prediction in Early‐Stage Glaucoma Using Regression Models and Long Short‐Term Memory: A Simulation Study

**DOI:** 10.1155/bmri/2815308

**Published:** 2026-10-21

**Authors:** Soheila Naderi, Eghbal Mansoori, Masoumeh Sadat Masoumpour, Marziyeh Doostfatemeh, Saeedeh Pourahmad

**Affiliations:** ^1^ Department of Biostatistics School of Medicine Shiraz University of Medical Sciences Shiraz Iran sums.ac.ir; ^2^ School of Electrical and Computer Engineering Shiraz University Shiraz Iran shirazu.ac.ir; ^3^ Poostchi Ophthalmology Research Center Department of Ophthalmology School of Medicine Shiraz University of Medical Sciences Shiraz Iran sums.ac.ir

**Keywords:** autoregressive model, glaucoma, LSTM, OLR, visual field prediction

## Abstract

**Background:**

Glaucoma is a progressive optic neuropathy in which longitudinal visual field (VF) prediction is essential for timely clinical decision‐making and preventing irreversible VF loss. However, accurate prediction remains challenging due to the noisy, high‐dimensional nature of VF data and their complex longitudinal patterns. Capturing these patterns may require increasingly sophisticated prediction models, but greater model complexity can reduce interpretability. Therefore, prediction models need to capture temporal patterns while balancing interpretability and computational feasibility. This study is aimed at evaluating and comparing the performance of four methods, including personalized models (first‐order autoregressive [AR(1)] and ordinary linear regression (OLR)), a population‐level long short‐term memory (LSTM), and a naive baseline (BL) forecast for glaucoma VF prediction.

**Materials and Methods:**

Data were simulated for patients with early‐stage glaucoma using a linear mixed effects model (LMM) to account for noise in VF measurements and the impact of individual effects on glaucoma progression. Future VFs were predicted from examinations 6–11 based on previous examinations. Prediction accuracy was assessed using root mean squared error (RMSE) and pointwise mean absolute error (PMAE).

**Results:**

Evaluation of the four approaches showed that OLR and naive BL forecast achieved higher errors than AR(1) and LSTM models. LSTM performed best when the number of examinations was small (in the initial examinations, indeed) in slow, moderate, and fast progression rate scenarios. However, with a larger number of examinations, prediction errors increased for LSTM compared with AR(1), resulting in higher accuracy for AR(1), particularly under moderate and fast progression rate scenarios.

**Conclusion:**

The performance of the models shows dependence on the number of examinations in the present study. These findings highlight the importance of temporal information in longitudinal VF prediction.

## 1. Introduction

Glaucoma is recognized as a leading cause of irreversible blindness worldwide [1–3]. The disease is characterized by retinal ganglion cell (RGC) death and functional visual field (VF) defects [4]. Standard automated perimetry (SAP) is employed as a primary functional test for the detection and monitoring of glaucoma [5, 6]. Early and reliable detection of VF progression in glaucoma is critical since VF deterioration is permanent [7].

Quantitative assessments for monitoring glaucoma progression are generally based on either global VF indices or pointwise VF measurements. Global indices provide summary measures of VF function but may overlook localized defects. In contrast, pointwise analyses are more sensitive to localized functional changes; however, this higher sensitivity is accompanied by lower specificity [8, 9]. Among pointwise measurements, total deviation values (TDVs) are particularly suitable for longitudinal modeling because they quantify localized sensitivity loss after adjustment for age‐related decline, enabling standardized comparisons across patients and over time.

A wide range of statistical and machine learning approaches has been developed to predict future VF using either global indices or pointwise measurements [10–23]. Among conventional statistical methods, pointwise linear regression (PLR), which fits ordinary linear regression (OLR) independently at each VF location, has been widely used for longitudinal VF analysis because of its simplicity and clinical interpretability [13, 14, 16, 17, 19, 20, 24]. However, OLR does not account for the temporal dependence among repeated VF measurements within each eye. Time series models such as the first‐order autoregressive (AR(1)) model explicitly accounts for this temporal dependence and, therefore, represents an appropriate alternative for longitudinal VF prediction. Despite this theoretical advantage, its application in glaucoma VF prediction has been limited, with only a few previous studies evaluating the AR(1) model for longitudinal VF measurements [24, 25].

More recently, deep learning techniques, particularly recurrent neural networks (RNNs) and long short‐term memory (LSTM) networks have gained attention because of their ability to learn complex temporal patterns directly from sequential VF data [10, 12] .Previous studies have demonstrated that LSTM‐based and hybrid deep learning architectures outperformed conventional regression approaches in VF prediction [10–12, 21, 23]. Machine learning methods have also shown effectiveness in other aspects of VF analysis, including glaucoma detection based on TDVs and pattern deviation values (PDVs), further highlighting their ability to capture complex VF patterns [15]. However, the successful implementation of these approaches requires large, high‐quality datasets and substantial computational resources. Moreover, comparisons among prediction models have predominantly relied on real‐world datasets, in which reliable assessment of VF progression is challenging due to the substantial variability of SAP measurements and the absence of a universally accepted gold standard for defining true progression [26–32]. These limitations make the assessment and comparison of predictive models difficult [33]. Therefore, differences in predictive performance may reflect variations in dataset characteristics rather than genuine methodological superiority.

Computer simulation offers a controlled and reproducible framework in which progression rates, follow‐up schedules, baseline (BL) disease severity, and measurement variability can be specified a priori, and large‐scale longitudinal VF datasets can be generated. These capabilities facilitate objective evaluation and improvement of progression detection and prediction methods and reduce the limitations associated with real‐world data [9, 33, 34]. Early simulation studies [18] generated longitudinal pointwise threshold sensitivities using a simple linear regression model. Although this approach enabled controlled simulation of localized VF deterioration, it did not explicitly model the dependence among repeated measurements within the same eye or the heterogeneity in BL status and progression rates across eyes. More recently, Sabouri et al. [35] introduced a simulation framework based on linear mixed‐effects models (LMMs) for generating longitudinal VF series.

This LMM‐based simulation accounted for between‐eye heterogeneity and dependence among repeated measurements within each eye through random intercepts and random slopes [36, 37]. Although this framework was developed for global MD, pointwise VF series share a similar longitudinal repeated‐measures structure. LMMs provide a controlled longitudinal data‐generating framework that can incorporate systematic progression, between‐eye heterogeneity, within‐eye dependence, and measurement variability. Compared with simple linear regression‐based simulation [18], this approach, therefore, provides a more realistic representation of the longitudinal structure of pointwise VF data.

Despite these advances, several important gaps remain. Previous simulation studies have focused either on global VF indices or on pointwise simulation using simple linear regression. To our knowledge, no existing framework has applied LMM to simulate longitudinal pointwise VFs while accounting for within‐eye dependence and between‐eye heterogeneity. Moreover, comparative evaluations of OLR, AR(1), and deep learning approaches have mainly relied on real‐world datasets, where the true progression trajectory is unknown. Finally, it is unclear whether the increased complexity of deep learning methods translates into superior predictive performance or whether simpler statistical approaches may perform comparably in specific clinical settings.

Accordingly, this study pursued three primary objectives: (1) to develop an LMM‐based framework for simulating longitudinal pointwise TDVs in early‐stage glaucoma, (2) to compare the predictive performance of conventional statistical models (OLR and AR(1)) and a deep learning model (LSTM), and (3) to examine the influence of disease progression rate and the number of examinations on the models′ performance. In addition, a naive BL forecast, assuming the subsequent observation is identical to the most recent measurement, was included as a benchmark for evaluating the more sophisticated prediction models. Based on these findings, we provide recommendations regarding the appropriate application of OLR, AR(1), and LSTM methods for longitudinal VF prediction in glaucoma.

## 2. Materials and Methods

### 2.1. Simulation of Clinical Data

We simulated threshold sensitivity data for 36,000 eyes with a focal inferior nasal defect at BL examination. The simulation period was 5 years with 6‐month intervals, comprising 11 examinations per eye and a total of 396,000 simulated VFs. BL age was set at 40–50 years. Simulation parameters were defined based on the opinion of a glaucoma specialist, who assessed reliable BL VFs with false negative rates < 12% and false positive rates < 4%, as well as fixation losses < 15%. Pointwise VF data were derived from SAP performed with the Humphrey Field Analyzer (Carl Zeiss Meditec), using the 24‐2 test pattern and the SITA Standard strategy. For all analyses and simulations, 52 test locations were used after excluding the two blind‐spot locations from the 24‐2 grid. A BL VF pattern from a glaucoma patient with a focal inferior nasal defect was selected by the glaucoma specialist as the reference pattern for simulation, and age‐matched normative VF data were used to determine the expected interval of threshold sensitivities at each test location. These intervals were used to initialize the BL threshold sensitivity values in the simulated VF data. For the simulation, at locations without glaucomatous damage in the selected BL VF pattern, the lower bound of each interval corresponded to the amount of sensitivity reduction that led to a change in the location from normal to a total deviation probability level of 5%, and the upper bound represented the expected normal sensitivity at that location. For the three locations related to the inferior nasal step defect (locations 27, 28, and 35; Figure 1), sensitivity levels that led to changes in total deviation probability level of 2% and 1% were also determined.

**Figure 1:**
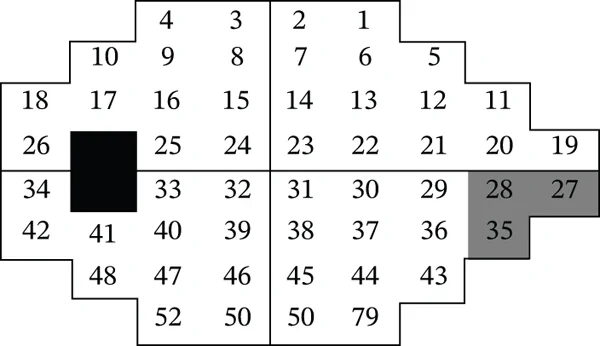
Numbered visual field map (excluding the two blind spots).

Six progression patterns (VF deterioration over time) and three progression rates were defined for the simulation of clinical data in different scenarios, yielding 18 separately simulated datasets, each containing 2000 eyes (Table 1 and Figure 2). Threshold sensitivity values at each of the 52 test locations were generated using an LMM with random intercept and slope parameters. The presence of random intercept and slope in the LMM causes each eye to have its own unique trajectory at BL and over time [37–39]. The equation of this model for threshold sensitivity values over time is written as follows:

(1) TSijk=β0k+b0ik+β1k+b1iktimeij+εijk

**Table 1:** Simulation scenarios based on progression rates and progression patterns.

Progression pattern	Progression rate	Number of deteriorating locations over time
Superior nasal defect (small defect)	−0.25 dB/year (slow)	Deterioration in three new locations with no change in the baseline defect (Figure 2a)
	0.75 dB/year (moderate)	
	−1.5 dB/year (fast)	
Inferior nasal defect (small defect)	−0.25 dB/year (slow)	Deterioration in three new locations with no change in the baseline defect (Figure 2b)
	0.75 dB/year (moderate)	
	−1.5 dB/year (fast)	
Deepening inferior nasal defect and early arcuate damage (medium defect)	−0.25 dB/year (slow)	Deterioration in eight locations: a deepening of the defect at baseline along with five new locations (Figure 2c)
	0.75 dB/year (moderate)	
	−1.5 dB/year (fast)	
Deepening inferior nasal defect and early arcuate damage (medium defect)	−0.25 dB/year (slow)	Deterioration in eight new locations with no change in the baseline defect (Figure 2d)
	0.75 dB/year (moderate)	
	−1.5 dB/year (fast)	
Inferior nasal defect and advanced inferior arcuate damage (large defect)	−0.25 dB/year (slow)	Deterioration in 16 locations: a deepening of the defect at baseline along with 13 new locations (Figure 2e)
	0.75 dB/year (moderate)	
	−1.5 dB/year (fast)	
Inferior nasal defect and inferior arcuate damage with central threatening, and early superior nasal defect (large defect)	−0.25 dB/year (slow)	Deterioration in 19 new locations with no change in the baseline defect (Figure 2f)
	0.75 dB/year (moderate)	
	−1.5 dB/year (fast)	

**Figure 2:**
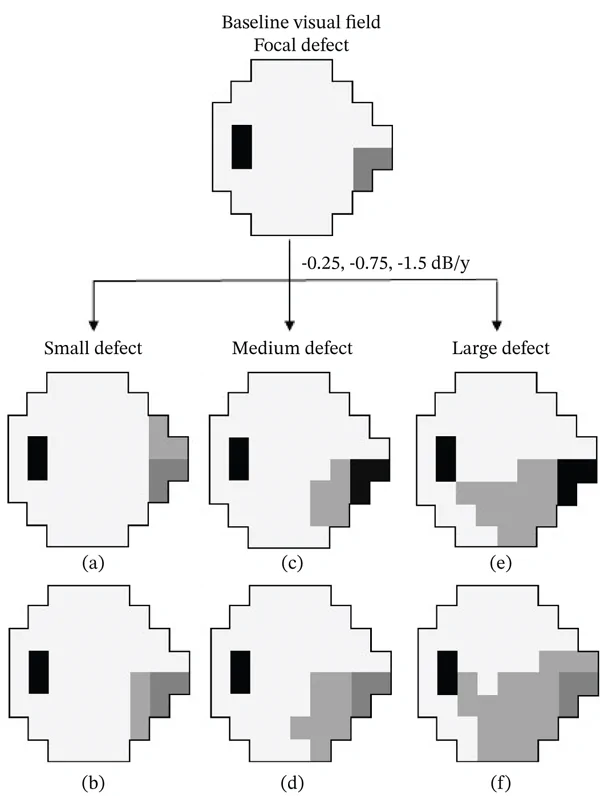
Simulated progression patterns (visual field deterioration over time). Six progression patterns ((a)–(f)) were applied on a baseline visual field with a focal inferior nasal defect in early‐stage glaucoma with different progression rates.

where *T* *S*_*i* *j* *k*_ indicates the threshold sensitivity (dB) for i^th^ eye, j^th^ visit, and k^th^ location. *β*_0*k*_ and *β*_1*k*_ are the fixed intercept and slope parameters, respectively; *b*_0*i* *k*_ and *b*_1*i* *k*_ are the corresponding random intercept and random slope effects;  *t*_*i* *j*_ denotes time; and *ε*_*i* *j* *k*_ represents the error term or noise.

The fixed intercept parameter (*β*_0*k*_) is the population mean BL threshold sensitivity for each location, whereas the random intercept (b_0ik_) represents eye‐specific deviations from this population mean. To determine the fixed and random intercepts, each VF test location was assigned a BL threshold sensitivity interval defined by a glaucoma specialist. Likewise, the fixed slope (*β*_1*k*_) is the population progression rate, and the random slope parameter (*b*_1*i* *k*_) captures eye‐specific variation around this population progression rate. To determine the fixed and random slopes for deteriorating locations, we defined three intervals for the progression rate based on previous studies [35, 40]: slow (0 to −0.5 dB/year), moderate (−0.5 to −1.0 dB/year), and fast (−1.0 to −2.0 dB/year), with corresponding population means of −0.25, −0.75, and −1.50 dB/year, respectively. Locations assumed to remain stable over time, regardless of whether they were healthy or damaged at BL, were assigned an age‐related decline interval of −0.15 to −0.05 dB/year with a population mean of −0.1 dB/year. The random intercept and random slope parameters were generated from a continuous uniform distribution within the predefined intervals, whereas the fixed intercept and slope parameters were set to the corresponding interval means. The error term, or noise component, represents the variability of repeated measurements for each eye. To add noise to the true threshold sensitivity values, a normal distribution was used based on the study by Rabiolo et al. [18]. At each test location, the noisy threshold sensitivity value was obtained by adding a random Monte Carlo value generated from this distribution to reproduce the inherent variability in VF measurements. The mean of the distribution at each test location was considered the simulated true threshold sensitivity, and the standard deviation (SD) was calculated using the function proposed by Gardiner and Crabb [41] as ln (SD) = −0.081 ×sensitivity (dB) +3.27. An eccentricity‐weighting factor, proposed by Spry et al. [9], was added to the SD to increase the variability of peripheral test locations. It is defined as follows:

(2) Eccentricity weighting factor=4.5−i2+4.5−j26.5

where *i* and *j* denote the coordinates of each location in the 10 × 10 grids. Noisy threshold sensitivity values simulated outside the range of 0–36 dB were truncated to 0 and 36, respectively. Finally, TDVs and PDVs were generated for each simulated examination using the “VisualFields” package in RStudio. Table 2 summarizes the simulation setup.

**Table 2:** Parameters of the linear mixed effects model used to simulate threshold sensitivity in patients with early‐stage glaucoma.

Parameter	Simulation setup
Fixed intercept (*β*_0*k*_)	Mean of the expected interval for threshold sensitivity defined by glaucoma specialist at each of the 52 locations.
Random intercept (*b*_0*i* *k*_)	Continuous uniform distribution with parameters −1/2 interval distance of threshold sensitivity defined by glaucoma specialist and +1/2 interval distance of threshold sensitivity defined by glaucoma specialist.
Fixed slope (*β*_1*k*_)	Deteriorating locations: −0.25 dB/year (slow), −0.75 dB/year (moderate), and −1.5 dB/year (fast)
	Stable locations (healthy or damaged at baseline): −0.1 dB/year (age‐related decline)
Random slope (*b*_1*i* *k*_)	Deteriorating locations: continuous uniform distribution with parameters −0.25 dB/year and +0.25 dB/year (slow and moderate); continuous uniform distribution with parameters −0.50 dB/year and +0.50 dB/year (fast)
	Stable locations (healthy or damaged at baseline): continuous uniform distribution with parameters −0.05 dB/year and +0.05 dB/year (age‐related decline)
Error term (*ε*_*i* *j* *k*_)	Normal distribution with mean equal to the simulated true threshold sensitivity value and SD equal to exp(−0.081 × sensitivity (dB) +3.27))

### 2.2. Methods to Predict the VF

For each progression rate, datasets from the six predefined progression patterns were combined into a single dataset, resulting in three simulated datasets representing slow, moderate, and fast progression rates (Figure 3). Four methods were employed to predict future VF examinations (6–11) for each of the three simulated datasets, including a naive BL forecast, OLR, AR(1), and LSTM‐based RNN. For each method, separate models were fitted to predict the successive examinations using all preceding VF examinations as inputs. For example, the model predicting the sixth examination used the BL and the second to fifth examinations as inputs, whereas the model predicting the seventh examination used the BL and the second to sixth examinations. The same sequential prediction framework was applied to all subsequent examinations.

**Figure 3:**
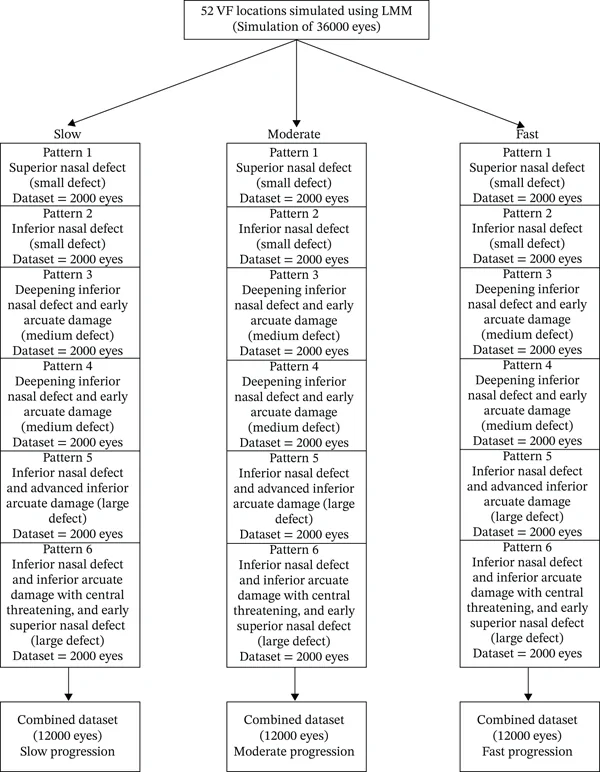
Flowchart of the data preparation process. Eighteen simulated datasets (each containing 2000 eyes) were generated from combinations of six progression patterns and three progression rates. These datasets were then aggregated according to progression rate (slow, moderate, and fast), resulting in three final datasets, each containing 12,000 eyes.

For all analyses, TDVs were used as the primary data representation because they capture age‐adjusted, pointwise measurements of VF sensitivity across the 52 test locations, allowing the models to learn spatial patterns of disease progression. Although threshold sensitivity values were simulated during data generation to derive longitudinal TDVs, they were not used directly for model training or prediction. Similarly, global indices such as MD and pattern standard deviation (PSD) were not used because they summarize the VF into a single value and, therefore, do not preserve the location‐specific information required for predicting future VF changes. Because TDVs were referenced to age‐matched normative values and the same TDV representation and normalization procedure was applied across all models, age‐related adjustment did not favor any prediction method. Consequently, differences in performance reflect the models′ ability to capture temporal and spatial dependencies. Before implementing the models, the simulated data were normalized to the range of [0, 1] using min‐max scaling. Each dataset (12,000 eyes) was randomly divided into training (10,200 eyes; 85%) and testing (1800 eyes; 15%) sets, with 10% of the training data reserved for validation. Following previous studies [10, 12], the LSTM model was trained using the training dataset and evaluated using the test dataset. Its prediction performance was then compared with that of naive BL forecast, OLR, and AR(1) in the test dataset. All analyses were performed in RStudio using the “forecast” package for AR(1) and the “tensorflow” and “keras” packages for the LSTM model.

### 2.3. Naïve BL Forecast

For each eye and each VF location, the predicted TDV at the next examination was assumed to be equal to the TDV observed at the immediately preceding examination:

(3) Y∧t=Yt−1.

This parameter‐free forecasting approach serves as a reference benchmark, reflecting the assumption that no measurable change occurs between consecutive examinations.

### 2.4. OLR

For each VF location and each eye, six independent OLR models (Model 1 to Model 6) were fitted separately (3,744,000 models for each scenario). In each model, TDVs were regressed against time according to

(4) Yt=β0+β1T+et;T=00.512.535,,,⋯,t∗ and t∗=,,⋯,

where *Y*_*t*_ is the TDV at time *t*, *T* denotes the time at each examination from the BL, *β*_0_ and *β*_1_ are the model regression coefficient estimated by the least square (LS) method, and *e*_*t*_ is the random error term.

### 2.5. AR(1) Model

Similar to OLR, six separate AR(1) models (Model 1 to Model 6) were fitted independently for each eye and each VF location (3,744,000 models for each scenario). In each model, TDVs from all examinations preceding the target visit were used to predict the TDV at the target visit.

Because TDV series exhibit a systematic temporal trend due to disease progression, each series was first detrended [25]. Accordingly, the observed series for each eye and VF location was modeled as

(5) Yt=β0+β1T+et;t=1216711,,⋯,t∗− and t∗=,,⋯,.

Here, *Y*_*t*_ denotes the TDV at time *t* (in years from BL), *β*_0_ and *β*_1_ are the intercept and slope estimated by OLS, *T* denotes the time at each examination from the BL, and *e*_*t*_ is the residual error term. The detrended series was obtained as the residuals from the fitted linear model:

(6) Wt=Yt−β∧0+β∧1T; t=1216711,,⋯,t∗− and t∗=,,⋯,.

The AR(1) model was then fitted to the detrended series:

(7) Wt=Ø Wt−1+Ɛt; t=236711,,⋯,t∗ and t∗=,,⋯,.

In this equation, Ø is the autoregressive parameter (−1 < Ø < 1), reflecting the temporal correlation between successive examinations, and *Ɛ*_*t*_ is a white noise error term with zero mean and constant variance. ∅ ∅ was estimated by maximum likelihood estimation (MLE) using all available lag‐1 pairs from the detrended series. The one‐step‐ahead prediction on the detrended scale was obtained as

(8) W∧ttarget=Ø∧Wttarget−1; ttarget=6711,,⋯,

where *t*_*t* *a* *r* *g* *e* *t*_ is the target examination (sixth to eleventh examination), $W_{t_{target}}$is the detrended residual at *t*_*t* *a* *r* *g* *e* *t*_, and $W_{t_{target-1}}$ is the detrended residual at the immediately preceding examination. The final TDV prediction was calculated by restoring the estimated linear trend:

(9) Y∧ttarget=β∧0+β∧1Ttarget+W∧ttarget;ttarget=67112.533.55,,⋯, and Ttarget=,,,⋯,.

As follow‐up examinations were conducted at a 6‐month interval over a 5‐year period, the target examination (sixth to eleventh examination) corresponded to a time point ranging from 2.5 to 5 years from BL, which was substituted into the estimated linear trend in Equation ((9).

### 2.6. RNN

RNNs based on LSTM cells consist of four components: the forget gate, the input gate, the output gate, and the memory cell, which are defined as follows:

(10) Forgetgate=sigmoidWfXt+Whfht−1+bf

(11) Inputgate=sigmoidWiXt+Whiht−1+bi

(12) Outputgate=sigmoidWoXt+Whoht−1+bo

(13) Ct=Ct−1⊗Forgetgate+Inputgate⊗tanh WcXt+Whcht−1+bc

Here, *w*_*f*_, *w*_*i*_, *w*_*o*_, and *w*_*C*_ denote the weights, and *b*_*f*_, *b*_*i*_, *b*_*o*_, and *b*_*C*_ represent the corresponding biases. The sigmoid and tanh functions are nonlinear activation functions applied within the gates. The previous hidden state, *h*_(*t*−1),_ together with the memory cell *C*_(*t*)_, allows the network to retain, discard, and update temporal information through elementwise multiplication (⊗). The hidden state is then computed as

(14) ht=Outputgate⊗tanhCt−1.

The operation of the three gates and the memory cell governs the selective retention, discarding, and updating of temporal information, thereby facilitating the learning of long‐term dependencies [10].

### 2.7. Proposed Method

In our proposed method, a moving “sliding window” of length five was constructed independently for each eye, such that in each window, five consecutive VF examinations were used as model inputs and the subsequent examination was used as model output. As more examinations became available, the number of training windows increased from one (using the first six examinations) to six (using all eleven examinations). For model evaluation, however, only the final (most recent) window for each test eye was used to predict the subsequent VF examination. The few previous studies [10, 12] that used LSTM considered only the first five examinations as inputs for predicting the sixth examination, without applying a sliding window structure due to the limited number of examinations.

To prevent information leakage between training and testing sets, data splitting was performed at the eye level before window construction. The dataset (12,000 eyes) was randomly divided into a training set (10,200 eyes; 85%) and a testing set (1800 eyes; 15%), after which sliding windows were generated independently for each eye. Regardless of the training strategy, the primary objective of all models was to generate the predicted value for the subsequent VF examination. Thus, the main distinction between the models concerned how the model parameters were estimated. Unlike OLR and AR(1), which were fitted separately for each eye and each VF location, only six LSTM models (with the number of training windows increasing from one in Model 1 to six in Model 6) were trained on pooled data from all eyes and locations. Accordingly, OLR and AR(1) represented personalized prediction models with eye‐specific parameter estimation, whereas LSTM represented a population‐level prediction model with a single set of learned weights applied identically to all test eyes.

Each LSTM model received 104 normalized input features comprising 52 TDVs and 52 PDVs. The network architecture was selected empirically for each prediction model. The architecture consisted of two stacked LSTM layers followed by a fully connected output layer with 52 neurons, each corresponding to one VF test location. The first LSTM layer contained 64 hidden units, and the second layer varied according to the progression scenario and prediction model (slow: 50–140 units; moderate: 30–120 units; and fast: 60–170 units). The final output consisted of the predicted TDVs at all 52 test locations.

Models were trained using the Adam optimizer (learning rate = 0.0001) with mean squared error (MSE) as the loss function, a batch size of 512, and a maximum of 100 epochs. To reduce overfitting, early stopping was applied based on validation loss, with a patience of four epochs and a minimum improvement threshold of 0.001, restoring the weights corresponding to the best validation performance. L2 weight regularization was applied to the output layer, and in some architectures, a dropout layer (rate = 0.1) was inserted between the final LSTM and output layers. Training continued until the early stopping criterion was satisfied. The complete training hyperparameters are summarized in Table 3.

**Table 3:** Summary of model training hyperparameters.

Parameter	Optimizer	Learning rate	Loss function	Batch size	Maximum epochs	Validation split	Early stopping monitor	Dropout
Value/description	Adam	0.0001	MSE	512	100	10% of training data	Validation loss	0.1

### 2.8. Model Evaluation Metrics

To evaluate the prediction performance of the models, two accuracy metrics were used, including the root mean squared error (RMSE) and the pointwise mean absolute error (PMAE) of TDV [10–12, 42]. The RMSE for each eye was computed using the following formula:

(15) RMSE=∑n=152true TDVn−predicted TDVn252

where *n* denotes each test location in the VF examination. The PMAE across all eyes for each VF test location was computed using the following formula:

(16) PMAEn=∑i=1number of eyestrue TDVi,n−predicted TDVi,nnumber of eyes

where *i* represents the eye, *n* represents the test location of VF examination, and TDV_i,n_ represents TDV of eye *i*, test location *n*. RMSE and PMAE metrics were calculated for naive BL forecast, LSTM, AR(1), and OLR. To compare the accuracy metrics among four methods, repeated measures one‐way analysis of variance was used, followed by paired *t*‐tests with Bonferroni correction for pairwise comparisons. Cohen′s *d* or effect size was calculated for pairwise comparisons. Model evaluation was performed using SPSS software Version 23.0.

## 3. Results

Tables 4, 5, and 6 summarize the overall RMSE and PMAE prediction errors for the slow, moderate, and fast progression rate scenarios, respectively. Multiple comparisons among the four models were significant across all progression rate scenarios. In the slow progression rate scenario, pairwise comparisons showed that the RMSE and PMAE for LSTM at the sixth examination were 3.33 ± 0.37 dB and 2.61 ± 0.69 dB, respectively, which were significantly lower than those of AR(1) and OLR (Cohen′s *d* > 0.8). As the number of examinations increased, the prediction errors for LSTM gradually increased, whereas those for AR(1) decreased, so that, at the eleventh examination, AR(1) performed slightly better than LSTM in RMSE (3.49 ± 0.44 dB vs. 3.56 ± 0.40 dB; Cohen′s *d* = 0.20) and moderately better than LSTM in PMAE (2.66 ± 0.82 dB vs. 2.79 ± 0.71 dB; Cohen′s *d* = 0.63). OLR and naive BL forecast consistently exhibited higher prediction errors than both LSTM and AR(1) in all examinations, with large effect sizes (Cohen′s *d* > 0.8). Compared with OLR, the naive BL forecast also yielded higher prediction errors from the seventh examination onward, with Cohen′s *d* ranging from 0.24 to 1.55 for RMSE and from 1.62 to 3.48 for PMAE (Table 4)

**Table 4:** Comparison among OLR, LSTM, and AR(1) models in slow progression rate scenario.

6^th^ examination	OLR	AR(1)	LSTM	BL	Cohen′s *d*					
					OLR vs. AR(1)	OLR vs. LSTM	AR(1) vs. LSTM	BL vs. AR(1)	BL vs. OLR	BL vs. LSTM
RMSE (dB)	$\begin{array}{c} 4.500.53\pm \end{array}$	$\begin{array}{c} 3.710.47\pm \end{array}$	$\begin{array}{c} 3.330.37\pm \end{array}$	$\begin{array}{c} 4.410.53\pm \end{array}$	1.43	2.24	0.90	1.20	0.24	2.16
PMAE (dB)	$\begin{array}{c} 3.461.07\pm \end{array}$	$\begin{array}{c} 2.810.84\pm \end{array}$	$\begin{array}{c} 2.610.69\pm \end{array}$	$\begin{array}{c} 3.381.04\pm \end{array}$	2.76	2.09	0.98	2.66	1.62	1.99
7^th^ examination	OLR	AR(1)	LSTM	BL	Cohen′s *d*					
					OLR vs. AR(1)	OLR vs. LSTM	AR(1) vs. LSTM	BL vs. AR(1)	BL vs. OLR	BL vs. LSTM
RMSE (dB)	$\begin{array}{c} 4.280.49\pm \end{array}$	$\begin{array}{c} 3.640.46\pm \end{array}$	$\begin{array}{c} 3.380.38\pm \end{array}$	$\begin{array}{c} 4.430.51\pm \end{array}$	1.32	1.94	0.63	1.42	0.38	2.15
PMAE (dB)	$\begin{array}{c} 3.291.02\pm \end{array}$	$\begin{array}{c} 2.760.87\pm \end{array}$	$\begin{array}{c} 2.640.73\pm \end{array}$	$\begin{array}{c} 3.401.05\pm \end{array}$	3.26	2.04	0.69	3.21	1.87	2.14
8^th^ examination	OLR	AR(1)	LSTM	BL	Cohen′s *d*					
					OLR vs. AR(1)	OLR vs. LSTM	AR(1) vs. LSTM	BL vs. AR(1)	BL vs. OLR	BL vs. LSTM
RMSE (dB)	$\begin{array}{c} 4.110.49\pm \end{array}$	$\begin{array}{c} 3.570.45\pm \end{array}$	$\begin{array}{c} 3.440.39\pm \end{array}$	$\begin{array}{c} 4.450.52\pm \end{array}$	1.20	1.51	0.13	1.61	0.88	1.99
PMAE (dB)	$\begin{array}{c} 3.160.96\pm \end{array}$	$\begin{array}{c} 2.720.81\pm \end{array}$	$\begin{array}{c} 2.680.69\pm \end{array}$	$\begin{array}{c} 3.411.05\pm \end{array}$	2.82	1.48	0.18	2.79	2.47	1.80
9^th^ examination	OLR	AR(1)	LSTM	BL	Cohen′s *d*					
					OLR vs. AR(1)	OLR vs. LSTM	AR(1) vs. LSTM	BL vs. AR(1)	BL vs. OLR	BL vs. LSTM
RMSE (dB)	$\begin{array}{c} 4.010.49\pm \end{array}$	$\begin{array}{c} 3.520.44\pm \end{array}$	$\begin{array}{c} 3.500.39\pm \end{array}$	$\begin{array}{c} 4.460.55\pm \end{array}$	1.21	1.21	0.04	1.75	1.11	1.84
PMAE (dB)	$\begin{array}{c} 3.090.95\pm \end{array}$	$\begin{array}{c} 2.680.80\pm \end{array}$	$\begin{array}{c} 2.750.71\pm \end{array}$	$\begin{array}{c} 3.421.02\pm \end{array}$	2.58	1.06	0.28	3.14	3.48	1.74
10^th^ examination	OLR	AR(1)	LSTM	BL	Cohen′s *d*					
					OLR vs. AR(1)	OLR vs. LSTM	AR(1) vs. LSTM	BL vs. AR(1)	BL vs. OLR	BL vs. LSTM
RMSE (dB)	$\begin{array}{c} 3.930.48\pm \end{array}$	$\begin{array}{c} 3.500.44\pm \end{array}$	$\begin{array}{c} 3.520.39\pm \end{array}$	$\begin{array}{c} 4.500.54\pm \end{array}$	1.11	0.99	0.07	1.91	1.38	1.85
PMAE (dB)	$\begin{array}{c} 3.030.92\pm \end{array}$	$\begin{array}{c} 2.670.81\pm \end{array}$	$\begin{array}{c} 2.760.75\pm \end{array}$	$\begin{array}{c} 3.451.06\pm \end{array}$	3.00	0.95	0.39	3.06	2.99	1.77
11^th^ examination	OLR	AR(1)	LSTM	BL	Cohen′s *d*					
					OLR vs. AR(1)	OLR vs. LSTM	AR(1) vs. LSTM	BL vs. AR(1)	BL vs. OLR	BL vs. LSTM
RMSE (dB)	$\begin{array}{c} 3.860.47\pm \end{array}$	$\begin{array}{c} 3.490.44\pm \end{array}$	$\begin{array}{c} 3.560.40\pm \end{array}$	$\begin{array}{c} 4.520.55\pm \end{array}$	1.01	0.76	0.20	1.89	1.55	1.81
PMAE (dB)	$\begin{array}{c} 2.970.91\pm \end{array}$	$\begin{array}{c} 2.660.82\pm \end{array}$	$\begin{array}{c} 2.790.71\pm \end{array}$	$\begin{array}{c} 3.461.07\pm \end{array}$	3.09	0.67	0.63	3.18	3.06	1.65

Abbreviations: AR(1), first‐order autoregressive; BL, naive baseline forecast; LSTM, long short‐term memory; OLR, ordinary linear regression; PMAE, pointwise mean absolute error; RMSE, root mean squared error; SD, standard deviation.

**Table 5:** Comparison among OLR, LSTM, and AR(1) models in moderate progression rate scenario.

6^th^ examination	OLR	AR(1)	LSTM	BL	Cohen′s *d*					
					OLR vs. AR(1)	OLR vs. LSTM	AR(1) vs. LSTM	BL vs. AR(1)	BL vs. OLR	BL vs. LSTM
RMSE (dB)	$\begin{array}{c} 4.580.56\pm \end{array}$	$\begin{array}{c} 3.790.50\pm \end{array}$	$\begin{array}{c} 3.510.38\pm \end{array}$	$\begin{array}{c} 4.500.55\pm \end{array}$	1.41	2.00	0.61	1.15	0.21	1.88
PMAE (dB)	$\begin{array}{c} 3.531.07\pm \end{array}$	$\begin{array}{c} 2.870.88\pm \end{array}$	$\begin{array}{c} 2.750.75\pm \end{array}$	$\begin{array}{c} 3.441.07\pm \end{array}$	3.21	1.80	0.41	2.87	1.98	1.63
7^th^ examination	OLR	AR(1)	LSTM	BL	Cohen′s *d*					
					OLR vs. AR(1)	OLR vs. LSTM	AR(1) vs. LSTM	BL vs. AR(1)	BL vs. OLR	BL vs. LSTM
RMSE (dB)	$\begin{array}{c} 4.350.54\pm \end{array}$	$\begin{array}{c} 3.730.48\pm \end{array}$	$\begin{array}{c} 3.640.39\pm \end{array}$	$\begin{array}{c} 4.530.58\pm \end{array}$	1.21	1.41	0.21	1.38	0.46	1.70
PMAE (dB)	$\begin{array}{c} 3.341.03\pm \end{array}$	$\begin{array}{c} 2.820.89\pm \end{array}$	$\begin{array}{c} 2.850.79\pm \end{array}$	$\begin{array}{c} 3.471.08\pm \end{array}$	3.35	1.31	0.07	3.17	1.90	1.55
8^th^ examination	OLR	AR(1)	LSTM	BL	Cohen′s *d*					
					OLR vs. AR(1)	OLR vs. LSTM	AR(1) vs. LSTM	BL vs. AR(1)	BL vs. OLR	BL vs. LSTM
RMSE (dB)	$\begin{array}{c} 4.210.52\pm \end{array}$	$\begin{array}{c} 3.690.48\pm \end{array}$	$\begin{array}{c} 3.750.41\pm \end{array}$	$\begin{array}{c} 4.580.56\pm \end{array}$	1.12	0.94	0.13	1.58	0.95	1.51
PMAE (dB)	$\begin{array}{c} 3.230.99\pm \end{array}$	$\begin{array}{c} 2.800.86\pm \end{array}$	$\begin{array}{c} 2.940.80\pm \end{array}$	$\begin{array}{c} 3.501.10\pm \end{array}$	2.99	0.76	0.43	2.78	2.18	1.19
9^th^ examination	OLR	AR(1)	LSTM	BL	Cohen′s *d*					
					OLR vs. AR(1)	OLR vs. LSTM	AR(1) vs. LSTM	BL vs. AR(1)	BL vs. OLR	BL vs. LSTM
RMSE (dB)	$\begin{array}{c} 4.130.51\pm \end{array}$	$\begin{array}{c} 3.640.47\pm \end{array}$	$\begin{array}{c} 3.810.41\pm \end{array}$	$\begin{array}{c} 4.620.57\pm \end{array}$	1.13	0.65	0.40	1.71	1.15	1.42
PMAE (dB)	$\begin{array}{c} 3.160.99\pm \end{array}$	$\begin{array}{c} 2.760.85\pm \end{array}$	$\begin{array}{c} 2.990.79\pm \end{array}$	$\begin{array}{c} 3.531.10\pm \end{array}$	2.57	0.37	0.62	2.90	2.98	1.00
10^th^ examination	OLR	AR(1)	LSTM	BL	Cohen′s *d*					
					OLR vs. AR(1)	OLR vs. LSTM	AR(1) vs. LSTM	BL vs. AR(1)	BL vs. OLR	BL vs. LSTM
RMSE (dB)	$\begin{array}{c} 4.030.50\pm \end{array}$	$\begin{array}{c} 3.630.48\pm \end{array}$	$\begin{array}{c} 3.830.43\pm \end{array}$	$\begin{array}{c} 4.630.58\pm \end{array}$	0.95	0.95	0.47	1.77	1.39	1.49
PMAE (dB)	$\begin{array}{c} 3.090.94\pm \end{array}$	$\begin{array}{c} 2.760.86\pm \end{array}$	$\begin{array}{c} 3.000.81\pm \end{array}$	$\begin{array}{c} 3.551.09\pm \end{array}$	3.16	0.25	0.72	3.26	2.94	1.19
11^th^ examination	OLR	AR(1)	LSTM	BL	Cohen′s *d*					
					OLR vs. AR(1)	OLR vs. LSTM	AR(1) vs. LSTM	BL vs. AR(1)	BL vs. OLR	BL vs. LSTM
RMSE (dB)	$\begin{array}{c} 4.010.50\pm \end{array}$	$\begin{array}{c} 3.630.47\pm \end{array}$	$\begin{array}{c} 4.030.42\pm \end{array}$	$\begin{array}{c} 4.700.59\pm \end{array}$	0.95	0.06	0.98	1.94	1.57	1.15
PMAE (dB)	$\begin{array}{c} 3.070.94\pm \end{array}$	$\begin{array}{c} 2.760.85\pm \end{array}$	$\begin{array}{c} 3.160.97\pm \end{array}$	$\begin{array}{c} 3.591.12\pm \end{array}$	3.05	0.13	0.63	2.99	2.75	0.57

Abbreviations: AR(1), first‐order autoregressive; BL, naive baseline forecast; LSTM, long short‐term memory; OLR, ordinary linear regression; PMAE, pointwise mean absolute error; RMSE, root mean squared error; SD, standard deviation.

**Table 6:** Comparison among OLR, LSTM, and AR(1) models in fast progression rate scenario.

6^th^ examination	OLR	AR(1)	LSTM	BL	Cohen′s *d*					
					OLR vs. AR(1)	OLR vs. LSTM	AR(1) vs. LSTM	BL vs. AR(1)	BL vs. OLR	BL vs. LSTM
RMSE (dB)	$\begin{array}{c} 4.710.61\pm \end{array}$	$\begin{array}{c} 3.960.56\pm \end{array}$	$\begin{array}{c} 3.740.44\pm \end{array}$	$\begin{array}{c} 4.630.60\pm \end{array}$	1.29	1.65	0.46	1.08	0.23	1.57
PMAE (dB)	$\begin{array}{c} 3.601.17\pm \end{array}$	$\begin{array}{c} 2.970.94\pm \end{array}$	$\begin{array}{c} 2.910.81\pm \end{array}$	$\begin{array}{c} 3.521.13\pm \end{array}$	2.47	1.39	0.17	2.67	1.17	1.35
7^th^ examination	OLR	AR(1)	LSTM	BL	Cohen′s *d*					
					OLR vs. AR(1)	OLR vs. LSTM	AR(1) vs. LSTM	BL vs. AR(1)	BL vs. OLR	BL vs. LSTM
RMSE (dB)	$\begin{array}{c} 4.530.59\pm \; \end{array}$	$\begin{array}{c} 3.930.55\pm \end{array}$	$\begin{array}{c} 3.860.45\pm \end{array}$	$\begin{array}{c} 4.730.62\pm \end{array}$	1.17	1.21	0.14	1.31	0.48	1.52
PMAE (dB)	$\begin{array}{c} 3.461.12\pm \end{array}$	$\begin{array}{c} 2.950.97\pm \end{array}$	$\begin{array}{c} 3.000.86\pm \end{array}$	$\begin{array}{c} 3.601.18\pm \end{array}$	2.90	1.07	0.19	2.80	1.76	1.31
8^th^ examination	OLR	AR(1)	LSTM	BL	Cohen′s *d*					
					OLR vs. AR(1)	OLR vs. LSTM	AR(1) vs. LSTM	BL vs. AR(1)	BL vs. OLR	BL vs. LSTM
RMSE (dB)	$\begin{array}{c} 4.410.58\pm \; \end{array}$	$\begin{array}{c} 3.890.54\pm \end{array}$	$\begin{array}{c} 4.040.47\pm \; \end{array}$	$\begin{array}{c} 4.810.64\pm \end{array}$	1.06	0.67	0.31	1.54	0.92	1.25
PMAE (dB)	$\begin{array}{c} 3.361.10\pm \; \end{array}$	$\begin{array}{c} 2.910.95\pm \end{array}$	$\begin{array}{c} 3.130.91\pm \; \end{array}$	$\begin{array}{c} 3.641.21\pm \end{array}$	2.58	0.47	0.58	2.71	2.21	0.95
9^th^ examination	OLR	AR(1)	LSTM	BL	Cohen′s *d*					
					OLR vs. AR(1)	OLR vs. LSTM	AR(1) vs. LSTM	BL vs. AR(1)	BL vs. OLR	BL vs. LSTM
RMSE (dB)	$\begin{array}{c} 4.340.60\pm \end{array}$	$\begin{array}{c} 3.880.59\pm \end{array}$	$\begin{array}{c} 4.310.50\pm \; \end{array}$	$\begin{array}{c} 4.880.67\pm \end{array}$	0.93	0.05	0.85	1.60	1.16	0.91
PMAE (dB)	$\begin{array}{c} 3.291.09\pm \; \end{array}$	$\begin{array}{c} 2.900.98\pm \end{array}$	$\begin{array}{c} 3.361.00\pm \; \end{array}$	$\begin{array}{c} 3.691.22\pm \end{array}$	3.12	0.12	0.93	3.24	2.90	0.54
10^th^ examination	OLR	AR(1)	LSTM	BL	Cohen′s *d*					
					OLR vs. AR(1)	OLR vs. LSTM	AR(1) vs. LSTM	BL vs. AR(1)	BL vs. OLR	BL vs. LSTM
RMSE (dB)	$\begin{array}{c} 4.290.59\pm \; \end{array}$	$\begin{array}{c} 3.880.57\pm \end{array}$	$\begin{array}{c} 4.440.53\pm \; \end{array}$	$\begin{array}{c} 4.950.70\pm \end{array}$	0.88	0.27	1.12	1.67	1.35	0.76
PMAE (dB)	$\begin{array}{c} 3.261.07\pm \; \end{array}$	$\begin{array}{c} 2.900.97\pm \end{array}$	$\begin{array}{c} 3.431.14\pm \; \end{array}$	$\begin{array}{c} 3.741.25\pm \end{array}$	3.13	0.25	0.86	2.92	2.59	0.45
11^th^ examination	OLR	AR(1)	LSTM	BL	Cohen′s *d*					
					OLR vs. AR(1)	OLR vs. LSTM	AR(1) vs. LSTM	BL vs. AR(1)	BL vs. OLR	BL vs. LSTM
RMSE (dB)	$\begin{array}{c} 4.300.62\pm \; \end{array}$	$\begin{array}{c} 3.890.58\pm \end{array}$	$\begin{array}{c} 4.670.54\pm \; \end{array}$	$\begin{array}{c} 5.040.73\pm \end{array}$	0.89	0.63	1.47)	1.80	1.50	0.53
PMAE (dB)	$\begin{array}{c} 3.251.10\pm \; \end{array}$	$\begin{array}{c} 2.900.99\pm \end{array}$	$\begin{array}{c} 3.651.19\pm \; \end{array}$	$\begin{array}{c} 3.801.27\pm \end{array}$	2.93	0.52	0.97)	3.08	3.01	0.17

Abbreviations: AR(1), first‐order autoregressive; BL naive baseline forecast; LSTM, long short‐term memory; OLR, ordinary linear regression; PMAE, pointwise mean absolute error; RMSE, root mean squared error; SD, standard deviation.

Similarly, in the moderate progression rate scenario, at the sixth examination, the lowest RMSE (3.51 ± 0.38 dB) and PMAE (2.75 ± 0.75 dB) were observed for LSTM. However, the effect size between LSTM and AR(1) was medium for RMSE (Cohen′s *d* = 0.61) and small for PMAE (Cohen′s *d* = 0.41). As the number of examinations increased, LSTM prediction errors increased. From the eighth examination onward, AR(1) performed better than LSTM in PMAE (Cohen′s *d* = 0.43–0.72), and from the ninth examination onward, it also performed better than LSTM in RMSE (Cohen′s *d* ranging from 0.40 to 0.98). At the eleventh examination, AR(1) achieved the lowest RMSE and PMAE, which were 3.63 ± 0.47 dB and 2.76 ± 0.85 dB, respectively. Additionally, the differences between OLR and LSTM decreased as the number of examinations increased, with negligible effect sizes observed for both RMSE and PMAE at the eleventh examination. Across all examinations, AR(1) and LSTM outperformed the naive BL forecast (Cohen′s *d* > 0.8), and OLR surpassed it from the seventh examination onward (Table 5).

In the fast progression rate scenario at the sixth examination, the RMSE for LSTM and AR(1) were 3.74 ± 0.44 dB and 3.96 ± 0.56 dB, respectively, corresponding to a small effect size (Cohen′s *d* = 0.46). At the seventh examination, the difference between LSTM and AR(1) was negligible (Cohen′s *d* = 0.14). In subsequent examinations with Cohen′s *d* increasing from 0.31 to 1.47, the difference between the two models increased, and AR(1) performed better than LSTM. Additionally, from the eighth examination onward, AR(1) exhibited a lower PMAE than LSTM, with Cohen′s *d* increasing from 0.58 at the eighth examination to 0.97 at the eleventh examination. As the number of examinations increased, the prediction errors for OLR decreased compared with those for LSTM, so that, at the eleventh examination, OLR achieved RMSE and PMAE of 4.30 ± 0.62 dB and 3.25 ± 1.10 dB, respectively. The differences between OLR and LSTM corresponded to medium effect sizes (Cohen′s *d* = 0.63 for RMSE and 0.52 for PMAE). Across all examinations, the RMSE and PMAE for AR(1) were lower than those of OLR and naive BL forecast, with consistently large effect sizes (Cohen′s *d* > 0.8). OLR also outperformed the naive BL forecast from the seventh examination onward, with an increasingly larger difference over subsequent examinations .In contrast, LSTM outperformed the naive BL forecast at earlier examinations, but its advantage gradually diminished as the number of examinations increased (Cohen′s *d* for RMSE decreased from 1.57 at the sixth examination to 0.53 at the eleventh examination) (Table 6).

Table 7 describes the overall RMSE across the VF sectors for the sixth examination in the slow progression rate scenario. As in the study by Kim et al. [10], the prediction error was compared among the six sectors of the 24‐2 VF proposed by Garway‐Heath et al. [43], as well as central and peripheral zones. The LSTM prediction errors were lower than those of AR(1) and OLR across all sections. Effect sizes for LSTM and AR(1) comparisons were small to medium in most sections (Cohen′s *d* = 0.26–0.52). However, a larger effect in the peripheral zone (Cohen′s *d* = 0.86) and a negligible effect in the nasal sector (Cohen′s *d* = 0.07) were observed. In contrast, effect sizes for OLR and LSTM comparisons (Cohen′s *d* = 0.63–2.06) and OLR and AR(1) comparisons (Cohen′s *d* = 0.50–1.29) were medium to large across all sections. The naive BL forecast showed higher RMSE than both LSTM and AR(1) across all VF sectors and zones, whereas the differences between OLR and naive BL forecast were small.

**Table 7:** Comparison among OLR, LSTM, and AR(1) models in the six Garway‐Heath sectors and peripheral and central zones of the 24–2 visual field test for the sixth examination in the slow progression rate scenario.

	Prediction error (RMSE, dB), mean ± SD				(Cohen′s *d*)					
	OLR	LSTM	AR(1)	BL	AR(1) vs. LSTM	AR(1) vs. OLR	OLR vs. LSTM	BL vs. AR(1)	BL vs. OLR	BL vs. LSTM
Superotemporal	$\begin{array}{c} 4.781.28\pm \end{array}$	$\begin{array}{c} 3.570.93\pm \end{array}$	$\begin{array}{c} 3.911.13\pm \end{array}$	$\begin{array}{c} 4.671.25\pm \end{array}$	0.33	0.69	0.98	0.57	0.12	0.92
Superonasal	$\begin{array}{c} 4.020.79\pm \end{array}$	$\begin{array}{c} 3.030.55\pm \end{array}$	$\begin{array}{c} 3.300.67\pm \end{array}$	$\begin{array}{c} 3.940.78\pm \end{array}$	0.41	0.92	1.25	0.78	0.14	1.19
Nasal	$\begin{array}{c} 2.920.84\pm \end{array}$	$\begin{array}{c} 2.360.64\pm \end{array}$	$\begin{array}{c} 2.420.74\pm \end{array}$	$\begin{array}{c} 2.860.81\pm \end{array}$	0.07	0.60	0.63	0.50	0.10	0.58
Inferonasal	$\begin{array}{c} 4.521.45\pm \end{array}$	$\begin{array}{c} 3.271.00\pm \end{array}$	$\begin{array}{c} 3.651.23\pm \end{array}$	$\begin{array}{c} 4.451.43\pm \end{array}$	0.35	0.60	0.91	0.52	0.06	0.89
Inferotemporal	$\begin{array}{c} 5.091.23\pm \end{array}$	$\begin{array}{c} 3.720.85\pm \end{array}$	$\begin{array}{c} 4.231.11\pm \end{array}$	$\begin{array}{c} 4.981.23\pm \end{array}$	0.52	0.67	1.16	0.55	0.12	1.08
Temporal	$\begin{array}{c} 4.211.51\pm \end{array}$	$\begin{array}{c} 3.111.09\pm \end{array}$	$\begin{array}{c} 3.441.30\pm \end{array}$	$\begin{array}{c} 4.101.47\pm \end{array}$	0.28	0.50	0.74	0.40	0.10	1.44
Central	$\begin{array}{c} 3.080.64\pm \end{array}$	$\begin{array}{c} 2.400.48\pm \end{array}$	$\begin{array}{c} 2.540.55\pm \end{array}$	$\begin{array}{c} 3.020.61\pm \end{array}$	0.26	0.86	1.03	0.75	0.13	0.99
Peripheral	$\begin{array}{c} 4.830.63\pm \end{array}$	$\begin{array}{c} 3.550.44\pm \end{array}$	$\begin{array}{c} 3.980.56\pm \end{array}$	$\begin{array}{c} 4.740.63\pm \end{array}$	0.86	1.29	2.06	1.09	0.22	2.00

Abbreviations: AR(1), first‐order autoregressive; BL naive baseline forecast; LSTM long short‐term memory; OLR ordinary linear regression; RMSE, root mean squared error; SD, standard deviation.

Figure 4 illustrates the overall RMSE ± SD and PMAE ± SD of OLR, LSTM, AR(1), and naive BL forecast across all scenarios for examinations 6–11, whereas Figure 5 shows the number of eyes categorized based on the RMSE prediction error for the sixth examination in the slow progression rate scenario. For LSTM, most eyes exhibited prediction errors of 2–3 dB or 3–4 dB. Specifically, 347 eyes had prediction errors of 2–3 dB, and 1364 eyes had prediction errors of 3–4 dB. For OLR, 299 eyes had prediction errors of 3–4 dB, 1187 eyes had prediction errors of 4–5 dB, and 304 eyes had prediction errors of 5–6 dB. For AR(1), 102 eyes had prediction errors of 2–3 dB, 1242 eyes had prediction errors of 3–4 dB, and 441 eyes had prediction errors of 4–5 dB. For naive BL forecast, 390 eyes had prediction errors of 3–4 dB, 1171 eyes had prediction errors of 4–5 dB, and 227 eyes had prediction errors of 5–6 dB.

**Figure 4:**
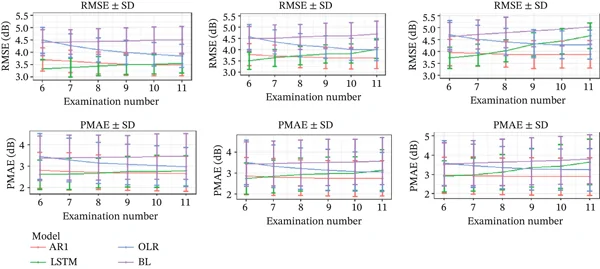
The overall RMSE and PMAE values obtained by predicting the TD values for the 6^th^–11^th^ examinations. The plot shows the RMSE and PMAE values obtained by predicting the TD values. Error bars indicate standard deviations, as shown in Tables 4, 5 and 6. RMSE, root mean squared error; PMAE, pointwise mean absolute error; TD, total deviation.

**Figure 5:**
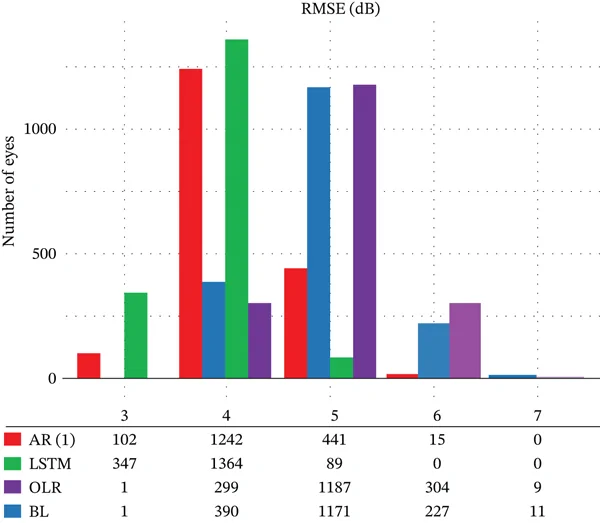
Number of eyes categorized according to the prediction error of RMSE for the sixth examination in the slow progression rate scenario. RMSE, root mean squared error.

Figure 6 indicates the PMAE across 52 VF locations for the sixth examination in the slow progression rate scenario. LSTM had significantly lower prediction errors compared with AR(1) (red dots), OLR (blue dots), and naive BL forecast (green dots) in most locations.

**Figure 6:**
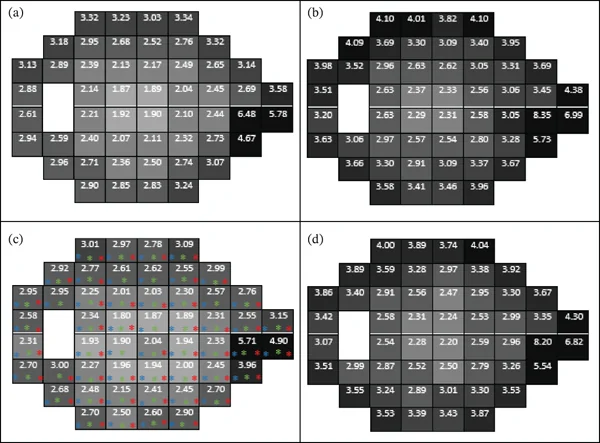
Pointwise mean absolute error (PMAE) of predicted total deviation value (TDV) for the sixth examination in the slow progression rate scenario. The darker colors indicate higher error. The darker color indicates higher error. Red dots indicate significant differences between AR(1) and LSTM; blue dots indicate significant differences between LSTM and OLR; green dots indicate significant differences between BL and LSTM (paired *t*‐test). OLR, ordinary linear regression; LSTM, long short‐term memory; AR(1), first‐order auto regressive; BL, naive baseline forecast.

## 4. Discussion

VF prediction is important in patients with glaucoma because it allows clinicians to assess future VF function and helps them decide on optimal treatment and follow‐up schedules. It is challenging to choose a model that is both interpretable and capable of predicting complex patterns incorporating noise in VF data.

Although deep learning methods such as RNNs have shown high accuracy for predicting complex patterns in VF data, their ambiguity in interpretation (often referred to as a black box) and their need for large training datasets make them difficult to implement in clinical settings. Conversely, conventional statistical methods, such as OLR, are more interpretable but are unable to account for temporal dependence among repeated VF measurements. AR(1) models address this limitation by explicitly modeling serial correlation while retaining the interpretability and simplicity of a statistical approach. Accordingly, the present study compared personalized statistical models (OLR and AR(1)) with a population‐level deep learning model (LSTM) to predict longitudinal VF measurements in glaucoma. For this purpose, different progression rate and pattern scenarios were designed, and the LMM approach was used to realistically simulate pointwise VF measurements over time. The performance of the models was then evaluated under slow, moderate, and fast progression scenarios with varying numbers of examinations.

Across all progression scenarios, AR(1) consistently achieved significantly lower prediction errors than OLR. This result is expected because, unlike OLR, AR(1) explicitly accounts for temporal dependence between consecutive VF measurements. Although most VF locations were stable or exhibited slow progression, AR(1) outperformed the naive BL forecast, particularly as the number of examinations increased. These results indicate that the superior performance of AR(1) cannot be explained solely by the inherent stability of the VF series; rather, modeling temporal dependence captures meaningful predictive information beyond persistence alone.

Several studies [13, 14, 19, 20] have reported that OLR demonstrates satisfactory performance for VF prediction in glaucoma. More recently, however, increasingly sophisticated statistical and machine learning methods have been proposed to improve predictive accuracy [10–12, 21, 23–25]. For example, Childress et al. [24] evaluated the OLR, AR(1), and first‐order irregular autoregressive [IAR(1)] models and found that both autoregressive models showed better predictive performance than OLR. In another related study, Naderi et al. [25] examined the predictive performance of AR(1), OLR, exponential, and polynomial models in patients with glaucoma. AR(1) showed superior pointwise predictive accuracy, particularly in advanced glaucoma, where it had significantly lower RMSE and PMAE than the other models. Consistent with these findings, AR(1) also significantly outperformed OLR across all VF progression rate scenarios in the present study.

Murata et al. [11] compared variational Bayesian linear regression (VBLR) with OLR for predicting TDVs by varying the number of input VF data from 2 to 10. VBLR exhibited more accurate predictions than OLR (*p* < 0.001), with an overall RMSE of 4.5 ± 2.4 dB when predicting the sixth VF examination. Although the LSTM model in the present study yielded lower RMSE values across all simulated progression scenarios, direct comparison between the two studies should be interpreted cautiously because of substantial differences in the study design, simulation framework, and evaluation methodology. Consequently, our findings do not establish the superiority of LSTM over the VBLR approach.

Similarly, Park et al. [12] demonstrated that LSTM significantly outperformed OLR when predicting the sixth VF examination and attributed this improvement to the incorporation of reliability indices (false positive rates, false negative rates, and fixation losses) as additional model inputs. Kim et al. [10] likewise reported that both bidirectional gated recurrent unit (bi‐GRU) and LSTM had superior predictive performance compared with OLR when predicting the sixth VF examination. Our findings are consistent with those reported by Park et al. [12] and Kim et al. [10]. The present study showed that the population‐level LSTM model achieved significantly better predictive performance than both personalized statistical models (OLR and AR(1)) at the sixth examination across all progression rate scenarios. This is likely because only five previous examinations were available for estimating the parameters of the personalized models. Under these conditions, OLR and AR(1) must estimate regression and autoregressive parameters, respectively, from a small number of longitudinal observations, which may lead to greater uncertainty in parameter estimation. In contrast, the LSTM model benefits from population‐level learning by leveraging information from all training eyes to estimate a common set of model parameters, which may allow it to generate more robust predictions when only a limited number of previous examinations are available.

Contrary to expectations, increasing the number of examinations did not improve the performance of LSTM compared with AR(1) in the present study. Rather, AR(1) obtained superior predictive accuracy, particularly under moderate and fast progression scenarios. These results should be interpreted in the context of the distinction between personalized and population‐level modeling frameworks. Because AR(1) was estimated separately for each eye, additional examinations progressively provided more information for estimating eye‐specific temporal dynamics and autoregressive parameters. This enhanced the model′s ability to characterize each eye′s longitudinal progression trajectory. In contrast, the LSTM model relied on a single set of population‐level weights that remained unchanged during prediction. Consequently, the improved performance of AR(1) with longer follow‐up likely reflects the advantages of personalized model fitting rather than an inherent superiority of autoregressive modeling over deep learning.

Although the predictive performance of the AR(1) model improved as the number of examinations increased, the number of examinations was still small, with the longest follow‐up in the present study comprising only 11 examinations over a 5‐year period. Hence, the superior performance of the AR(1) model should be interpreted in the context of short‐term prediction. This observation is in agreement with previous studies indicating that AR(1) models are particularly suitable for short‐term forecasting [44, 45].

More recently, Abbasi et al. [21] compared a Hybrid‐VF‐Net architecture integrating convolutional neural networks (CNNs) and RNNs with standalone CNN and RNN models for VF prediction without incorporating reliability indices. The hybrid architecture had the lowest prediction error and outperformed both individual models. Furthermore, prediction accuracy declined for less reliable VF examinations, emphasizing the importance of reliability indices in VF prediction. These findings suggest that including complementary clinical information beyond VF measurements alone may further enhance predictive performance.

Finally, the present study has several limitations. First, although the proposed simulation framework accounted for between‐eye heterogeneity, within‐eye dependence, clinically defined progression scenarios, and realistic measurement variability, its reliance on a linear mixed‐effects model resulted in predominantly linear progression trajectories. Consequently, the simulated datasets may not fully capture the nonlinear progression patterns observed in clinical glaucoma populations. Nevertheless, by incorporating random effects, the framework accounted for heterogeneity in disease progression trajectories and BL VFs across eyes, generating simulated data that more closely reflected the complexity and variability of real‐world clinical data than those generated using simple linear regression [18]. Second, because the simulated datasets were generated from an inherently linear process, the better performance of the AR(1) model compared with the LSTM may have been partly attributable to a bias in favor of linear models. Third, although simulated data have the advantage of controlling progression rates, measurement variability, and follow‐up schedules while facilitating standardized model comparisons, they rely on predefined parametric assumptions and thus cannot fully reproduce the complex variability, noise characteristics, and irregular missing patterns observed in real‐world datasets. Fourth, the LSTM input representation in this study treats VF test locations as flat feature vectors, effectively ignoring the anatomically structured spatial relationships between them. While spatiotemporal architectures [21] could potentially capture these dependencies to improve accuracy, our primary objective was focusing on the temporal dynamics of progression using a standard LSTM model. Fifth, reliability indices—including false‐positive rates, false‐negative rates, and fixation losses—were not incorporated because these variables could not be realistically simulated within the current framework. Sixth, the autoregressive coefficient was estimated from relatively short longitudinal series and may, therefore, be subject to Hurwicz small‐sample bias, which generally leads to underestimation of the autoregressive coefficient. However, a previous simulation study [46] suggests that this estimation bias has a relatively little effect on point forecasts although it may have greater implications for prediction‐interval coverage and multistep forecasting. Given that the AR(1) models in the present study were primarily used for one‐step‐ahead point prediction, the practical impact of this bias on predictive accuracy is likely to be limited.

Accordingly, future research should evaluate the performance of these prediction models using real world clinical VF data as a complementary approach to the present simulation study. Using anatomically informed spatiotemporal architectures may further improve predictive accuracy by modeling spatial and temporal dependencies. In addition, future studies should integrate reliability indices together with VF measurements into deep learning models to enable a more clinically representative comparison with personalized statistical approaches. Because the naive BL forecast used in the present study relies solely on the most recent measurement, it may be affected by variability in a single VF measurement. A simple running‐average forecast could therefore be considered as an additional BL in future studies, as it may reduce the influence of variability in a single recent measurement and provide a reference for assessing the robustness of prediction model comparisons. Finally, simulating data with irregular follow‐up intervals and applying the IAR(1) model, which is more appropriate for irregularly spaced longitudinal data, could be a valuable direction for future research.

## 5. Conclusion

This study developed a simulation framework for comparing pointwise VF prediction models over time and showed that predictive performance depended on both the modeling approach and the amount of longitudinal data available. The population‐level LSTM achieved the highest accuracy with limited number of examinations, whereas the personalized AR(1) model became more accurate as additional examinations were conducted. Given that the length of the VF series was still relatively short as the number of examinations increased, these findings support the suitability of AR(1) for short‐term prediction. Overall, when a sufficient number of examinations is available, the results of the present study emphasize the value of personalized temporal modeling and highlight the potential importance of accounting for individual progression trajectories in longitudinal VF prediction.

## Author Contributions

S.N.: data generation, data analysis, and writing of the draft. E.M.: programming advisor. M.M.: clinical advisor and draft approver. M.D.: statistical advisor and drafted the manuscript. S.P.: research idea, study design, and improvement of the manuscript.

## Funding

This study was supported by the Research Council of Shiraz University of Medical Sciences, 29800.

## Disclosure

This paper was extracted from Soheila Naderi′s Ph.D. thesis under the supervision of Prof. Saeedeh Pourahmad.

## Conflicts of Interest

The authors declare no conflicts of interest.

## Supporting Information

Additional supporting information can be found online in the Supporting Information section.

## Supporting information


**Supporting Information 1** The codes for generating the simulated data are provided in File S1.

**Supporting Information 2** The codes used for data analysis are provided in File S2.

**Supporting Information 3**.

## Data Availability

The data that support the findings of this study are available from the corresponding author upon reasonable request.
