# Supplementary material for: Longitudinal Pointwise Visual Field Prediction in Early‐Stage Glaucoma Using Regression Models and Long Short‐Term Memory: A Simulation Study
Source: Biomed Res Int. 2026 Oct 21;2026:2815308. doi: 10.1155/bmri/2815308 (PMC13685520; doi:10.1155/bmri/2815308)
Supplement: Supplementary file 1 [file BMRI-2026-2815308-s001.docx]

Supplement Material- OLR Model Code

S2. OLR Model Implementation for Examination 6 Under the Slow Progression Rate Scenario

rm(list=ls())

gc()

library(dplyr)

library(caret)

library(readr)

library(openxlsx)

set.seed(123)

data <- read_csv("D:/felash10/USB Drive/phd/payanname phd/thesis/final.simulation/TD/models in slow progression_td/data_slow_ecc.csv")

norm_params <- list()

for (col in grep("^TD_", names(data), value = TRUE)) {

min_val <- min(data[[col]], na.rm = TRUE)

max_val <- max(data[[col]], na.rm = TRUE)

data[[paste0(col, "_norm")]] <- (data[[col]] - min_val) / (max_val - min_val)

norm_params[[col]] <- list(min = min_val, max = max_val)

}

denormalize <- function(x_norm, col_name) {

params <- norm_params[[gsub("_norm$", "", col_name)]]

return(x_norm * (params$max - params$min) + params$min)

{

data$eye <- rep(1:12000, each = 11)

set.seed(123)

test_eyes <- sample(1:12000, size = 0.15 * 12000)

data_test <- data %>% filter(eye %in% test_eyes)

data_rmse <- data.frame(eye = integer(), rmse = numeric())

data_mae <- data.frame(location = 1:52, mae = numeric(52))

mae_matrix <- matrix(0, nrow = length(test_eyes), ncol = 52)

for (i in seq_along(test_eyes)) {

eye_id <- test_eyes[i]

eye_data <- data_test %>% filter(eye == eye_id)

predictions <- c()

true_values <- c()

for (j in 1:52) {

td_col <- paste0("TD_", j, "_norm")

train_subset <- eye_data[1:5, ]

test_time <- eye_data$time[6]

true_value <- eye_data[[td_col]][6]

model <- lm(as.formula(paste(td_col, "~ time")), data = train_subset)

prediction <- predict(model, newdata = data.frame(time = test_time))

predictions <- c(predictions, prediction)

true_values <- c(true_values, true_value)

}

predictions_denorm <- sapply(1:52, function(j) denormalize(predictions[j], paste0("TD_", j)))

true_values_denorm <- sapply(1:52, function(j) denormalize(true_values[j], paste0("TD_", j)))

rmse_eye <- sqrt(mean((true_values_denorm - predictions_denorm)^2, na.rm = TRUE))

data_rmse <- rbind(data_rmse, data.frame(eye = eye_id, rmse = rmse_eye))

mae_matrix[i, ] <- abs(true_values_denorm - predictions_denorm)

{

data_mae$mae <- colMeans(mae_matrix, na.rm = TRUE)

write_csv(data_rmse, "D:/slow_rmse_OLR-5.csv")

write_csv(data_mae, "D:/slow_mae_OLR-5.csv")

mae_matrix_window10 <- mae_matrix

mae_per_eye_loc <- as.data.frame(mae_matrix_window10)

colnames(mae_per_eye_loc) <- paste0("TD_", 1:52)

write_csv(mae_per_eye_loc, "D:/slow_mae_per_eye_loc_OLR_5.csv")

Supplement Material- OLR Model Code

S2. OLR Model Implementation for Examination 7 Under the Slow Progression Rate Scenario

rm(list=ls())

gc()

library(dplyr)

library(caret)

library(readr)

library(openxlsx)

set.seed(123)

data <- read_csv("D:/felash10/USB Drive/phd/payanname phd/thesis/final.simulation/TD/models in slow progression_td/data_slow_ecc.csv")

norm_params <- list()

for (col in grep("^TD_", names(data), value = TRUE)) {

min_val <- min(data[[col]], na.rm = TRUE)

max_val <- max(data[[col]], na.rm = TRUE)

data[[paste0(col, "_norm")]] <- (data[[col]] - min_val) / (max_val - min_val)

norm_params[[col]] <- list(min = min_val, max = max_val)

{

denormalize <- function(x_norm, col_name) {

params <- norm_params[[gsub("_norm$", "", col_name)]]

return(x_norm * (params$max - params$min) + params$min)

{

data$eye <- rep(1:12000, each = 11)

set.seed(123)

test_eyes <- sample(1:12000, size = 0.15 * 12000)

data_test <- data %>% filter(eye %in% test_eyes)

data_rmse <- data.frame(eye = integer(), rmse = numeric())

data_mae <- data.frame(location = 1:52, mae = numeric(52))

mae_matrix <- matrix(0, nrow = length(test_eyes), ncol = 52)

for (i in seq_along(test_eyes)) {

eye_id <- test_eyes[i]

eye_data <- data_test %>% filter(eye == eye_id)

predictions <- c()

true_values <- c()

for (j in 1:52) {

td_col <- paste0("TD_", j, "_norm")

train_subset <- eye_data[1:6, ]

test_time <- eye_data$time[7]

true_value <- eye_data[[td_col]][7]

model <- lm(as.formula(paste(td_col, "~ time")), data = train_subset)

prediction <- predict(model, newdata = data.frame(time = test_time))

predictions <- c(predictions, prediction)

true_values <- c(true_values, true_value)

{

predictions_denorm <- sapply(1:52, function(j) denormalize(predictions[j], paste0("TD_", j)))

true_values_denorm <- sapply(1:52, function(j) denormalize(true_values[j], paste0("TD_", j)))

rmse_eye <- sqrt(mean((true_values_denorm - predictions_denorm)^2, na.rm = TRUE))

data_rmse <- rbind(data_rmse, data.frame(eye = eye_id, rmse = rmse_eye))

mae_matrix[i, ] <- abs(true_values_denorm - predictions_denorm)

{

data_mae$mae <- colMeans(mae_matrix, na.rm = TRUE)

write_csv(data_rmse, "D:/slow_rmse_OLR-6.csv")

write_csv(data_mae, "D:/slow_mae_OLR-6.csv")

mae_per_eye_loc <- as.data.frame(mae_matrix_window10)

colnames(mae_per_eye_loc) <- paste0("TD_", 1:52)

write_csv(mae_per_eye_loc, "D:/slow_mae_per_eye_loc_OLR_6.csv")

Supplement Material- OLR Model Code

S2. OLR Model Implementation for Examination 8 Under the Slow Progression Rate Scenario

rm(list=ls())

gc()

library(dplyr)

library(caret)

library(readr)

library(openxlsx)

set.seed(123)

data <- read_csv("D:/felash10/USB Drive/phd/payanname phd/thesis/final.simulation/TD/models in slow progression_td/data_slow_ecc.csv")

norm_params <- list()

for (col in grep("^TD_", names(data), value = TRUE)) {

min_val <- min(data[[col]], na.rm = TRUE)

max_val <- max(data[[col]], na.rm = TRUE)

data[[paste0(col, "_norm")]] <- (data[[col]] - min_val) / (max_val - min_val)

norm_params[[col]] <- list(min = min_val, max = max_val)

{

denormalize <- function(x_norm, col_name) {

params <- norm_params[[gsub("_norm$", "", col_name)]]

return(x_norm * (params$max - params$min) + params$min)

{

data$eye <- rep(1:12000, each = 11)

set.seed(123)

test_eyes <- sample(1:12000, size = 0.15 * 12000)

data_test <- data %>% filter(eye %in% test_eyes)

data_rmse <- data.frame(eye = integer(), rmse = numeric())

data_mae <- data.frame(location = 1:52, mae = numeric(52))

mae_matrix <- matrix(0, nrow = length(test_eyes), ncol = 52)

for (i in seq_along(test_eyes)) {

eye_id <- test_eyes[i]

eye_data <- data_test %>% filter(eye == eye_id)

predictions <- c()

true_values <- c()

for (j in 1:52) {

td_col <- paste0("TD_", j, "_norm")

train_subset <- eye_data[1:7, ]

test_time <- eye_data$time[8]

true_value <- eye_data[[td_col]][8]

model <- lm(as.formula(paste(td_col, "~ time")), data = train_subset)

prediction <- predict(model, newdata = data.frame(time = test_time))

predictions <- c(predictions, prediction)

true_values <- c(true_values, true_value)

{

predictions_denorm <- sapply(1:52, function(j) denormalize(predictions[j], paste0("TD_", j)))

true_values_denorm <- sapply(1:52, function(j) denormalize(true_values[j], paste0("TD_", j)))

rmse_eye <- sqrt(mean((true_values_denorm - predictions_denorm)^2, na.rm = TRUE))

data_rmse <- rbind(data_rmse, data.frame(eye = eye_id, rmse = rmse_eye))

mae_matrix[i, ] <- abs(true_values_denorm - predictions_denorm)

{

data_mae$mae <- colMeans(mae_matrix, na.rm = TRUE)

write_csv(data_rmse, "D:/slow_rmse_OLR-7.csv")

write_csv(data_mae, "D:/slow_mae_OLR-7.csv")

mae_per_eye_loc <- as.data.frame(mae_matrix_window10)

colnames(mae_per_eye_loc) <- paste0("TD_", 1:52)

write_csv(mae_per_eye_loc, "D:/slow_mae_per_eye_loc_OLR_7.csv")

Supplement Material- OLR Model Code

S2. OLR Model Implementation for Examination 9 Under the Slow Progression Rate Scenario

rm(list=ls())

gc()

library(dplyr)

library(caret)

library(readr)

library(openxlsx)

set.seed(123)

data <- read_csv("D:/felash10/USB Drive/phd/payanname phd/thesis/final.simulation/TD/models in slow progression_td/data_slow_ecc.csv")

norm_params <- list()

for (col in grep("^TD_", names(data), value = TRUE)) {

min_val <- min(data[[col]], na.rm = TRUE)

max_val <- max(data[[col]], na.rm = TRUE)

data[[paste0(col, "_norm")]] <- (data[[col]] - min_val) / (max_val - min_val)

norm_params[[col]] <- list(min = min_val, max = max_val)

}

denormalize <- function(x_norm, col_name) {

params <- norm_params[[gsub("_norm$", "", col_name)]]

return(x_norm * (params$max - params$min) + params$min)

}

data$eye <- rep(1:12000, each = 11)

set.seed(123)

test_eyes <- sample(1:12000, size = 0.15 * 12000)

data_test <- data %>% filter(eye %in% test_eyes)

data_rmse <- data.frame(eye = integer(), rmse = numeric())

data_mae <- data.frame(location = 1:52, mae = numeric(52))

mae_matrix <- matrix(0, nrow = length(test_eyes), ncol = 52)

for (i in seq_along(test_eyes)) {

eye_id <- test_eyes[i]

eye_data <- data_test %>% filter(eye == eye_id)

predictions <- c()

true_values <- c()

for (j in 1:52) {

td_col <- paste0("TD_", j, "_norm")

train_subset <- eye_data[1:8, ]

test_time <- eye_data$time[9]

true_value <- eye_data[[td_col]][9]

model <- lm(as.formula(paste(td_col, "~ time")), data = train_subset)

prediction <- predict(model, newdata = data.frame(time = test_time))

predictions <- c(predictions, prediction)

true_values <- c(true_values, true_value)

{

predictions_denorm <- sapply(1:52, function(j) denormalize(predictions[j], paste0("TD_", j)))

true_values_denorm <- sapply(1:52, function(j) denormalize(true_values[j], paste0("TD_", j)))

rmse_eye <- sqrt(mean((true_values_denorm - predictions_denorm)^2, na.rm = TRUE))

data_rmse <- rbind(data_rmse, data.frame(eye = eye_id, rmse = rmse_eye))

mae_matrix[i, ] <- abs(true_values_denorm - predictions_denorm)

{

data_mae$mae <- colMeans(mae_matrix, na.rm = TRUE)

write_csv(data_rmse, "D:/slow_rmse_OLR-8.csv")

write_csv(data_mae, "D:/slow_mae_OLR-8.csv")

mae_per_eye_loc <- as.data.frame(mae_matrix_window10)

colnames(mae_per_eye_loc) <- paste0("TD_", 1:52)

write_csv(mae_per_eye_loc, "D:/slow_mae_per_eye_loc_OLR_8.csv")

Supplement Material- OLR Model Code

S2. OLR Model Implementation for Examination 10 Under the Slow Progression Rate Scenario

rm(list=ls())

gc()

library(dplyr)

library(caret)

library(readr)

library(openxlsx)

set.seed(123)

data <- read_csv("D:/felash10/USB Drive/phd/payanname phd/thesis/final.simulation/TD/models in slow progression_td/data_slow_ecc.csv")

norm_params <- list()

for (col in grep("^TD_", names(data), value = TRUE)) {

min_val <- min(data[[col]], na.rm = TRUE)

max_val <- max(data[[col]], na.rm = TRUE)

data[[paste0(col, "_norm")]] <- (data[[col]] - min_val) / (max_val - min_val)

norm_params[[col]] <- list(min = min_val, max = max_val)

{

denormalize <- function(x_norm, col_name) {

params <- norm_params[[gsub("_norm$", "", col_name)]]

return(x_norm * (params$max - params$min) + params$min)

{

data$eye <- rep(1:12000, each = 11)

set.seed(123)

test_eyes <- sample(1:12000, size = 0.15 * 12000)

data_test <- data %>% filter(eye %in% test_eyes)

data_rmse <- data.frame(eye = integer(), rmse = numeric())

data_mae <- data.frame(location = 1:52, mae = numeric(52))

mae_matrix <- matrix(0, nrow = length(test_eyes), ncol = 52)

for (i in seq_along(test_eyes)) {

eye_id <- test_eyes[i]

eye_data <- data_test %>% filter(eye == eye_id)

predictions <- c()

true_values <- c()

for (j in 1:52) {

td_col <- paste0("TD_", j, "_norm")

train_subset <- eye_data[1:9, ]

test_time <- eye_data$time[10]

true_value <- eye_data[[td_col]][10]

model <- lm(as.formula(paste(td_col, "~ time")), data = train_subset)

prediction <- predict(model, newdata = data.frame(time = test_time))

predictions <- c(predictions, prediction)

true_values <- c(true_values, true_value)

{

predictions_denorm <- sapply(1:52, function(j) denormalize(predictions[j], paste0("TD_", j)))

true_values_denorm <- sapply(1:52, function(j) denormalize(true_values[j], paste0("TD_", j)))

rmse_eye <- sqrt(mean((true_values_denorm - predictions_denorm)^2, na.rm = TRUE))

data_rmse <- rbind(data_rmse, data.frame(eye = eye_id, rmse = rmse_eye))

mae_matrix[i, ] <- abs(true_values_denorm - predictions_denorm)

{

data_mae$mae <- colMeans(mae_matrix, na.rm = TRUE)

write_csv(data_rmse, "D:/slow_rmse_OLR-9.csv")

write_csv(data_mae, "D:/slow_mae_OLR-9.csv")

mae_per_eye_loc <- as.data.frame(mae_matrix_window10)

colnames(mae_per_eye_loc) <- paste0("TD_", 1:52)

write_csv(mae_per_eye_loc, "D:/slow_mae_per_eye_loc_OLR_9.csv")

Supplement Material- OLR Model Code

S2. OLR Model Implementation for Examination 11 Under the Slow Progression Rate Scenario

rm(list=ls())

gc()

library(dplyr)

library(caret)

library(readr)

library(openxlsx)

set.seed(123)

data <- read_csv("D:/felash10/USB Drive/phd/payanname phd/thesis/final.simulation/TD/models in slow progression_td/data_slow_ecc.csv")

norm_params <- list()

for (col in grep("^TD_", names(data), value = TRUE)) {

min_val <- min(data[[col]], na.rm = TRUE)

max_val <- max(data[[col]], na.rm = TRUE)

data[[paste0(col, "_norm")]] <- (data[[col]] - min_val) / (max_val - min_val)

norm_params[[col]] <- list(min = min_val, max = max_val)

}

denormalize <- function(x_norm, col_name) {

params <- norm_params[[gsub("_norm$", "", col_name)]]

return(x_norm * (params$max - params$min) + params$min)

}

data$eye <- rep(1:12000, each = 11)

set.seed(123)

test_eyes <- sample(1:12000, size = 0.15 * 12000)

data_test <- data %>% filter(eye %in% test_eyes)

data_rmse <- data.frame(eye = integer(), rmse = numeric())

data_mae <- data.frame(location = 1:52, mae = numeric(52))

mae_matrix <- matrix(0, nrow = length(test_eyes), ncol = 52)

for (i in seq_along(test_eyes)) {

eye_id <- test_eyes[i]

eye_data <- data_test %>% filter(eye == eye_id)

predictions <- c()

true_values <- c()

for (j in 1:52) {

td_col <- paste0("TD_", j, "_norm")

train_subset <- eye_data[1:10, ]

test_time <- eye_data$time[11]

true_value <- eye_data[[td_col]][11]

model <- lm(as.formula(paste(td_col, "~ time")), data = train_subset)

prediction <- predict(model, newdata = data.frame(time = test_time))

predictions <- c(predictions, prediction)

true_values <- c(true_values, true_value)

{

predictions_denorm <- sapply(1:52, function(j) denormalize(predictions[j], paste0("TD_", j)))

true_values_denorm <- sapply(1:52, function(j) denormalize(true_values[j], paste0("TD_", j)))

rmse_eye <- sqrt(mean((true_values_denorm - predictions_denorm)^2, na.rm = TRUE))

data_rmse <- rbind(data_rmse, data.frame(eye = eye_id, rmse = rmse_eye))

mae_matrix[i, ] <- abs(true_values_denorm - predictions_denorm)

{

data_mae$mae <- colMeans(mae_matrix, na.rm = TRUE)

write_csv(data_rmse, "D:/slow_rmse_OLR-10.csv")

write_csv(data_mae, "D:/slow_mae_OLR-10.csv")

mae_per_eye_loc <- as.data.frame(mae_matrix_window10)

colnames(mae_per_eye_loc) <- paste0("TD_", 1:52)

write_csv(mae_per_eye_loc, "D:/slow_mae_per_eye_loc_OLR_10.csv")

Supplement Material- OLR Model Code

S2. OLR Model Implementation for Examination 6 Under the Moderate Progression Rate Scenario

rm(list=ls())

gc()

library(dplyr)

library(caret)

library(readr)

library(openxlsx)

set.seed(123)

data <- read_csv("D:/felash10/USB Drive/phd/payanname phd/thesis/final.simulation/TD/models in average progression_td/data_average_ecc.csv")

norm_params <- list()

for (col in grep("^TD_", names(data), value = TRUE)) {

min_val <- min(data[[col]], na.rm = TRUE)

max_val <- max(data[[col]], na.rm = TRUE)

data[[paste0(col, "_norm")]] <- (data[[col]] - min_val) / (max_val - min_val)

norm_params[[col]] <- list(min = min_val, max = max_val)

{

denormalize <- function(x_norm, col_name) {

params <- norm_params[[gsub("_norm$", "", col_name)]]

return(x_norm * (params$max - params$min) + params$min)

}

data$eye <- rep(1:12000, each = 11)

set.seed(123)

test_eyes <- sample(1:12000, size = 0.15 * 12000)

data_test <- data %>% filter(eye %in% test_eyes)

data_rmse <- data.frame(eye = integer(), rmse = numeric())

data_mae <- data.frame(location = 1:52, mae = numeric(52))

mae_matrix <- matrix(0, nrow = length(test_eyes), ncol = 52)

for (i in seq_along(test_eyes)) {

eye_id <- test_eyes[i]

eye_data <- data_test %>% filter(eye == eye_id)

predictions <- c()

true_values <- c()

for (j in 1:52) {

td_col <- paste0("TD_", j, "_norm")

train_subset <- eye_data[1:5, ]

test_time <- eye_data$time[6]

true_value <- eye_data[[td_col]][6]

model <- lm(as.formula(paste(td_col, "~ time")), data = train_subset)

prediction <- predict(model, newdata = data.frame(time = test_time))

predictions <- c(predictions, prediction)

true_values <- c(true_values, true_value)

{

predictions_denorm <- sapply(1:52, function(j) denormalize(predictions[j], paste0("TD_", j)))

true_values_denorm <- sapply(1:52, function(j) denormalize(true_values[j], paste0("TD_", j)))

rmse_eye <- sqrt(mean((true_values_denorm - predictions_denorm)^2, na.rm = TRUE))

data_rmse <- rbind(data_rmse, data.frame(eye = eye_id, rmse = rmse_eye))

mae_matrix[i, ] <- abs(true_values_denorm - predictions_denorm)

{

data_mae$mae <- colMeans(mae_matrix, na.rm = TRUE)

write_csv(data_rmse, "D:/average_rmse_OLR-5.csv")

write_csv(data_mae, "D:/average_mae_OLR-5.csv")

mae_per_eye_loc <- as.data.frame(mae_matrix_window10)

colnames(mae_per_eye_loc) <- paste0("TD_", 1:52)

write_csv(mae_per_eye_loc, "D:/average_mae_per_eye_loc_OLR_5.csv")

Supplement Material- OLR Model Code

S2. OLR Model Implementation for Examination 7 Under the Moderate Progression Rate Scenario

rm(list=ls())

gc()

library(dplyr)

library(caret)

library(readr)

library(openxlsx)

set.seed(123)

data <- read_csv("D:/felash10/USB Drive/phd/payanname phd/thesis/final.simulation/TD/models in average progression_td/data_average_ecc.csv")

norm_params <- list()

for (col in grep("^TD_", names(data), value = TRUE)) {

min_val <- min(data[[col]], na.rm = TRUE)

max_val <- max(data[[col]], na.rm = TRUE)

data[[paste0(col, "_norm")]] <- (data[[col]] - min_val) / (max_val - min_val)

norm_params[[col]] <- list(min = min_val, max = max_val)

}

denormalize <- function(x_norm, col_name) {

params <- norm_params[[gsub("_norm$", "", col_name)]]

return(x_norm * (params$max - params$min) + params$min)

}

data$eye <- rep(1:12000, each = 11)

set.seed(123)

test_eyes <- sample(1:12000, size = 0.15 * 12000)

data_test <- data %>% filter(eye %in% test_eyes)

data_rmse <- data.frame(eye = integer(), rmse = numeric())

data_mae <- data.frame(location = 1:52, mae = numeric(52))

mae_matrix <- matrix(0, nrow = length(test_eyes), ncol = 52)

for (i in seq_along(test_eyes)) {

eye_id <- test_eyes[i]

eye_data <- data_test %>% filter(eye == eye_id)

predictions <- c()

true_values <- c()

for (j in 1:52) {

td_col <- paste0("TD_", j, "_norm")

train_subset <- eye_data[1:6, ]

test_time <- eye_data$time[7]

true_value <- eye_data[[td_col]][7]

model <- lm(as.formula(paste(td_col, "~ time")), data = train_subset)

prediction <- predict(model, newdata = data.frame(time = test_time))

predictions <- c(predictions, prediction)

true_values <- c(true_values, true_value)

}

predictions_denorm <- sapply(1:52, function(j) denormalize(predictions[j], paste0("TD_", j)))

true_values_denorm <- sapply(1:52, function(j) denormalize(true_values[j], paste0("TD_", j)))

rmse_eye <- sqrt(mean((true_values_denorm - predictions_denorm)^2, na.rm = TRUE))

data_rmse <- rbind(data_rmse, data.frame(eye = eye_id, rmse = rmse_eye))

mae_matrix[i, ] <- abs(true_values_denorm - predictions_denorm)

}

data_mae$mae <- colMeans(mae_matrix, na.rm = TRUE)

write_csv(data_rmse, "D:/average_rmse_OLR-6.csv")

write_csv(data_mae, "D:/average_mae_OLR-6.csv")

mae_per_eye_loc <- as.data.frame(mae_matrix_window10)

colnames(mae_per_eye_loc) <- paste0("TD_", 1:52)

print(head(mae_per_eye_loc[, 1:5]))

write_csv(mae_per_eye_loc, "D:/average_mae_per_eye_loc_OLR_6.csv")

Supplement Material- OLR Model Code

S2. OLR Model Implementation for Examination 8 Under the Moderate Progression Rate Scenario

rm(list=ls())

gc()

library(dplyr)

library(caret)

library(readr)

library(openxlsx)

set.seed(123)

data <- read_csv("D:/felash10/USB Drive/phd/payanname phd/thesis/final.simulation/TD/models in average progression_td/data_average_ecc.csv")

norm_params <- list()

for (col in grep("^TD_", names(data), value = TRUE)) {

min_val <- min(data[[col]], na.rm = TRUE)

max_val <- max(data[[col]], na.rm = TRUE)

data[[paste0(col, "_norm")]] <- (data[[col]] - min_val) / (max_val - min_val)

norm_params[[col]] <- list(min = min_val, max = max_val)

}

denormalize <- function(x_norm, col_name) {

params <- norm_params[[gsub("_norm$", "", col_name)]]

return(x_norm * (params$max - params$min) + params$min)

}

data$eye <- rep(1:12000, each = 11)

set.seed(123)

test_eyes <- sample(1:12000, size = 0.15 * 12000)

data_test <- data %>% filter(eye %in% test_eyes)

data_rmse <- data.frame(eye = integer(), rmse = numeric())

data_mae <- data.frame(location = 1:52, mae = numeric(52))

mae_matrix <- matrix(0, nrow = length(test_eyes), ncol = 52)

for (i in seq_along(test_eyes)) {

eye_id <- test_eyes[i]

eye_data <- data_test %>% filter(eye == eye_id)

predictions <- c()

true_values <- c()

for (j in 1:52) {

td_col <- paste0("TD_", j, "_norm")

train_subset <- eye_data[1:7, ]

test_time <- eye_data$time[8]

true_value <- eye_data[[td_col]][8]

model <- lm(as.formula(paste(td_col, "~ time")), data = train_subset)

prediction <- predict(model, newdata = data.frame(time = test_time))

predictions <- c(predictions, prediction)

true_values <- c(true_values, true_value)

}

predictions_denorm <- sapply(1:52, function(j) denormalize(predictions[j], paste0("TD_", j)))

true_values_denorm <- sapply(1:52, function(j) denormalize(true_values[j], paste0("TD_", j)))

rmse_eye <- sqrt(mean((true_values_denorm - predictions_denorm)^2, na.rm = TRUE))

data_rmse <- rbind(data_rmse, data.frame(eye = eye_id, rmse = rmse_eye))

mae_matrix[i, ] <- abs(true_values_denorm - predictions_denorm)

}

data_mae$mae <- colMeans(mae_matrix, na.rm = TRUE)

write_csv(data_rmse, "D:/average_rmse_OLR-7.csv")

write_csv(data_mae, "D:/average_mae_OLR-7.csv")

mae_per_eye_loc <- as.data.frame(mae_matrix_window10)

colnames(mae_per_eye_loc) <- paste0("TD_", 1:52)

write_csv(mae_per_eye_loc, "D:/average_mae_per_eye_loc_OLR_7.csv")

Supplement Material- OLR Model Code

S2. OLR Model Implementation for Examination 9 Under the Moderate Progression Rate Scenario

rm(list=ls())

gc()

library(dplyr)

library(caret)

library(readr)

library(openxlsx)

set.seed(123)

data <- read_csv("D:/felash10/USB Drive/phd/payanname phd/thesis/final.simulation/TD/models in average progression_td/data_average_ecc.csv")

norm_params <- list()

for (col in grep("^TD_", names(data), value = TRUE)) {

min_val <- min(data[[col]], na.rm = TRUE)

max_val <- max(data[[col]], na.rm = TRUE)

data[[paste0(col, "_norm")]] <- (data[[col]] - min_val) / (max_val - min_val)

norm_params[[col]] <- list(min = min_val, max = max_val)

{

denormalize <- function(x_norm, col_name) {

params <- norm_params[[gsub("_norm$", "", col_name)]]

return(x_norm * (params$max - params$min) + params$min)

{

data$eye <- rep(1:12000, each = 11)

set.seed(123)

test_eyes <- sample(1:12000, size = 0.15 * 12000)

data_test <- data %>% filter(eye %in% test_eyes)

data_rmse <- data.frame(eye = integer(), rmse = numeric())

data_mae <- data.frame(location = 1:52, mae = numeric(52))

mae_matrix <- matrix(0, nrow = length(test_eyes), ncol = 52)

for (i in seq_along(test_eyes)) {

eye_id <- test_eyes[i]

eye_data <- data_test %>% filter(eye == eye_id)

predictions <- c()

true_values <- c()

for (j in 1:52) {

td_col <- paste0("TD_", j, "_norm")

train_subset <- eye_data[1:8, ]

test_time <- eye_data$time[9]

true_value <- eye_data[[td_col]][9]

model <- lm(as.formula(paste(td_col, "~ time")), data = train_subset)

prediction <- predict(model, newdata = data.frame(time = test_time))

predictions <- c(predictions, prediction)

true_values <- c(true_values, true_value)

{

predictions_denorm <- sapply(1:52, function(j) denormalize(predictions[j], paste0("TD_", j)))

true_values_denorm <- sapply(1:52, function(j) denormalize(true_values[j], paste0("TD_", j)))

rmse_eye <- sqrt(mean((true_values_denorm - predictions_denorm)^2, na.rm = TRUE))

data_rmse <- rbind(data_rmse, data.frame(eye = eye_id, rmse = rmse_eye))

mae_matrix[i, ] <- abs(true_values_denorm - predictions_denorm)

{

data_mae$mae <- colMeans(mae_matrix, na.rm = TRUE)

write_csv(data_rmse, "D:/average_rmse_OLR-8.csv")

write_csv(data_mae, "D:/average_mae_OLR-8.csv")

mae_per_eye_loc <- as.data.frame(mae_matrix_window10)

colnames(mae_per_eye_loc) <- paste0("TD_", 1:52)

write_csv(mae_per_eye_loc, "D:/average_mae_per_eye_loc_OLR_8.csv")

Supplement Material- OLR Model Code

S2. OLR Model Implementation for Examination 10 Under the Moderate Progression Rate Scenario

rm(list=ls())

gc()

library(dplyr)

library(caret)

library(readr)

library(openxlsx)

set.seed(123)

data <- read_csv("D:/felash10/USB Drive/phd/payanname phd/thesis/final.simulation/TD/models in average progression_td/data_average_ecc.csv")

norm_params <- list()

for (col in grep("^TD_", names(data), value = TRUE)) {

min_val <- min(data[[col]], na.rm = TRUE)

max_val <- max(data[[col]], na.rm = TRUE)

data[[paste0(col, "_norm")]] <- (data[[col]] - min_val) / (max_val - min_val)

norm_params[[col]] <- list(min = min_val, max = max_val)

}

denormalize <- function(x_norm, col_name) {

params <- norm_params[[gsub("_norm$", "", col_name)]]

return(x_norm * (params$max - params$min) + params$min)

}

data$eye <- rep(1:12000, each = 11)

set.seed(123)

test_eyes <- sample(1:12000, size = 0.15 * 12000)

data_test <- data %>% filter(eye %in% test_eyes)

data_rmse <- data.frame(eye = integer(), rmse = numeric())

data_mae <- data.frame(location = 1:52, mae = numeric(52))

mae_matrix <- matrix(0, nrow = length(test_eyes), ncol = 52)

for (i in seq_along(test_eyes)) {

eye_id <- test_eyes[i]

eye_data <- data_test %>% filter(eye == eye_id)

predictions <- c()

true_values <- c()

for (j in 1:52) {

td_col <- paste0("TD_", j, "_norm")

train_subset <- eye_data[1:9, ]

test_time <- eye_data$time[10]

true_value <- eye_data[[td_col]][10]

model <- lm(as.formula(paste(td_col, "~ time")), data = train_subset)

prediction <- predict(model, newdata = data.frame(time = test_time))

predictions <- c(predictions, prediction)

true_values <- c(true_values, true_value)

}

predictions_denorm <- sapply(1:52, function(j) denormalize(predictions[j], paste0("TD_", j)))

true_values_denorm <- sapply(1:52, function(j) denormalize(true_values[j], paste0("TD_", j)))

rmse_eye <- sqrt(mean((true_values_denorm - predictions_denorm)^2, na.rm = TRUE))

data_rmse <- rbind(data_rmse, data.frame(eye = eye_id, rmse = rmse_eye))

mae_matrix[i, ] <- abs(true_values_denorm - predictions_denorm)

}

data_mae$mae <- colMeans(mae_matrix, na.rm = TRUE)

write_csv(data_rmse, "D:/average_rmse_OLR-9.csv")

write_csv(data_mae, "D:/average_mae_OLR-9.csv")

mae_per_eye_loc <- as.data.frame(mae_matrix_window10)

colnames(mae_per_eye_loc) <- paste0("TD_", 1:52)

write_csv(mae_per_eye_loc, "D:/average_mae_per_eye_loc_OLR_9.csv")

Supplement Material- OLR Model Code

S2. OLR Model Implementation for Examination 11 Under the Moderate Progression Rate Scenario

rm(list=ls())

gc()

library(dplyr)

library(caret)

library(readr)

library(openxlsx)

set.seed(123)

data <- read_csv("D:/felash10/USB Drive/phd/payanname phd/thesis/final.simulation/TD/models in average progression_td/data_average_ecc.csv")

norm_params <- list()

for (col in grep("^TD_", names(data), value = TRUE)) {

min_val <- min(data[[col]], na.rm = TRUE)

max_val <- max(data[[col]], na.rm = TRUE)

data[[paste0(col, "_norm")]] <- (data[[col]] - min_val) / (max_val - min_val)

norm_params[[col]] <- list(min = min_val, max = max_val)

}

denormalize <- function(x_norm, col_name) {

params <- norm_params[[gsub("_norm$", "", col_name)]]

return(x_norm * (params$max - params$min) + params$min)

}

data$eye <- rep(1:12000, each = 11)

set.seed(123)

test_eyes <- sample(1:12000, size = 0.15 * 12000)

data_test <- data %>% filter(eye %in% test_eyes)

data_rmse <- data.frame(eye = integer(), rmse = numeric())

data_mae <- data.frame(location = 1:52, mae = numeric(52))

mae_matrix <- matrix(0, nrow = length(test_eyes), ncol = 52)

for (i in seq_along(test_eyes)) {

eye_id <- test_eyes[i]

eye_data <- data_test %>% filter(eye == eye_id)

predictions <- c()

true_values <- c()

for (j in 1:52) {

td_col <- paste0("TD_", j, "_norm")

train_subset <- eye_data[1:10, ]

test_time <- eye_data$time[11]

true_value <- eye_data[[td_col]][11]

model <- lm(as.formula(paste(td_col, "~ time")), data = train_subset)

prediction <- predict(model, newdata = data.frame(time = test_time))

predictions <- c(predictions, prediction)

true_values <- c(true_values, true_value)

}

predictions_denorm <- sapply(1:52, function(j) denormalize(predictions[j], paste0("TD_", j)))

true_values_denorm <- sapply(1:52, function(j) denormalize(true_values[j], paste0("TD_", j)))

rmse_eye <- sqrt(mean((true_values_denorm - predictions_denorm)^2, na.rm = TRUE))

data_rmse <- rbind(data_rmse, data.frame(eye = eye_id, rmse = rmse_eye))

mae_matrix[i, ] <- abs(true_values_denorm - predictions_denorm)

}

data_mae$mae <- colMeans(mae_matrix, na.rm = TRUE)

write_csv(data_rmse, "D:/average_rmse_OLR-10.csv")

write_csv(data_mae, "D:/average_mae_OLR-10.csv")

mae_per_eye_loc <- as.data.frame(mae_matrix_window10)

colnames(mae_per_eye_loc) <- paste0("TD_", 1:52)

print(head(mae_per_eye_loc[, 1:5]))

write_csv(mae_per_eye_loc, "D:/average_mae_per_eye_loc_OLR_10.csv")

Supplement Material- OLR Model Code

S2. OLR Model Implementation for Examination 6 Under the Fast Progression Rate Scenario

rm(list=ls())

gc()

library(dplyr)

library(caret)

library(readr)

library(openxlsx)

set.seed(123)

data <- read_csv("D:/felash10/USB Drive/phd/payanname phd/thesis/final.simulation/TD/models in quick progression_td/data_quick_ecc.csv")

norm_params <- list()

for (col in grep("^TD_", names(data), value = TRUE)) {

min_val <- min(data[[col]], na.rm = TRUE)

max_val <- max(data[[col]], na.rm = TRUE)

data[[paste0(col, "_norm")]] <- (data[[col]] - min_val) / (max_val - min_val)

norm_params[[col]] <- list(min = min_val, max = max_val)

}

denormalize <- function(x_norm, col_name) {

params <- norm_params[[gsub("_norm$", "", col_name)]]

return(x_norm * (params$max - params$min) + params$min)

}

data$eye <- rep(1:12000, each = 11)

set.seed(123)

test_eyes <- sample(1:12000, size = 0.15 * 12000)

data_test <- data %>% filter(eye %in% test_eyes)

data_rmse <- data.frame(eye = integer(), rmse = numeric())

data_mae <- data.frame(location = 1:52, mae = numeric(52))

mae_matrix <- matrix(0, nrow = length(test_eyes), ncol = 52)

for (i in seq_along(test_eyes)) {

eye_id <- test_eyes[i]

eye_data <- data_test %>% filter(eye == eye_id)

predictions <- c()

true_values <- c()

for (j in 1:52) {

td_col <- paste0("TD_", j, "_norm")

train_subset <- eye_data[1:5, ]

test_time <- eye_data$time[6]

true_value <- eye_data[[td_col]][6]

model <- lm(as.formula(paste(td_col, "~ time")), data = train_subset)

prediction <- predict(model, newdata = data.frame(time = test_time))

predictions <- c(predictions, prediction)

true_values <- c(true_values, true_value)

}

predictions_denorm <- sapply(1:52, function(j) denormalize(predictions[j], paste0("TD_", j)))

true_values_denorm <- sapply(1:52, function(j) denormalize(true_values[j], paste0("TD_", j)))

rmse_eye <- sqrt(mean((true_values_denorm - predictions_denorm)^2, na.rm = TRUE))

data_rmse <- rbind(data_rmse, data.frame(eye = eye_id, rmse = rmse_eye))

mae_matrix[i, ] <- abs(true_values_denorm - predictions_denorm)

}

data_mae$mae <- colMeans(mae_matrix, na.rm = TRUE)

write_csv(data_rmse, "D:/quick_rmse_OLR-5.csv")

write_csv(data_mae, "D:/quick_mae_OLR-5.csv")

mae_per_eye_loc <- as.data.frame(mae_matrix_window10)

colnames(mae_per_eye_loc) <- paste0("TD_", 1:52)

print(head(mae_per_eye_loc[, 1:5]))

write_csv(mae_per_eye_loc, "D:/quick_mae_per_eye_loc_OLR_5.csv")

Supplement Material- OLR Model Code

S2. OLR Model Implementation for Examination 7 Under the Fast Progression Rate Scenario

rm(list=ls())

gc()

library(dplyr)

library(caret)

library(readr)

library(openxlsx)

set.seed(123)

data <- read_csv("D:/felash10/USB Drive/phd/payanname phd/thesis/final.simulation/TD/models in quick progression_td/data_quick_ecc.csv")

norm_params <- list()

for (col in grep("^TD_", names(data), value = TRUE)) {

min_val <- min(data[[col]], na.rm = TRUE)

max_val <- max(data[[col]], na.rm = TRUE)

data[[paste0(col, "_norm")]] <- (data[[col]] - min_val) / (max_val - min_val)

norm_params[[col]] <- list(min = min_val, max = max_val)

}

denormalize <- function(x_norm, col_name) {

params <- norm_params[[gsub("_norm$", "", col_name)]]

return(x_norm * (params$max - params$min) + params$min)

}

data$eye <- rep(1:12000, each = 11)

set.seed(123)

test_eyes <- sample(1:12000, size = 0.15 * 12000)

data_test <- data %>% filter(eye %in% test_eyes)

data_rmse <- data.frame(eye = integer(), rmse = numeric())

data_mae <- data.frame(location = 1:52, mae = numeric(52))

mae_matrix <- matrix(0, nrow = length(test_eyes), ncol = 52)

for (i in seq_along(test_eyes)) {

eye_id <- test_eyes[i]

eye_data <- data_test %>% filter(eye == eye_id)

predictions <- c()

true_values <- c()

for (j in 1:52) {

td_col <- paste0("TD_", j, "_norm")

train_subset <- eye_data[1:6, ]

test_time <- eye_data$time[7]

true_value <- eye_data[[td_col]][7]

model <- lm(as.formula(paste(td_col, "~ time")), data = train_subset)

prediction <- predict(model, newdata = data.frame(time = test_time))

predictions <- c(predictions, prediction)

true_values <- c(true_values, true_value)

}

predictions_denorm <- sapply(1:52, function(j) denormalize(predictions[j], paste0("TD_", j)))

true_values_denorm <- sapply(1:52, function(j) denormalize(true_values[j], paste0("TD_", j)))

rmse_eye <- sqrt(mean((true_values_denorm - predictions_denorm)^2, na.rm = TRUE))

data_rmse <- rbind(data_rmse, data.frame(eye = eye_id, rmse = rmse_eye))

mae_matrix[i, ] <- abs(true_values_denorm - predictions_denorm)

}

data_mae$mae <- colMeans(mae_matrix, na.rm = TRUE)

write_csv(data_rmse, "D:/quick_rmse_OLR-6.csv")

write_csv(data_mae, "D:/quick_mae_OLR-6.csv")

mae_matrix_window10 <- mae_matrix #

mae_per_eye_loc <- as.data.frame(mae_matrix_window10)

colnames(mae_per_eye_loc) <- paste0("TD_", 1:52)

write_csv(mae_per_eye_loc, "D:/quick_mae_per_eye_loc_OLR_6.csv")

Supplement Material- OLR Model Code

S2. OLR Model Implementation for Examination 8 Under the Fast Progression Rate Scenario

rm(list=ls())

gc()

library(dplyr)

library(caret)

library(readr)

library(openxlsx)

set.seed(123)

data <- read_csv("D:/felash10/USB Drive/phd/payanname phd/thesis/final.simulation/TD/models in quick progression_td/data_quick_ecc.csv")

norm_params <- list()

for (col in grep("^TD_", names(data), value = TRUE)) {

min_val <- min(data[[col]], na.rm = TRUE)

max_val <- max(data[[col]], na.rm = TRUE)

data[[paste0(col, "_norm")]] <- (data[[col]] - min_val) / (max_val - min_val)

norm_params[[col]] <- list(min = min_val, max = max_val)

{

denormalize <- function(x_norm, col_name) {

params <- norm_params[[gsub("_norm$", "", col_name)]]

return(x_norm * (params$max - params$min) + params$min)

{

data$eye <- rep(1:12000, each = 11)

set.seed(123)

test_eyes <- sample(1:12000, size = 0.15 * 12000)

data_test <- data %>% filter(eye %in% test_eyes)

data_rmse <- data.frame(eye = integer(), rmse = numeric())

data_mae <- data.frame(location = 1:52, mae = numeric(52))

mae_matrix <- matrix(0, nrow = length(test_eyes), ncol = 52)

for (i in seq_along(test_eyes)) {

eye_id <- test_eyes[i]

eye_data <- data_test %>% filter(eye == eye_id)

predictions <- c()

true_values <- c()

for (j in 1:52) {

td_col <- paste0("TD_", j, "_norm")

train_subset <- eye_data[1:7, ]

test_time <- eye_data$time[8]

true_value <- eye_data[[td_col]][8]

model <- lm(as.formula(paste(td_col, "~ time")), data = train_subset)

prediction <- predict(model, newdata = data.frame(time = test_time))

predictions <- c(predictions, prediction)

true_values <- c(true_values, true_value)

{

predictions_denorm <- sapply(1:52, function(j) denormalize(predictions[j], paste0("TD_", j)))

true_values_denorm <- sapply(1:52, function(j) denormalize(true_values[j], paste0("TD_", j)))

rmse_eye <- sqrt(mean((true_values_denorm - predictions_denorm)^2, na.rm = TRUE))

data_rmse <- rbind(data_rmse, data.frame(eye = eye_id, rmse = rmse_eye))

mae_matrix[i, ] <- abs(true_values_denorm - predictions_denorm)

{

data_mae$mae <- colMeans(mae_matrix, na.rm = TRUE)

write_csv(data_rmse, "D:/quick_rmse_OLR-7.csv")

write_csv(data_mae, "D:/quick_mae_OLR-7.csv")

mae_per_eye_loc <- as.data.frame(mae_matrix_window10)

colnames(mae_per_eye_loc) <- paste0("TD_", 1:52)

write_csv(mae_per_eye_loc, "D:/quick_mae_per_eye_loc_OLR_7.csv")

Supplement Material- OLR Model Code

S2. OLR Model Implementation for Examination 9 Under the Fast Progression Rate Scenario

rm(list=ls())

gc()

library(dplyr)

library(caret)

library(readr)

library(openxlsx)

set.seed(123)

data <- read_csv("D:/felash10/USB Drive/phd/payanname phd/thesis/final.simulation/TD/models in quick progression_td/data_quick_ecc.csv")

norm_params <- list()

for (col in grep("^TD_", names(data), value = TRUE)) {

min_val <- min(data[[col]], na.rm = TRUE)

max_val <- max(data[[col]], na.rm = TRUE)

data[[paste0(col, "_norm")]] <- (data[[col]] - min_val) / (max_val - min_val)

norm_params[[col]] <- list(min = min_val, max = max_val)

}

denormalize <- function(x_norm, col_name) {

params <- norm_params[[gsub("_norm$", "", col_name)]]

return(x_norm * (params$max - params$min) + params$min)

}

data$eye <- rep(1:12000, each = 11)

set.seed(123)

test_eyes <- sample(1:12000, size = 0.15 * 12000)

data_test <- data %>% filter(eye %in% test_eyes)

data_rmse <- data.frame(eye = integer(), rmse = numeric())

data_mae <- data.frame(location = 1:52, mae = numeric(52))

mae_matrix <- matrix(0, nrow = length(test_eyes), ncol = 52)

for (i in seq_along(test_eyes)) {

eye_id <- test_eyes[i]

eye_data <- data_test %>% filter(eye == eye_id)

predictions <- c()

true_values <- c()

for (j in 1:52) {

td_col <- paste0("TD_", j, "_norm")

train_subset <- eye_data[1:8, ]

test_time <- eye_data$time[9]

true_value <- eye_data[[td_col]][9]

model <- lm(as.formula(paste(td_col, "~ time")), data = train_subset)

prediction <- predict(model, newdata = data.frame(time = test_time))

predictions <- c(predictions, prediction)

true_values <- c(true_values, true_value)

}

predictions_denorm <- sapply(1:52, function(j) denormalize(predictions[j], paste0("TD_", j)))

true_values_denorm <- sapply(1:52, function(j) denormalize(true_values[j], paste0("TD_", j)))

rmse_eye <- sqrt(mean((true_values_denorm - predictions_denorm)^2, na.rm = TRUE))

data_rmse <- rbind(data_rmse, data.frame(eye = eye_id, rmse = rmse_eye))

mae_matrix[i, ] <- abs(true_values_denorm - predictions_denorm)

}

data_mae$mae <- colMeans(mae_matrix, na.rm = TRUE)

write_csv(data_rmse, "D:/quick_rmse_OLR-8.csv")

write_csv(data_mae, "D:/quick_mae_OLR-8.csv")

mae_per_eye_loc <- as.data.frame(mae_matrix_window10)

colnames(mae_per_eye_loc) <- paste0("TD_", 1:52)

write_csv(mae_per_eye_loc, "D:/quick_mae_per_eye_loc_OLR_8.csv")

Supplement Material- OLR Model Code

S2. OLR Model Implementation for Examination 10 Under the Fast Progression Rate Scenario

rm(list=ls())

gc()

library(dplyr)

library(caret)

library(readr)

library(openxlsx)

set.seed(123)

data <- read_csv("D:/felash10/USB Drive/phd/payanname phd/thesis/final.simulation/TD/models in quick progression_td/data_quick_ecc.csv")

norm_params <- list()

for (col in grep("^TD_", names(data), value = TRUE)) {

min_val <- min(data[[col]], na.rm = TRUE)

max_val <- max(data[[col]], na.rm = TRUE)

data[[paste0(col, "_norm")]] <- (data[[col]] - min_val) / (max_val - min_val)

norm_params[[col]] <- list(min = min_val, max = max_val)

}

denormalize <- function(x_norm, col_name) {

params <- norm_params[[gsub("_norm$", "", col_name)]]

return(x_norm * (params$max - params$min) + params$min)

}

data$eye <- rep(1:12000, each = 11)

set.seed(123)

test_eyes <- sample(1:12000, size = 0.15 * 12000)

data_test <- data %>% filter(eye %in% test_eyes)

data_rmse <- data.frame(eye = integer(), rmse = numeric())

data_mae <- data.frame(location = 1:52, mae = numeric(52))

mae_matrix <- matrix(0, nrow = length(test_eyes), ncol = 52)

for (i in seq_along(test_eyes)) {

eye_id <- test_eyes[i]

eye_data <- data_test %>% filter(eye == eye_id)

predictions <- c()

true_values <- c()

for (j in 1:52) {

td_col <- paste0("TD_", j, "_norm")

train_subset <- eye_data[1:9, ]

test_time <- eye_data$time[10]

true_value <- eye_data[[td_col]][10]

model <- lm(as.formula(paste(td_col, "~ time")), data = train_subset)

prediction <- predict(model, newdata = data.frame(time = test_time))

predictions <- c(predictions, prediction)

true_values <- c(true_values, true_value)

}

predictions_denorm <- sapply(1:52, function(j) denormalize(predictions[j], paste0("TD_", j)))

true_values_denorm <- sapply(1:52, function(j) denormalize(true_values[j], paste0("TD_", j)))

rmse_eye <- sqrt(mean((true_values_denorm - predictions_denorm)^2, na.rm = TRUE))

data_rmse <- rbind(data_rmse, data.frame(eye = eye_id, rmse = rmse_eye))

mae_matrix[i, ] <- abs(true_values_denorm - predictions_denorm)

}

data_mae$mae <- colMeans(mae_matrix, na.rm = TRUE)

write_csv(data_rmse, "D:/quick_rmse_OLR-9.csv")

write_csv(data_mae, "D:/quick_mae_OLR-9.csv")

mae_per_eye_loc <- as.data.frame(mae_matrix_window10)

colnames(mae_per_eye_loc) <- paste0("TD_", 1:52)

print(head(mae_per_eye_loc[, 1:5]))

write_csv(mae_per_eye_loc, "D:/quick_mae_per_eye_loc_OLR_9.csv")

Supplement Material- OLR Model Code

S2. OLR Model Implementation for Examination 11 Under the Fast Progression Rate Scenario

rm(list=ls())

gc()

library(dplyr)

library(caret)

library(readr)

library(openxlsx)

set.seed(123)

data <- read_csv("D:/felash10/USB Drive/phd/payanname phd/thesis/final.simulation/TD/models in quick progression_td/data_quick_ecc.csv")

norm_params <- list()

for (col in grep("^TD_", names(data), value = TRUE)) {

min_val <- min(data[[col]], na.rm = TRUE)

max_val <- max(data[[col]], na.rm = TRUE)

data[[paste0(col, "_norm")]] <- (data[[col]] - min_val) / (max_val - min_val)

norm_params[[col]] <- list(min = min_val, max = max_val)

}

denormalize <- function(x_norm, col_name) {

params <- norm_params[[gsub("_norm$", "", col_name)]]

return(x_norm * (params$max - params$min) + params$min)

}

data$eye <- rep(1:12000, each = 11)

set.seed(123)

test_eyes <- sample(1:12000, size = 0.15 * 12000)

data_test <- data %>% filter(eye %in% test_eyes)

data_rmse <- data.frame(eye = integer(), rmse = numeric())

data_mae <- data.frame(location = 1:52, mae = numeric(52))

mae_matrix <- matrix(0, nrow = length(test_eyes), ncol = 52)

for (i in seq_along(test_eyes)) {

eye_id <- test_eyes[i]

eye_data <- data_test %>% filter(eye == eye_id)

predictions <- c()

true_values <- c()

for (j in 1:52) {

td_col <- paste0("TD_", j, "_norm")

train_subset <- eye_data[1:10, ]

test_time <- eye_data$time[11]

true_value <- eye_data[[td_col]][11]

model <- lm(as.formula(paste(td_col, "~ time")), data = train_subset)

prediction <- predict(model, newdata = data.frame(time = test_time))

predictions <- c(predictions, prediction)

true_values <- c(true_values, true_value)

}

predictions_denorm <- sapply(1:52, function(j) denormalize(predictions[j], paste0("TD_", j)))

true_values_denorm <- sapply(1:52, function(j) denormalize(true_values[j], paste0("TD_", j)))

rmse_eye <- sqrt(mean((true_values_denorm - predictions_denorm)^2, na.rm = TRUE))

data_rmse <- rbind(data_rmse, data.frame(eye = eye_id, rmse = rmse_eye))

mae_matrix[i, ] <- abs(true_values_denorm - predictions_denorm)

}

data_mae$mae <- colMeans(mae_matrix, na.rm = TRUE)

write_csv(data_rmse, "D:/quick_rmse_OLR-10.csv")

write_csv(data_mae, "D:/quick_mae_OLR-10.csv")

mae_per_eye_loc <- as.data.frame(mae_matrix_window10)

colnames(mae_per_eye_loc) <- paste0("TD_", 1:52)

write_csv(mae_per_eye_loc, "D:/quick_mae_per_eye_loc_OLR_10.csv")

Supplement Material- AR(1) Model Code

S2. AR(1) Model Implementation for Examination 6 Under the Slow Progression Rate Scenario

rm(list = ls())

gc()

library(dplyr)

library(caret)

library(readr)

library(TSA)

library(openxlsx)

library(stats)

library(forecast)

set.seed(123)

data <- read_csv("D:/felash10/USB Drive/phd/payanname phd/thesis/final.simulation/TD/models in slow progression_td/data_slow_ecc.csv")

norm_params <- list()

for (col in grep("^TD_", names(data), value = TRUE)) {

min_val <- min(data[[col]], na.rm = TRUE)

max_val <- max(data[[col]], na.rm = TRUE)

data[[paste0(col, "_norm")]] <- (data[[col]] - min_val) / (max_val - min_val)

norm_params[[col]] <- list(min = min_val, max = max_val)

{

denormalize <- function(x_norm, col_name) {

params <- norm_params[[gsub("_norm$", "", col_name)]]

return(x_norm * (params$max - params$min) + params$min)

{

data$eye <- rep(1:12000, each = 11)

set.seed(123)

test_eyes <- sample(1:12000, size = 0.15 * 12000)

data_test <- data %>% filter(eye %in% test_eyes)

data_rmse <- data.frame(eye = integer(), rmse = numeric())

data_mae <- data.frame(location = 1:52, mae = numeric(52))

mae_matrix <- matrix(0, nrow = length(test_eyes), ncol = 52)

for (i in seq_along(test_eyes)) {

eye_id <- test_eyes[i]

eye_data <- data_test %>% filter(eye == eye_id)

eye_data_subset <- eye_data[1:6, ]

predictions <- numeric(52)

true_values <- numeric(52)

for (j in 1:52) {

td_col <- paste0("TD_", j, "_norm")

series <- eye_data_subset[[td_col]]

time <- eye_data_subset$time

lm_model <- lm(series ~ time)

p_val <- summary(lm_model)$coefficients[2, 4]

if (!is.na(p_val) && p_val < 0.05) {

detrended <- residuals(lm_model)

trend_at_6 <- predict(lm_model, newdata = data.frame(time = time[6]))

else {{

detrended <- series

trend_at_6 <- 0

{

train_subset <- ts(detrended[1:5], start = min(time), frequency = 1)

true_values[j] <- series[6]

tryCatch({

ar_model <- Arima(train_subset, order = c(1, 0, 0), method = "ML")

prediction_resid <- forecast(ar_model, h = 1)$mean

predictions[j] <- prediction_resid + trend_at_6

, error = function(e) {{

predictions[j] <- NA

({

{

predictions_denorm <- sapply(1:52, function(j) denormalize(predictions[j], paste0("TD_", j)))

true_values_denorm <- sapply(1:52, function(j) denormalize(true_values[j], paste0("TD_", j)))

rmse_eye <- sqrt(mean((true_values_denorm - predictions_denorm)^2, na.rm = TRUE))

data_rmse <- rbind(data_rmse, data.frame(eye = eye_id, rmse = rmse_eye))

mae_matrix[i, ] <- abs(true_values_denorm - predictions_denorm)

{

data_mae$mae <- colMeans(mae_matrix, na.rm = TRUE)

write_csv(data_rmse, "D:/slow_rmse_AR1-5.csv")

write_csv(data_mae, "D:/slow_mae_AR1-5.csv")

mae_per_eye_loc <- as.data.frame(mae_matrix_window10)

colnames(mae_per_eye_loc) <- paste0("TD_", 1:52)

print(head(mae_per_eye_loc[, 1:5]))

write_csv(mae_per_eye_loc, "D:/slow_mae_per_eye_loc_ar1_5.csv")

Supplement Material- AR(1) Model Code

S2. AR(1) Model Implementation for Examination 7 Under the Slow Progression Rate Scenario

rm(list = ls())

gc()

library(dplyr)

library(caret)

library(readr)

library(TSA)

library(openxlsx)

library(stats)

library(forecast)

set.seed(123)

data <- read_csv("D:/felash10/USB Drive/phd/payanname phd/thesis/final.simulation/TD/models in slow progression_td/data_slow_ecc.csv")

norm_params <- list()

for (col in grep("^TD_", names(data), value = TRUE)) {

min_val <- min(data[[col]], na.rm = TRUE)

max_val <- max(data[[col]], na.rm = TRUE)

data[[paste0(col, "_norm")]] <- (data[[col]] - min_val) / (max_val - min_val)

norm_params[[col]] <- list(min = min_val, max = max_val)

}

denormalize <- function(x_norm, col_name) {

params <- norm_params[[gsub("_norm$", "", col_name)]]

return(x_norm * (params$max - params$min) + params$min)

}

data$eye <- rep(1:12000, each = 11)

set.seed(123)

test_eyes <- sample(1:12000, size = 0.15 * 12000)

data_test <- data %>% filter(eye %in% test_eyes)

data_rmse <- data.frame(eye = integer(), rmse = numeric())

data_mae <- data.frame(location = 1:52, mae = numeric(52))

mae_matrix <- matrix(0, nrow = length(test_eyes), ncol = 52)

for (i in seq_along(test_eyes)) {

eye_id <- test_eyes[i]

eye_data <- data_test %>% filter(eye == eye_id)

eye_data_subset <- eye_data[1:7, ]

predictions <- numeric(52)

true_values <- numeric(52)

for (j in 1:52) {

td_col <- paste0("TD_", j, "_norm")

series <- eye_data_subset[[td_col]]

time <- eye_data_subset$time

lm_model <- lm(series ~ time)

p_val <- summary(lm_model)$coefficients[2, 4]

if (!is.na(p_val) && p_val < 0.05) {

detrended <- residuals(lm_model)

trend_at_6 <- predict(lm_model, newdata = data.frame(time = time[7]))

}else {

detrended <- series

trend_at_6 <- 0

}

train_subset <- ts(detrended[1:6], start = min(time), frequency = 1)

true_values[j] <- series[7]

tryCatch({

ar_model <- Arima(train_subset, order = c(1, 0, 0), method = "ML")

prediction_resid <- forecast(ar_model, h = 1)$mean

predictions[j] <- prediction_resid + trend_at_6

} , error = function(e) {

}

predictions[j] <- NA

})

}

predictions_denorm <- sapply(1:52, function(j) denormalize(predictions[j], paste0("TD_", j)))

true_values_denorm <- sapply(1:52, function(j) denormalize(true_values[j], paste0("TD_", j)))

rmse_eye <- sqrt(mean((true_values_denorm - predictions_denorm)^2, na.rm = TRUE))

data_rmse <- rbind(data_rmse, data.frame(eye = eye_id, rmse = rmse_eye))

mae_matrix[i, ] <- abs(true_values_denorm - predictions_denorm)

}

data_mae$mae <- colMeans(mae_matrix, na.rm = TRUE)

write_csv(data_rmse, "D:/slow_rmse_AR1-6.csv")

write_csv(data_mae, "D:/slow_mae_AR1-6.csv")

mae_per_eye_loc <- as.data.frame(mae_matrix_window10)

colnames(mae_per_eye_loc) <- paste0("TD_", 1:52)

write_csv(mae_per_eye_loc, "D:/slow_mae_per_eye_loc_ar1_6.csv")

Supplement Material- AR(1) Model Code

S2. AR(1) Model Implementation for Examination 8 Under the Slow Progression Rate Scenario

rm(list = ls())

gc()

library(dplyr)

library(caret)

library(readr)

library(TSA)

library(openxlsx)

library(stats)

library(forecast)

set.seed(123)

data <- read_csv("D:/felash10/USB Drive/phd/payanname phd/thesis/final.simulation/TD/models in slow progression_td/data_slow_ecc.csv")

norm_params <- list()

for (col in grep("^TD_", names(data), value = TRUE)) {

min_val <- min(data[[col]], na.rm = TRUE)

max_val <- max(data[[col]], na.rm = TRUE)

data[[paste0(col, "_norm")]] <- (data[[col]] - min_val) / (max_val - min_val)

norm_params[[col]] <- list(min = min_val, max = max_val)

}

denormalize <- function(x_norm, col_name) {

params <- norm_params[[gsub("_norm$", "", col_name)]]

return(x_norm * (params$max - params$min) + params$min)

{

data$eye <- rep(1:12000, each = 11)

set.seed(123)

test_eyes <- sample(1:12000, size = 0.15 * 12000)

data_test <- data %>% filter(eye %in% test_eyes)

data_rmse <- data.frame(eye = integer(), rmse = numeric())

data_mae <- data.frame(location = 1:52, mae = numeric(52))

mae_matrix <- matrix(0, nrow = length(test_eyes), ncol = 52)

for (i in seq_along(test_eyes)) {

eye_id <- test_eyes[i]

eye_data <- data_test %>% filter(eye == eye_id)

eye_data_subset <- eye_data[1:8, ]

predictions <- numeric(52)

true_values <- numeric(52)

for (j in 1:52) {

td_col <- paste0("TD_", j, "_norm")

series <- eye_data_subset[[td_col]]

time <- eye_data_subset$time

lm_model <- lm(series ~ time)

p_val <- summary(lm_model)$coefficients[2, 4]

if (!is.na(p_val) && p_val < 0.05) {

detrended <- residuals(lm_model)

trend_at_6 <- predict(lm_model, newdata = data.frame(time = time[8]))

}else {

detrended <- series

trend_at_6 <- 0

}

train_subset <- ts(detrended[1:7], start = min(time), frequency = 1)

true_values[j] <- series[8]

tryCatch({

ar_model <- Arima(train_subset, order = c(1, 0, 0), method = "ML")

prediction_resid <- forecast(ar_model, h = 1)$mean

predictions[j] <- prediction_resid + trend_at_6

},error = function(e) {

predictions[j] <- NA

({

{

predictions_denorm <- sapply(1:52, function(j) denormalize(predictions[j], paste0("TD_", j)))

true_values_denorm <- sapply(1:52, function(j) denormalize(true_values[j], paste0("TD_", j)))

rmse_eye <- sqrt(mean((true_values_denorm - predictions_denorm)^2, na.rm = TRUE))

data_rmse <- rbind(data_rmse, data.frame(eye = eye_id, rmse = rmse_eye))

mae_matrix[i, ] <- abs(true_values_denorm - predictions_denorm)

{

data_mae$mae <- colMeans(mae_matrix, na.rm = TRUE)

write_csv(data_rmse, "D:/slow_rmse_AR1-7.csv")

write_csv(data_mae, "D:/slow_mae_AR1-7.csv")

mae_per_eye_loc <- as.data.frame(mae_matrix_window10)

colnames(mae_per_eye_loc) <- paste0("TD_", 1:52)

write_csv(mae_per_eye_loc, "D:/slow_mae_per_eye_loc_ar1_7.csv")

Supplement Material- AR(1) Model Code

S2. AR(1) Model Implementation for Examination 9 Under the Slow Progression Rate Scenario

rm(list = ls())

gc()

library(dplyr)

library(caret)

library(readr)

library(TSA)

library(openxlsx)

library(stats)

library(forecast)

set.seed(123)

data <- read_csv("D:/felash10/USB Drive/phd/payanname phd/thesis/final.simulation/TD/models in slow progression_td/data_slow_ecc.csv")

norm_params <- list()

for (col in grep("^TD_", names(data), value = TRUE)) {

min_val <- min(data[[col]], na.rm = TRUE)

max_val <- max(data[[col]], na.rm = TRUE)

data[[paste0(col, "_norm")]] <- (data[[col]] - min_val) / (max_val - min_val)

norm_params[[col]] <- list(min = min_val, max = max_val)

}

denormalize <- function(x_norm, col_name) {

params <- norm_params[[gsub("_norm$", "", col_name)]]

return(x_norm * (params$max - params$min) + params$min)

}

data$eye <- rep(1:12000, each = 11)

set.seed(123)

test_eyes <- sample(1:12000, size = 0.15 * 12000)

data_test <- data %>% filter(eye %in% test_eyes)

data_rmse <- data.frame(eye = integer(), rmse = numeric())

data_mae <- data.frame(location = 1:52, mae = numeric(52))

mae_matrix <- matrix(0, nrow = length(test_eyes), ncol = 52)

for (i in seq_along(test_eyes)) {

eye_id <- test_eyes[i]

eye_data <- data_test %>% filter(eye == eye_id)

eye_data_subset <- eye_data[1:9, ]

predictions <- numeric(52)

true_values <- numeric(52)

for (j in 1:52) {

td_col <- paste0("TD_", j, "_norm")

series <- eye_data_subset[[td_col]]

time <- eye_data_subset$time

lm_model <- lm(series ~ time)

p_val <- summary(lm_model)$coefficients[2, 4]

if (!is.na(p_val) && p_val < 0.05) {

detrended <- residuals(lm_model)

trend_at_6 <- predict(lm_model, newdata = data.frame(time = time[9]))

}else {

detrended <- series

trend_at_6 <- 0

{

train_subset <- ts(detrended[1:8], start = min(time), frequency = 1)

true_values[j] <- series[9]

tryCatch({

ar_model <- Arima(train_subset, order = c(1, 0, 0), method = "ML")

prediction_resid <- forecast(ar_model, h = 1)$mean

predictions[j] <- prediction_resid + trend_at_6

} ,error = function(e){

predictions[j] <- NA

})

}

predictions_denorm <- sapply(1:52, function(j) denormalize(predictions[j], paste0("TD_", j)))

true_values_denorm <- sapply(1:52, function(j) denormalize(true_values[j], paste0("TD_", j)))

rmse_eye <- sqrt(mean((true_values_denorm - predictions_denorm)^2, na.rm = TRUE))

data_rmse <- rbind(data_rmse, data.frame(eye = eye_id, rmse = rmse_eye))

mae_matrix[i, ] <- abs(true_values_denorm - predictions_denorm)

}

data_mae$mae <- colMeans(mae_matrix, na.rm = TRUE)

write_csv(data_rmse, "D:/slow_rmse_AR1-8.csv")

write_csv(data_mae, "D:/slow_mae_AR1-8.csv")

mae_per_eye_loc <- as.data.frame(mae_matrix_window10)

colnames(mae_per_eye_loc) <- paste0("TD_", 1:52)

write_csv(mae_per_eye_loc, "D:/slow_mae_per_eye_loc_ar1_8.csv")

Supplement Material- AR(1) Model Code

S2. AR(1) Model Implementation for Examination 10 Under the Slow Progression Rate Scenario

rm(list = ls())

gc()

library(dplyr)

library(caret)

library(readr)

library(TSA)

library(openxlsx)

library(stats)

library(forecast)

set.seed(123)

data <- read_csv("D:/felash10/USB Drive/phd/payanname phd/thesis/final.simulation/TD/models in slow progression_td/data_slow_ecc.csv")

norm_params <- list()

for (col in grep("^TD_", names(data), value = TRUE)) {

min_val <- min(data[[col]], na.rm = TRUE)

max_val <- max(data[[col]], na.rm = TRUE)

data[[paste0(col, "_norm")]] <- (data[[col]] - min_val) / (max_val - min_val)

norm_params[[col]] <- list(min = min_val, max = max_val)

{

denormalize <- function(x_norm, col_name) {

params <- norm_params[[gsub("_norm$", "", col_name)]]

return(x_norm * (params$max - params$min) + params$min)

}

data$eye <- rep(1:12000, each = 11)

set.seed(123)

test_eyes <- sample(1:12000, size = 0.15 * 12000)

data_test <- data %>% filter(eye %in% test_eyes)

data_rmse <- data.frame(eye = integer(), rmse = numeric())

data_mae <- data.frame(location = 1:52, mae = numeric(52))

mae_matrix <- matrix(0, nrow = length(test_eyes), ncol = 52)

for (i in seq_along(test_eyes)) {

eye_id <- test_eyes[i]

eye_data <- data_test %>% filter(eye == eye_id)

eye_data_subset <- eye_data[1:10, ]

predictions <- numeric(52)

true_values <- numeric(52)

for (j in 1:52) {

td_col <- paste0("TD_", j, "_norm")

series <- eye_data_subset[[td_col]]

time <- eye_data_subset$time

lm_model <- lm(series ~ time)

p_val <- summary(lm_model)$coefficients[2, 4]

if (!is.na(p_val) && p_val < 0.05) {

detrended <- residuals(lm_model)

trend_at_6 <- predict(lm_model, newdata = data.frame(time = time[10]))

}else {

detrended <- series

trend_at_6 <- 0

{

train_subset <- ts(detrended[1:9], start = min(time), frequency = 1)

true_values[j] <- series[10]

tryCatch({

ar_model <- Arima(train_subset, order = c(1, 0, 0), method = "ML")

prediction_resid <- forecast(ar_model, h = 1)$mean

predictions[j] <- prediction_resid + trend_at_6

,error = function(e) {{

predictions[j] <- NA

})

}

predictions_denorm <- sapply(1:52, function(j) denormalize(predictions[j], paste0("TD_", j)))

true_values_denorm <- sapply(1:52, function(j) denormalize(true_values[j], paste0("TD_", j)))

rmse_eye <- sqrt(mean((true_values_denorm - predictions_denorm)^2, na.rm = TRUE))

data_rmse <- rbind(data_rmse, data.frame(eye = eye_id, rmse = rmse_eye))

mae_matrix[i, ] <- abs(true_values_denorm - predictions_denorm)

{

data_mae$mae <- colMeans(mae_matrix, na.rm = TRUE)

write_csv(data_rmse, "D:/slow_rmse_AR1-9.csv")

write_csv(data_mae, "D:/slow_mae_AR1-9.csv")

mae_per_eye_loc <- as.data.frame(mae_matrix_window10)

colnames(mae_per_eye_loc) <- paste0("TD_", 1:52)

write_csv(mae_per_eye_loc, "D:/slow_mae_per_eye_loc_ar1_9.csv")

Supplement Material- AR(1) Model Code

S2. AR(1) Model Implementation for Examination 11 Under the Slow Progression Rate Scenario

rm(list = ls())

gc()

library(dplyr)

library(caret)

library(readr)

library(TSA)

library(openxlsx)

library(stats)

library(forecast)

set.seed(123)

data <- read_csv("D:/felash10/USB Drive/phd/payanname phd/thesis/final.simulation/TD/models in slow progression_td/data_slow_ecc.csv")

norm_params <- list()

for (col in grep("^TD_", names(data), value = TRUE)) {

min_val <- min(data[[col]], na.rm = TRUE)

max_val <- max(data[[col]], na.rm = TRUE)

data[[paste0(col, "_norm")]] <- (data[[col]] - min_val) / (max_val - min_val)

norm_params[[col]] <- list(min = min_val, max = max_val)

}

denormalize <- function(x_norm, col_name) {

params <- norm_params[[gsub("_norm$", "", col_name)]]

return(x_norm * (params$max - params$min) + params$min)

}

data$eye <- rep(1:12000, each = 11)

set.seed(123)

test_eyes <- sample(1:12000, size = 0.15 * 12000)

data_test <- data %>% filter(eye %in% test_eyes)

data_rmse <- data.frame(eye = integer(), rmse = numeric())

data_mae <- data.frame(location = 1:52, mae = numeric(52))

mae_matrix <- matrix(0, nrow = length(test_eyes), ncol = 52)

for (i in seq_along(test_eyes)) {

eye_id <- test_eyes[i]

eye_data <- data_test %>% filter(eye == eye_id)

eye_data_subset <- eye_data[1:11, ]

predictions <- numeric(52)

true_values <- numeric(52)

for (j in 1:52) {

td_col <- paste0("TD_", j, "_norm")

series <- eye_data_subset[[td_col]]

time <- eye_data_subset$time

lm_model <- lm(series ~ time)

p_val <- summary(lm_model)$coefficients[2, 4]

if (!is.na(p_val) && p_val < 0.05) {

detrended <- residuals(lm_model)

trend_at_6 <- predict(lm_model, newdata = data.frame(time = time[11]))

else {{

detrended <- series

trend_at_6 <- 0

{

train_subset <- ts(detrended[1:10], start = min(time), frequency = 1)

true_values[j] <- series[11]

tryCatch({

ar_model <- Arima(train_subset, order = c(1, 0, 0), method = "ML")

prediction_resid <- forecast(ar_model, h = 1)$mean

predictions[j] <- prediction_resid + trend_at_6

},error = function(e){

predictions[j] <- NA

})

}

predictions_denorm <- sapply(1:52, function(j) denormalize(predictions[j], paste0("TD_", j)))

true_values_denorm <- sapply(1:52, function(j) denormalize(true_values[j], paste0("TD_", j)))

rmse_eye <- sqrt(mean((true_values_denorm - predictions_denorm)^2, na.rm = TRUE))

data_rmse <- rbind(data_rmse, data.frame(eye = eye_id, rmse = rmse_eye))

mae_matrix[i, ] <- abs(true_values_denorm - predictions_denorm)

}

data_mae$mae <- colMeans(mae_matrix, na.rm = TRUE)

write_csv(data_rmse, "D:/slow_rmse_AR1-10.csv")

write_csv(data_mae, "D:/slow_mae_AR1-10.csv")

mae_per_eye_loc <- as.data.frame(mae_matrix_window10)

colnames(mae_per_eye_loc) <- paste0("TD_", 1:52)

write_csv(mae_per_eye_loc, "D:/slow_mae_per_eye_loc_ar1_10.csv")

Supplement Material- AR(1) Model Code

S2. AR(1) Model Implementation for Examination 6 Under the Moderate Progression Rate Scenario

rm(list = ls())

gc()

library(dplyr)

library(caret)

library(readr)

library(TSA)

library(openxlsx)

library(stats)

library(forecast)

set.seed(123)

data <- read_csv("D:/felash10/USB Drive/phd/payanname phd/thesis/final.simulation/TD/models in average progression_td/data_average_ecc.csv")

norm_params <- list()

for (col in grep("^TD_", names(data), value = TRUE)) {

min_val <- min(data[[col]], na.rm = TRUE)

max_val <- max(data[[col]], na.rm = TRUE)

data[[paste0(col, "_norm")]] <- (data[[col]] - min_val) / (max_val - min_val)

norm_params[[col]] <- list(min = min_val, max = max_val)

}

denormalize <- function(x_norm, col_name) {

params <- norm_params[[gsub("_norm$", "", col_name)]]

return(x_norm * (params$max - params$min) + params$min)

}

data$eye <- rep(1:12000, each = 11)

set.seed(123)

test_eyes <- sample(1:12000, size = 0.15 * 12000)

data_test <- data %>% filter(eye %in% test_eyes)

data_rmse <- data.frame(eye = integer(), rmse = numeric())

data_mae <- data.frame(location = 1:52, mae = numeric(52))

mae_matrix <- matrix(0, nrow = length(test_eyes), ncol = 52)

for (i in seq_along(test_eyes)) {

eye_id <- test_eyes[i]

eye_data <- data_test %>% filter(eye == eye_id)

eye_data_subset <- eye_data[1:6, ]

predictions <- numeric(52)

true_values <- numeric(52)

for (j in 1:52) {

td_col <- paste0("TD_", j, "_norm")

series <- eye_data_subset[[td_col]]

time <- eye_data_subset$time

lm_model <- lm(series ~ time)

p_val <- summary(lm_model)$coefficients[2, 4]

if (!is.na(p_val) && p_val < 0.05) {

detrended <- residuals(lm_model)

trend_at_6 <- predict(lm_model, newdata = data.frame(time = time[6]))

else {{

detrended <- series

trend_at_6 <- 0

{

train_subset <- ts(detrended[1:5], start = min(time), frequency = 1)

true_values[j] <- series[6]

tryCatch({

ar_model <- Arima(train_subset, order = c(1, 0, 0), method = "ML")

prediction_resid <- forecast(ar_model, h = 1)$mean

predictions[j] <- prediction_resid + trend_at_6

{ , error = function(e) {

predictions[j] <- NA

({

{

predictions_denorm <- sapply(1:52, function(j) denormalize(predictions[j], paste0("TD_", j)))

true_values_denorm <- sapply(1:52, function(j) denormalize(true_values[j], paste0("TD_", j)))

rmse_eye <- sqrt(mean((true_values_denorm - predictions_denorm)^2, na.rm = TRUE))

data_rmse <- rbind(data_rmse, data.frame(eye = eye_id, rmse = rmse_eye))

mae_matrix[i, ] <- abs(true_values_denorm - predictions_denorm)

}

data_mae$mae <- colMeans(mae_matrix, na.rm = TRUE)

write_csv(data_rmse, "D:/average_rmse_AR1-5.csv")

write_csv(data_mae, "D:/average_mae_AR1-5.csv")

mae_per_eye_loc <- as.data.frame(mae_matrix_window10)

colnames(mae_per_eye_loc) <- paste0("TD_", 1:52)

write_csv(mae_per_eye_loc, "D:/average_mae_per_eye_loc_ar1_5.csv")

Supplement Material- AR(1) Model Code

S2. AR(1) Model Implementation for Examination 7 Under the Moderate Progression Rate Scenario

rm(list = ls())

gc()

library(dplyr)

library(caret)

library(readr)

library(TSA)

library(openxlsx)

library(stats)

library(forecast)

set.seed(123)

data <- read_csv("D:/felash10/USB Drive/phd/payanname phd/thesis/final.simulation/TD/models in average progression_td/data_average_ecc.csv")

norm_params <- list()

for (col in grep("^TD_", names(data), value = TRUE)) {

min_val <- min(data[[col]], na.rm = TRUE)

max_val <- max(data[[col]], na.rm = TRUE)

data[[paste0(col, "_norm")]] <- (data[[col]] - min_val) / (max_val - min_val)

norm_params[[col]] <- list(min = min_val, max = max_val)

}

denormalize <- function(x_norm, col_name) {

params <- norm_params[[gsub("_norm$", "", col_name)]]

return(x_norm * (params$max - params$min) + params$min)

}

data$eye <- rep(1:12000, each = 11)

set.seed(123)

test_eyes <- sample(1:12000, size = 0.15 * 12000)

data_test <- data %>% filter(eye %in% test_eyes)

data_rmse <- data.frame(eye = integer(), rmse = numeric())

data_mae <- data.frame(location = 1:52, mae = numeric(52))

mae_matrix <- matrix(0, nrow = length(test_eyes), ncol = 52)

for (i in seq_along(test_eyes)) {

eye_id <- test_eyes[i]

eye_data <- data_test %>% filter(eye == eye_id)

eye_data_subset <- eye_data[1:7, ]

predictions <- numeric(52)

true_values <- numeric(52)

for (j in 1:52) {

td_col <- paste0("TD_", j, "_norm")

series <- eye_data_subset[[td_col]]

time <- eye_data_subset$time

lm_model <- lm(series ~ time)

p_val <- summary(lm_model)$coefficients[2, 4]

if (!is.na(p_val) && p_val < 0.05) {

detrended <- residuals(lm_model)

trend_at_6 <- predict(lm_model, newdata = data.frame(time = time[7]))

}else {

detrended <- series

trend_at_6 <- 0

}

train_subset <- ts(detrended[1:6], start = min(time), frequency = 1)

true_values[j] <- series[7]

tryCatch({

ar_model <- Arima(train_subset, order = c(1, 0, 0), method = "ML")

prediction_resid <- forecast(ar_model, h = 1)$mean

predictions[j] <- prediction_resid + trend_at_6

} }, error = function(e)

predictions[j] <- NA

({

{

predictions_denorm <- sapply(1:52, function(j) denormalize(predictions[j], paste0("TD_", j)))

true_values_denorm <- sapply(1:52, function(j) denormalize(true_values[j], paste0("TD_", j)))

rmse_eye <- sqrt(mean((true_values_denorm - predictions_denorm)^2, na.rm = TRUE))

data_rmse <- rbind(data_rmse, data.frame(eye = eye_id, rmse = rmse_eye))

mae_matrix[i, ] <- abs(true_values_denorm - predictions_denorm)

{

data_mae$mae <- colMeans(mae_matrix, na.rm = TRUE)

write_csv(data_rmse, "D:/average_rmse_AR1-6.csv")

write_csv(data_mae, "D:/average_mae_AR1-6.csv")

mae_per_eye_loc <- as.data.frame(mae_matrix_window10)

colnames(mae_per_eye_loc) <- paste0("TD_", 1:52)

print(head(mae_per_eye_loc[, 1:5]))

write_csv(mae_per_eye_loc, "D:/average_mae_per_eye_loc_ar1_6.csv")

Supplement Material- AR(1) Model Code

S2. AR(1) Model Implementation for Examination 8 Under the Moderate Progression Rate Scenario

rm(list = ls())

gc()

library(dplyr)

library(caret)

library(readr)

library(TSA)

library(openxlsx)

library(stats)

library(forecast)

set.seed(123)

data <- read_csv("D:/felash10/USB Drive/phd/payanname phd/thesis/final.simulation/TD/models in average progression_td/data_average_ecc.csv")

norm_params <- list()

for (col in grep("^TD_", names(data), value = TRUE)) {

min_val <- min(data[[col]], na.rm = TRUE)

max_val <- max(data[[col]], na.rm = TRUE)

data[[paste0(col, "_norm")]] <- (data[[col]] - min_val) / (max_val - min_val)

norm_params[[col]] <- list(min = min_val, max = max_val)

{

denormalize <- function(x_norm, col_name) {

params <- norm_params[[gsub("_norm$", "", col_name)]]

return(x_norm * (params$max - params$min) + params$min)

{

data$eye <- rep(1:12000, each = 11)

set.seed(123)

test_eyes <- sample(1:12000, size = 0.15 * 12000)

data_test <- data %>% filter(eye %in% test_eyes)

data_rmse <- data.frame(eye = integer(), rmse = numeric())

data_mae <- data.frame(location = 1:52, mae = numeric(52))

mae_matrix <- matrix(0, nrow = length(test_eyes), ncol = 52)

for (i in seq_along(test_eyes)) {

eye_id <- test_eyes[i]

eye_data <- data_test %>% filter(eye == eye_id)

eye_data_subset <- eye_data[1:8, ]

predictions <- numeric(52)

true_values <- numeric(52)

for (j in 1:52) {

td_col <- paste0("TD_", j, "_norm")

series <- eye_data_subset[[td_col]]

time <- eye_data_subset$time

lm_model <- lm(series ~ time)

p_val <- summary(lm_model)$coefficients[2, 4]

if (!is.na(p_val) && p_val < 0.05) {

detrended <- residuals(lm_model)

trend_at_6 <- predict(lm_model, newdata = data.frame(time = time[8]))

else {{

detrended <- series

trend_at_6 <- 0

}

train_subset <- ts(detrended[1:7], start = min(time), frequency = 1)

true_values[j] <- series[8]

tryCatch({

ar_model <- Arima(train_subset, order = c(1, 0, 0), method = "ML")

prediction_resid <- forecast(ar_model, h = 1)$mean

predictions[j] <- prediction_resid + trend_at_6

,error = function(e) {{

predictions[j] <- NA

({

{

predictions_denorm <- sapply(1:52, function(j) denormalize(predictions[j], paste0("TD_", j)))

true_values_denorm <- sapply(1:52, function(j) denormalize(true_values[j], paste0("TD_", j)))

rmse_eye <- sqrt(mean((true_values_denorm - predictions_denorm)^2, na.rm = TRUE))

data_rmse <- rbind(data_rmse, data.frame(eye = eye_id, rmse = rmse_eye))

mae_matrix[i, ] <- abs(true_values_denorm - predictions_denorm)

{
mae_per_eye_loc <- as.data.frame(mae_matrix_window10)

colnames(mae_per_eye_loc) <- paste0("TD_", 1:52)

write_csv(mae_per_eye_loc, "D:/average_mae_per_eye_loc_ar1_7.csv")

Supplement Material- AR(1) Model Code

S2. AR(1) Model Implementation for Examination 9 Under the Moderate Progression Rate Scenario

rm(list = ls())

gc()

library(dplyr)

library(caret)

library(readr)

library(TSA)

library(openxlsx)

library(stats)

library(forecast)

set.seed(123)

data <- read_csv("D:/felash10/USB Drive/phd/payanname phd/thesis/final.simulation/TD/models in average progression_td/data_average_ecc.csv")

norm_params <- list()

for (col in grep("^TD_", names(data), value = TRUE)) {

min_val <- min(data[[col]], na.rm = TRUE)

max_val <- max(data[[col]], na.rm = TRUE)

data[[paste0(col, "_norm")]] <- (data[[col]] - min_val) / (max_val - min_val)

norm_params[[col]] <- list(min = min_val, max = max_val)

}

denormalize <- function(x_norm, col_name) {

params <- norm_params[[gsub("_norm$", "", col_name)]]

return(x_norm * (params$max - params$min) + params$min)

}

data$eye <- rep(1:12000, each = 11)

set.seed(123)

test_eyes <- sample(1:12000, size = 0.15 * 12000)

data_test <- data %>% filter(eye %in% test_eyes)

data_rmse <- data.frame(eye = integer(), rmse = numeric())

data_mae <- data.frame(location = 1:52, mae = numeric(52))

mae_matrix <- matrix(0, nrow = length(test_eyes), ncol = 52)

for (i in seq_along(test_eyes)) {

eye_id <- test_eyes[i]

eye_data <- data_test %>% filter(eye == eye_id)

eye_data_subset <- eye_data[1:9, ]

predictions <- numeric(52)

true_values <- numeric(52)

for (j in 1:52) {

td_col <- paste0("TD_", j, "_norm")

series <- eye_data_subset[[td_col]]

time <- eye_data_subset$time

lm_model <- lm(series ~ time)

p_val <- summary(lm_model)$coefficients[2, 4]

if (!is.na(p_val) && p_val < 0.05) {

detrended <- residuals(lm_model)

trend_at_6 <- predict(lm_model, newdata = data.frame(time = time[9]))

else {{

detrended <- series

trend_at_6 <- 0

}

train_subset <- ts(detrended[1:8], start = min(time), frequency = 1)

true_values[j] <- series[9]

tryCatch({

ar_model <- Arima(train_subset, order = c(1, 0, 0), method = "ML")

prediction_resid <- forecast(ar_model, h = 1)$mean

predictions[j] <- prediction_resid + trend_at_6

,error = function(e) {{

predictions[j] <- NA

})

}

predictions_denorm <- sapply(1:52, function(j) denormalize(predictions[j], paste0("TD_", j)))

true_values_denorm <- sapply(1:52, function(j) denormalize(true_values[j], paste0("TD_", j)))

rmse_eye <- sqrt(mean((true_values_denorm - predictions_denorm)^2, na.rm = TRUE))

data_rmse <- rbind(data_rmse, data.frame(eye = eye_id, rmse = rmse_eye))

mae_matrix[i, ] <- abs(true_values_denorm - predictions_denorm)

}

data_mae$mae <- colMeans(mae_matrix, na.rm = TRUE)

write_csv(data_rmse, "D:/average_rmse_AR1-8.csv")

write_csv(data_mae, "D:/average_mae_AR1-8.csv")

mae_per_eye_loc <- as.data.frame(mae_matrix_window10)

colnames(mae_per_eye_loc) <- paste0("TD_", 1:52)

print(head(mae_per_eye_loc[, 1:5]))

write_csv(mae_per_eye_loc, "D:/average_mae_per_eye_loc_ar1_8.csv")

Supplement Material- AR(1) Model Code

S2. AR(1) Model Implementation for Examination 10 Under the Moderate Progression Rate Scenario

rm(list = ls())

gc()

library(dplyr)

library(caret)

library(readr)

library(TSA)

library(openxlsx)

library(stats)

library(forecast)

set.seed(123)

data <- read_csv("D:/felash10/USB Drive/phd/payanname phd/thesis/final.simulation/TD/models in average progression_td/data_average_ecc.csv")

norm_params <- list()

for (col in grep("^TD_", names(data), value = TRUE)) {

min_val <- min(data[[col]], na.rm = TRUE)

max_val <- max(data[[col]], na.rm = TRUE)

data[[paste0(col, "_norm")]] <- (data[[col]] - min_val) / (max_val - min_val)

norm_params[[col]] <- list(min = min_val, max = max_val)

}

denormalize <- function(x_norm, col_name) {

params <- norm_params[[gsub("_norm$", "", col_name)]]

return(x_norm * (params$max - params$min) + params$min)

}

data$eye <- rep(1:12000, each = 11)

set.seed(123)

test_eyes <- sample(1:12000, size = 0.15 * 12000)

data_test <- data %>% filter(eye %in% test_eyes)

data_rmse <- data.frame(eye = integer(), rmse = numeric())

data_mae <- data.frame(location = 1:52, mae = numeric(52))

mae_matrix <- matrix(0, nrow = length(test_eyes), ncol = 52)

for (i in seq_along(test_eyes)) {

eye_id <- test_eyes[i]

eye_data <- data_test %>% filter(eye == eye_id)

eye_data_subset <- eye_data[1:10, ]

predictions <- numeric(52)

true_values <- numeric(52)

for (j in 1:52) {

td_col <- paste0("TD_", j, "_norm")

series <- eye_data_subset[[td_col]]

time <- eye_data_subset$time

lm_model <- lm(series ~ time)

p_val <- summary(lm_model)$coefficients[2, 4]

if (!is.na(p_val) && p_val < 0.05) {

detrended <- residuals(lm_model)

trend_at_6 <- predict(lm_model, newdata = data.frame(time = time[10]))

else {{

detrended <- series

trend_at_6 <- 0

}

train_subset <- ts(detrended[1:9], start = min(time), frequency = 1)

true_values[j] <- series[10]

tryCatch({

ar_model <- Arima(train_subset, order = c(1, 0, 0), method = "ML")

prediction_resid <- forecast(ar_model, h = 1)$mean

predictions[j] <- prediction_resid + trend_at_6

,error = function(e) {{

predictions[j] <- NA

})

}

predictions_denorm <- sapply(1:52, function(j) denormalize(predictions[j], paste0("TD_", j)))

true_values_denorm <- sapply(1:52, function(j) denormalize(true_values[j], paste0("TD_", j)))

rmse_eye <- sqrt(mean((true_values_denorm - predictions_denorm)^2, na.rm = TRUE))

data_rmse <- rbind(data_rmse, data.frame(eye = eye_id, rmse = rmse_eye))

mae_matrix[i, ] <- abs(true_values_denorm - predictions_denorm)

}

data_mae$mae <- colMeans(mae_matrix, na.rm = TRUE)

write_csv(data_rmse, "D:/average_rmse_AR1-9.csv")

write_csv(data_mae, "D:/average_mae_AR1-9.csv")

mae_per_eye_loc <- as.data.frame(mae_matrix_window10)

colnames(mae_per_eye_loc) <- paste0("TD_", 1:52)

write_csv(mae_per_eye_loc, "D:/average_mae_per_eye_loc_ar1_9.csv")

Supplement Material- AR(1) Model Code

S2. AR(1) Model Implementation for Examination 11 Under the Moderate Progression Rate Scenario

rm(list = ls())

gc()

library(dplyr)

library(caret)

library(readr)

library(TSA)

library(openxlsx)

library(stats)

library(forecast)

set.seed(123)

data <- read_csv("D:/felash10/USB Drive/phd/payanname phd/thesis/final.simulation/TD/models in average progression_td/data_average_ecc.csv")

norm_params <- list()

for (col in grep("^TD_", names(data), value = TRUE)) {

min_val <- min(data[[col]], na.rm = TRUE)

max_val <- max(data[[col]], na.rm = TRUE)

data[[paste0(col, "_norm")]] <- (data[[col]] - min_val) / (max_val - min_val)

norm_params[[col]] <- list(min = min_val, max = max_val)

{

denormalize <- function(x_norm, col_name) {

params <- norm_params[[gsub("_norm$", "", col_name)]]

return(x_norm * (params$max - params$min) + params$min)

{

data$eye <- rep(1:12000, each = 11)

set.seed(123)

test_eyes <- sample(1:12000, size = 0.15 * 12000)

data_test <- data %>% filter(eye %in% test_eyes)

data_rmse <- data.frame(eye = integer(), rmse = numeric())

data_mae <- data.frame(location = 1:52, mae = numeric(52))

mae_matrix <- matrix(0, nrow = length(test_eyes), ncol = 52)

for (i in seq_along(test_eyes)) {

eye_id <- test_eyes[i]

eye_data <- data_test %>% filter(eye == eye_id)

eye_data_subset <- eye_data[1:11, ]

predictions <- numeric(52)

true_values <- numeric(52)

for (j in 1:52) {

td_col <- paste0("TD_", j, "_norm")

series <- eye_data_subset[[td_col]]

time <- eye_data_subset$time

lm_model <- lm(series ~ time)

p_val <- summary(lm_model)$coefficients[2, 4]

if (!is.na(p_val) && p_val < 0.05) {

detrended <- residuals(lm_model)

trend_at_6 <- predict(lm_model, newdata = data.frame(time = time[11]))

}else {

detrended <- series

trend_at_6 <- 0

}

train_subset <- ts(detrended[1:10], start = min(time), frequency = 1)

true_values[j] <- series[11]

tryCatch({

ar_model <- Arima(train_subset, order = c(1, 0, 0), method = "ML")

prediction_resid <- forecast(ar_model, h = 1)$mean

predictions[j] <- prediction_resid + trend_at_6

},error = function(e) {

predictions[j] <- NA

})

}

predictions_denorm <- sapply(1:52, function(j) denormalize(predictions[j], paste0("TD_", j)))

true_values_denorm <- sapply(1:52, function(j) denormalize(true_values[j], paste0("TD_", j)))

rmse_eye <- sqrt(mean((true_values_denorm - predictions_denorm)^2, na.rm = TRUE))

data_rmse <- rbind(data_rmse, data.frame(eye = eye_id, rmse = rmse_eye))

mae_matrix[i, ] <- abs(true_values_denorm - predictions_denorm)

}

data_mae$mae <- colMeans(mae_matrix, na.rm = TRUE)

write_csv(data_rmse, "D:/average_rmse_AR1-10.csv")

write_csv(data_mae, "D:/average_mae_AR1-10.csv")

mae_per_eye_loc <- as.data.frame(mae_matrix_window10)

colnames(mae_per_eye_loc) <- paste0("TD_", 1:52)

write_csv(mae_per_eye_loc, "D:/average_mae_per_eye_loc_ar1_10.csv")

Supplement Material- AR(1) Model Code

S2. AR(1) Model Implementation for Examination 6 Under the Fast Progression Rate Scenario

rm(list = ls())

gc()

library(dplyr)

library(caret)

library(readr)

library(TSA)

library(openxlsx)

library(stats)

library(forecast)

set.seed(123)

data <- read_csv("D:/felash10/USB Drive/phd/payanname phd/thesis/final.simulation/TD/models in quick progression_td/data_quick_ecc.csv")

norm_params <- list()

for (col in grep("^TD_", names(data), value = TRUE)) {

min_val <- min(data[[col]], na.rm = TRUE)

max_val <- max(data[[col]], na.rm = TRUE)

data[[paste0(col, "_norm")]] <- (data[[col]] - min_val) / (max_val - min_val)

norm_params[[col]] <- list(min = min_val, max = max_val)

}

denormalize <- function(x_norm, col_name) {

params <- norm_params[[gsub("_norm$", "", col_name)]]

return(x_norm * (params$max - params$min) + params$min)

}

data$eye <- rep(1:12000, each = 11)

set.seed(123)

test_eyes <- sample(1:12000, size = 0.15 * 12000)

data_test <- data %>% filter(eye %in% test_eyes)

data_rmse <- data.frame(eye = integer(), rmse = numeric())

data_mae <- data.frame(location = 1:52, mae = numeric(52))

mae_matrix <- matrix(0, nrow = length(test_eyes), ncol = 52)

for (i in seq_along(test_eyes)) {

eye_id <- test_eyes[i]

eye_data <- data_test %>% filter(eye == eye_id)

eye_data_subset <- eye_data[1:6, ]

predictions <- numeric(52)

true_values <- numeric(52)

for (j in 1:52) {

td_col <- paste0("TD_", j, "_norm")

series <- eye_data_subset[[td_col]]

time <- eye_data_subset$time

lm_model <- lm(series ~ time)

p_val <- summary(lm_model)$coefficients[2, 4]

if (!is.na(p_val) && p_val < 0.05) {

detrended <- residuals(lm_model)

trend_at_6 <- predict(lm_model, newdata = data.frame(time = time[6]))

else {{

detrended <- series

trend_at_6 <- 0

{

train_subset <- ts(detrended[1:5], start = min(time), frequency = 1)

true_values[j] <- series[6]

tryCatch({

ar_model <- Arima(train_subset, order = c(1, 0, 0), method = "ML")

prediction_resid <- forecast(ar_model, h = 1)$mean

predictions[j] <- prediction_resid + trend_at_6

} },error = function(e)

predictions[j] <- NA

({

{

predictions_denorm <- sapply(1:52, function(j) denormalize(predictions[j], paste0("TD_", j)))

true_values_denorm <- sapply(1:52, function(j) denormalize(true_values[j], paste0("TD_", j)))

rmse_eye <- sqrt(mean((true_values_denorm - predictions_denorm)^2, na.rm = TRUE))

data_rmse <- rbind(data_rmse, data.frame(eye = eye_id, rmse = rmse_eye))

mae_matrix[i, ] <- abs(true_values_denorm - predictions_denorm)

{

data_mae$mae <- colMeans(mae_matrix, na.rm = TRUE)

write_csv(data_rmse, "D:/quick_rmse_AR1-5.csv")

write_csv(data_mae, "D:/quick_mae_AR1-5.csv")

mae_per_eye_loc <- as.data.frame(mae_matrix_window10)

colnames(mae_per_eye_loc) <- paste0("TD_", 1:52)

write_csv(mae_per_eye_loc, "D:/quick_mae_per_eye_loc_ar1_5.csv")

Supplement Material- AR(1) Model Code

S2. AR(1) Model Implementation for Examination 7 Under the Fast Progression Rate Scenario

library(dplyr)

library(caret)

library(readr)

library(TSA)

library(openxlsx)

library(stats)

library(forecast)

set.seed(123)

data <- read_csv("D:/felash10/USB Drive/phd/payanname phd/thesis/final.simulation/TD/models in quick progression_td/data_quick_ecc.csv")

norm_params <- list()

for (col in grep("^TD_", names(data), value = TRUE)) {

min_val <- min(data[[col]], na.rm = TRUE)

max_val <- max(data[[col]], na.rm = TRUE)

data[[paste0(col, "_norm")]] <- (data[[col]] - min_val) / (max_val - min_val)

norm_params[[col]] <- list(min = min_val, max = max_val)

}

denormalize <- function(x_norm, col_name) {

params <- norm_params[[gsub("_norm$", "", col_name)]]

return(x_norm * (params$max - params$min) + params$min)

}

data$eye <- rep(1:12000, each = 11)

set.seed(123)

test_eyes <- sample(1:12000, size = 0.15 * 12000)

data_test <- data %>% filter(eye %in% test_eyes)

data_rmse <- data.frame(eye = integer(), rmse = numeric())

data_mae <- data.frame(location = 1:52, mae = numeric(52))

mae_matrix <- matrix(0, nrow = length(test_eyes), ncol = 52)

for (i in seq_along(test_eyes)) {

eye_id <- test_eyes[i]

eye_data <- data_test %>% filter(eye == eye_id)

eye_data_subset <- eye_data[1:7, ]

predictions <- numeric(52)

true_values <- numeric(52)

for (j in 1:52) {

td_col <- paste0("TD_", j, "_norm")

series <- eye_data_subset[[td_col]]

time <- eye_data_subset$time

lm_model <- lm(series ~ time)

p_val <- summary(lm_model)$coefficients[2, 4]

if (!is.na(p_val) && p_val < 0.05) {

detrended <- residuals(lm_model)

trend_at_6 <- predict(lm_model, newdata = data.frame(time = time[7]))

else {{

detrended <- series

trend_at_6 <- 0

}

train_subset <- ts(detrended[1:6], start = min(time), frequency = 1)

true_values[j] <- series[7]

tryCatch({

ar_model <- Arima(train_subset, order = c(1, 0, 0), method = "ML")

prediction_resid <- forecast(ar_model, h = 1)$mean

predictions[j] <- prediction_resid + trend_at_6

}, error = function(e) {

predictions[j] <- NA

})

}

predictions_denorm <- sapply(1:52, function(j) denormalize(predictions[j], paste0("TD_", j)))

true_values_denorm <- sapply(1:52, function(j) denormalize(true_values[j], paste0("TD_", j)))

rmse_eye <- sqrt(mean((true_values_denorm - predictions_denorm)^2, na.rm = TRUE))

data_rmse <- rbind(data_rmse, data.frame(eye = eye_id, rmse = rmse_eye))

mae_matrix[i, ] <- abs(true_values_denorm - predictions_denorm)

}

data_mae$mae <- colMeans(mae_matrix, na.rm = TRUE)

write_csv(data_rmse, "D:/quick_rmse_AR1-6.csv")

write_csv(data_mae, "D:/quick_mae_AR1-6.csv")

mae_per_eye_loc <- as.data.frame(mae_matrix_window10)

colnames(mae_per_eye_loc) <- paste0("TD_", 1:52)

write_csv(mae_per_eye_loc, "D:/quick_mae_per_eye_loc_ar1_6.csv")

Supplement Material- AR(1) Model Code

S2. AR(1) Model Implementation for Examination 8 Under the Fast Progression Rate Scenario

rm(list = ls())

gc()

library(dplyr)

library(caret)

library(readr)

library(TSA)

library(openxlsx)

library(stats)

library(forecast)

set.seed(123)

data <- read_csv("D:/felash10/USB Drive/phd/payanname phd/thesis/final.simulation/TD/models in quick progression_td/data_quick_ecc.csv")

norm_params <- list()

for (col in grep("^TD_", names(data), value = TRUE)) {

min_val <- min(data[[col]], na.rm = TRUE)

max_val <- max(data[[col]], na.rm = TRUE)

data[[paste0(col, "_norm")]] <- (data[[col]] - min_val) / (max_val - min_val)

norm_params[[col]] <- list(min = min_val, max = max_val)

{

denormalize <- function(x_norm, col_name) {

params <- norm_params[[gsub("_norm$", "", col_name)]]

return(x_norm * (params$max - params$min) + params$min)

{

data$eye <- rep(1:12000, each = 11)

set.seed(123)

test_eyes <- sample(1:12000, size = 0.15 * 12000)

data_test <- data %>% filter(eye %in% test_eyes)

data_rmse <- data.frame(eye = integer(), rmse = numeric())

data_mae <- data.frame(location = 1:52, mae = numeric(52))

mae_matrix <- matrix(0, nrow = length(test_eyes), ncol = 52)

for (i in seq_along(test_eyes)) {

eye_id <- test_eyes[i]

eye_data <- data_test %>% filter(eye == eye_id)

eye_data_subset <- eye_data[1:8, ]

predictions <- numeric(52)

true_values <- numeric(52)

for (j in 1:52) {

td_col <- paste0("TD_", j, "_norm")

series <- eye_data_subset[[td_col]]

time <- eye_data_subset$time

lm_model <- lm(series ~ time)

p_val <- summary(lm_model)$coefficients[2, 4]

if (!is.na(p_val) && p_val < 0.05) {

detrended <- residuals(lm_model)

trend_at_6 <- predict(lm_model, newdata = data.frame(time = time[8]))

else {{

detrended <- series

trend_at_6 <- 0

}

train_subset <- ts(detrended[1:7], start = min(time), frequency = 1)

true_values[j] <- series[8]

tryCatch({

ar_model <- Arima(train_subset, order = c(1, 0, 0), method = "ML")

prediction_resid <- forecast(ar_model, h = 1)$mean

predictions[j] <- prediction_resid + trend_at_6

},error = function(e) {

predictions[j] <- NA

})

}

predictions_denorm <- sapply(1:52, function(j) denormalize(predictions[j], paste0("TD_", j)))

true_values_denorm <- sapply(1:52, function(j) denormalize(true_values[j], paste0("TD_", j)))

rmse_eye <- sqrt(mean((true_values_denorm - predictions_denorm)^2, na.rm = TRUE))

data_rmse <- rbind(data_rmse, data.frame(eye = eye_id, rmse = rmse_eye))

mae_matrix[i, ] <- abs(true_values_denorm - predictions_denorm)

}

data_mae$mae <- colMeans(mae_matrix, na.rm = TRUE)

write_csv(data_rmse, "D:/quick_rmse_AR1-7.csv")

write_csv(data_mae, "D:/quick_mae_AR1-7.csv")

mae_per_eye_loc <- as.data.frame(mae_matrix_window10)

colnames(mae_per_eye_loc) <- paste0("TD_", 1:52)

write_csv(mae_per_eye_loc, "D:/quick_mae_per_eye_loc_ar1_7.csv")

Supplement Material- AR(1) Model Code

S2. AR(1) Model Implementation for Examination 9 Under the Fast Progression Rate Scenario

rm(list = ls())

gc()

library(dplyr)

library(caret)

library(readr)

library(TSA)

library(openxlsx)

library(stats)

library(forecast)

set.seed(123)

data <- read_csv("D:/felash10/USB Drive/phd/payanname phd/thesis/final.simulation/TD/models in quick progression_td/data_quick_ecc.csv")

norm_params <- list()

for (col in grep("^TD_", names(data), value = TRUE)) {

min_val <- min(data[[col]], na.rm = TRUE)

max_val <- max(data[[col]], na.rm = TRUE)

data[[paste0(col, "_norm")]] <- (data[[col]] - min_val) / (max_val - min_val)

norm_params[[col]] <- list(min = min_val, max = max_val)

{

denormalize <- function(x_norm, col_name) {

params <- norm_params[[gsub("_norm$", "", col_name)]]

return(x_norm * (params$max - params$min) + params$min)

{

data$eye <- rep(1:12000, each = 11)

set.seed(123)

test_eyes <- sample(1:12000, size = 0.15 * 12000)

data_test <- data %>% filter(eye %in% test_eyes)

data_rmse <- data.frame(eye = integer(), rmse = numeric())

data_mae <- data.frame(location = 1:52, mae = numeric(52))

mae_matrix <- matrix(0, nrow = length(test_eyes), ncol = 52)

for (i in seq_along(test_eyes)) {

eye_id <- test_eyes[i]

eye_data <- data_test %>% filter(eye == eye_id)

eye_data_subset <- eye_data[1:9, ]

predictions <- numeric(52)

true_values <- numeric(52)

for (j in 1:52) {

td_col <- paste0("TD_", j, "_norm")

series <- eye_data_subset[[td_col]]

time <- eye_data_subset$time

lm_model <- lm(series ~ time)

p_val <- summary(lm_model)$coefficients[2, 4]

if (!is.na(p_val) && p_val < 0.05) {

detrended <- residuals(lm_model)

trend_at_6 <- predict(lm_model, newdata = data.frame(time = time[9]))

}else {

detrended <- series

trend_at_6 <- 0

}

train_subset <- ts(detrended[1:8], start = min(time), frequency = 1)

true_values[j] <- series[9]

tryCatch({

ar_model <- Arima(train_subset, order = c(1, 0, 0), method = "ML")

prediction_resid <- forecast(ar_model, h = 1)$mean

predictions[j] <- prediction_resid + trend_at_6

},error = function(e) {

predictions[j] <- NA

})

}

predictions_denorm <- sapply(1:52, function(j) denormalize(predictions[j], paste0("TD_", j)))

true_values_denorm <- sapply(1:52, function(j) denormalize(true_values[j], paste0("TD_", j)))

rmse_eye <- sqrt(mean((true_values_denorm - predictions_denorm)^2, na.rm = TRUE))

data_rmse <- rbind(data_rmse, data.frame(eye = eye_id, rmse = rmse_eye))

mae_matrix[i, ] <- abs(true_values_denorm - predictions_denorm)

}

data_mae$mae <- colMeans(mae_matrix, na.rm = TRUE)

write_csv(data_rmse, "D:/quick_rmse_AR1-8.csv")

write_csv(data_mae, "D:/quick_mae_AR1-8.csv")

mae_per_eye_loc <- as.data.frame(mae_matrix_window10)

colnames(mae_per_eye_loc) <- paste0("TD_", 1:52)

write_csv(mae_per_eye_loc, "D:/quick_mae_per_eye_loc_ar1_8.csv")

Supplement Material- AR(1) Model Code

S2. AR(1) Model Implementation for Examination 10 Under the Fast Progression Rate Scenario

rm(list = ls())

gc()

library(dplyr)

library(caret)

library(readr)

library(TSA)

library(openxlsx)

library(stats)

library(forecast)

set.seed(123)

data <- read_csv("D:/felash10/USB Drive/phd/payanname phd/thesis/final.simulation/TD/models in quick progression_td/data_quick_ecc.csv")

norm_params <- list()

for (col in grep("^TD_", names(data), value = TRUE)) {

min_val <- min(data[[col]], na.rm = TRUE)

max_val <- max(data[[col]], na.rm = TRUE)

data[[paste0(col, "_norm")]] <- (data[[col]] - min_val) / (max_val - min_val)

norm_params[[col]] <- list(min = min_val, max = max_val)

}

denormalize <- function(x_norm, col_name) {

params <- norm_params[[gsub("_norm$", "", col_name)]]

return(x_norm * (params$max - params$min) + params$min)

}

data$eye <- rep(1:12000, each = 11)

set.seed(123)

test_eyes <- sample(1:12000, size = 0.15 * 12000)

data_test <- data %>% filter(eye %in% test_eyes)

data_rmse <- data.frame(eye = integer(), rmse = numeric())

data_mae <- data.frame(location = 1:52, mae = numeric(52))

mae_matrix <- matrix(0, nrow = length(test_eyes), ncol = 52)

for (i in seq_along(test_eyes)) {

eye_id <- test_eyes[i]

eye_data <- data_test %>% filter(eye == eye_id)

eye_data_subset <- eye_data[1:10, ]

predictions <- numeric(52)

true_values <- numeric(52)

for (j in 1:52) {

td_col <- paste0("TD_", j, "_norm")

series <- eye_data_subset[[td_col]]

time <- eye_data_subset$time

lm_model <- lm(series ~ time)

p_val <- summary(lm_model)$coefficients[2, 4]

if (!is.na(p_val) && p_val < 0.05) {

detrended <- residuals(lm_model)

trend_at_6 <- predict(lm_model, newdata = data.frame(time = time[10]))

else {{

detrended <- series

trend_at_6 <- 0

}

train_subset <- ts(detrended[1:9], start = min(time), frequency = 1)

true_values[j] <- series[10]

tryCatch({

ar_model <- Arima(train_subset, order = c(1, 0, 0), method = "ML")

prediction_resid <- forecast(ar_model, h = 1)$mean

predictions[j] <- prediction_resid + trend_at_6

,error = function(e) {{

predictions[j] <- NA

})

}

predictions_denorm <- sapply(1:52, function(j) denormalize(predictions[j], paste0("TD_", j)))

true_values_denorm <- sapply(1:52, function(j) denormalize(true_values[j], paste0("TD_", j)))

rmse_eye <- sqrt(mean((true_values_denorm - predictions_denorm)^2, na.rm = TRUE))

data_rmse <- rbind(data_rmse, data.frame(eye = eye_id, rmse = rmse_eye))

mae_matrix[i, ] <- abs(true_values_denorm - predictions_denorm)

}

data_mae$mae <- colMeans(mae_matrix, na.rm = TRUE)

write_csv(data_rmse, "D:/quick_rmse_AR1-9.csv")

write_csv(data_mae, "D:/quick_mae_AR1-9.csv")

mae_matrix_window10 <- mae_matrix

mae_per_eye_loc <- as.data.frame(mae_matrix_window10)

colnames(mae_per_eye_loc) <- paste0("TD_", 1:52)

write_csv(mae_per_eye_loc, "D:/quick_mae_per_eye_loc_ar1_9.csv")

Supplement Material- AR(1) Model Code

S2. AR(1) Model Implementation for Examination 11 Under the Fast Progression Rate Scenario

library(dplyr)

library(caret)

library(readr)

library(TSA)

library(openxlsx)

library(stats)

library(forecast)

set.seed(123)

data <- read_csv("D:/felash10/USB Drive/phd/payanname phd/thesis/final.simulation/TD/models in quick progression_td/data_quick_ecc.csv")

norm_params <- list()

for (col in grep("^TD_", names(data), value = TRUE)) {

min_val <- min(data[[col]], na.rm = TRUE)

max_val <- max(data[[col]], na.rm = TRUE)

data[[paste0(col, "_norm")]] <- (data[[col]] - min_val) / (max_val - min_val)

norm_params[[col]] <- list(min = min_val, max = max_val)

{

denormalize <- function(x_norm, col_name) {

params <- norm_params[[gsub("_norm$", "", col_name)]]

return(x_norm * (params$max - params$min) + params$min)

{

data$eye <- rep(1:12000, each = 11)

set.seed(123)

test_eyes <- sample(1:12000, size = 0.15 * 12000)

data_test <- data %>% filter(eye %in% test_eyes)

data_rmse <- data.frame(eye = integer(), rmse = numeric())

data_mae <- data.frame(location = 1:52, mae = numeric(52))

mae_matrix <- matrix(0, nrow = length(test_eyes), ncol = 52)

for (i in seq_along(test_eyes)) {

eye_id <- test_eyes[i]

eye_data <- data_test %>% filter(eye == eye_id)

eye_data_subset <- eye_data[1:11, ]

predictions <- numeric(52)

true_values <- numeric(52)

for (j in 1:52) {

td_col <- paste0("TD_", j, "_norm")

series <- eye_data_subset[[td_col]]

time <- eye_data_subset$time

lm_model <- lm(series ~ time)

p_val <- summary(lm_model)$coefficients[2, 4]

if (!is.na(p_val) && p_val < 0.05) {

detrended <- residuals(lm_model)

trend_at_6 <- predict(lm_model, newdata = data.frame(time = time[11]))

}else {

detrended <- series

trend_at_6 <- 0

{

train_subset <- ts(detrended[1:10], start = min(time), frequency = 1)

true_values[j] <- series[11]

tryCatch({

ar_model <- Arima(train_subset, order = c(1, 0, 0), method = "ML")

prediction_resid <- forecast(ar_model, h = 1)$mean

predictions[j] <- prediction_resid + trend_at_6

},error = function(e) {

predictions[j] <- NA

})

}

predictions_denorm <- sapply(1:52, function(j) denormalize(predictions[j], paste0("TD_", j)))

true_values_denorm <- sapply(1:52, function(j) denormalize(true_values[j], paste0("TD_", j)))

rmse_eye <- sqrt(mean((true_values_denorm - predictions_denorm)^2, na.rm = TRUE))

data_rmse <- rbind(data_rmse, data.frame(eye = eye_id, rmse = rmse_eye))

mae_matrix[i, ] <- abs(true_values_denorm - predictions_denorm)

}

data_mae$mae <- colMeans(mae_matrix, na.rm = TRUE)

write_csv(data_rmse, "D:/quick_rmse_AR1-10.csv")

write_csv(data_mae, "D:/quick_mae_AR1-10.csv")

mae_matrix_window10 <- mae_matrix #

mae_per_eye_loc <- as.data.frame(mae_matrix_window10)

colnames(mae_per_eye_loc) <- paste0("TD_", 1:52)

write_csv(mae_per_eye_loc, "D:/quick_mae_per_eye_loc_ar1_10.csv")

Supplement Material- LSTM Model Code

S2. LSTM Model Implementation for Examination 6 Under the Slow Progression Rate Scenario

rm(list = ls())

gc()

library(dplyr)

library(caret)

library(readr)

library(tensorflow)

library(keras)

library(openxlsx)

tensorflow::set_random_seed(123)

data <- read_csv("D:/felash10/USB Drive/phd/payanname phd/thesis/final.simulation/TD/models in slow progression_td/data_slow_ecc.csv")

nvisit <- 11

neye <- 12000

num_features <- 52

start_col_TD <- 5

start_col_PD <- start_col_TD + num_features

data$eye <- rep(1:neye, each = nvisit)

normalize <- function(x) {

(x - min(x, na.rm = TRUE)) / (max(x, na.rm = TRUE) - min(x, na.rm = TRUE))

}

normalized_TD_columns <- paste0("TD_", 1:num_features, "_norm")

normalized_PD_columns <- paste0("PD_", 1:num_features, "_norm")

data[normalized_TD_columns] <- lapply(data[start_col_TD:(start_col_TD + num_features - 1)], normalize)

data[normalized_PD_columns] <- lapply(data[start_col_PD:(start_col_PD + num_features - 1)], normalize)

set.seed(123)

test_indices <- sample(1:neye, size = 0.15 * neye)

train_indices <- setdiff(1:neye, test_indices)

inputs_list <- list()

outputs_list <- list()

create_windows <- function(eye_data, is_test = FALSE) {

input_list <- list()

output_list <- list()

if (is_test) {

input <- as.matrix(eye_data[1:5, c(normalized_TD_columns, normalized_PD_columns)])

output <- as.matrix(eye_data[6, normalized_TD_columns])

input_list[[1]] <- input

output_list[[1]] <- output

}else {

for (i in 1:1) {

input <- as.matrix(eye_data[i:(i+4), c(normalized_TD_columns, normalized_PD_columns)])

output <- as.matrix(eye_data[i+5, normalized_TD_columns])

input_list[[i]] <- input

output_list[[i]] <- output

{

{

return(list(inputs = input_list, outputs = output_list))

{

for (eye_id in 1:neye) {

eye_data <- data[data$eye == eye_id, ]

windows <- create_windows(eye_data, is_test = (eye_id %in% test_indices))

inputs_list[[eye_id]] <- windows$inputs

outputs_list[[eye_id]] <- windows$outputs

{

train_inputs <- inputs_list[train_indices]

train_outputs <- outputs_list[train_indices]

test_inputs <- inputs_list[test_indices]

test_outputs <- outputs_list[test_indices]

x_train <- as.array(train_inputs, dim = c(length(train_indices), 1, 5, num_features * 2))

y_train <- as.array(train_outputs, dim = c(length(train_indices), 1, num_features))

x_test <- as.array(test_inputs, dim = c(length(test_indices), 1, 5, num_features * 2))

y_test <- as.array(test_outputs, dim = c(length(test_indices), 1, num_features))

x_train_tensor <- as_tensor(unlist(x_train),shape=c(10200,1,5,104))

y_train_tensor <- as_tensor(unlist(y_train),shape=c(10200,1,52))

x_test_tensor <- as_tensor(unlist(x_test),shape=c(1800,1,5,104))

y_test_tensor <- as_tensor(unlist(y_test),shape=c(1800,1,52))

build_model <- function() {

input <- layer_input(shape = c(NA, 5, 104))

model <- input %>%

time_distributed(layer_lstm(units = 64, return_sequences = FALSE)) %>%

layer_lstm(units = 50, return_sequences = TRUE) %>%

time_distributed(layer_dense(units = 52, activation = "linear",

kernel_regularizer = regularizer_l2(0.0001)))

model_final <- keras_model(inputs = input, outputs = model)

model_final %>% compile(

loss = "mean_squared_error",

optimizer = optimizer_adam(learning_rate = 0.0001),

metrics = c("mean_squared_error", "mean_absolute_error")

(

return(model_final)

{

model <- build_model()

early_stopping <- callback_early_stopping(

monitor = "val_loss",

patience = 4,

min_delta = 0.001,

restore_best_weights = TRUE

(

history <- model %>% fit(

x_train_tensor, y_train_tensor,

validation_split = 0.1,

epochs = 100,

batch_size = 512,

verbose = 2,

callbacks = list(early_stopping)

(

y_test_pred <- model %>% predict(x_test_tensor)

valuation_train <- model %>% evaluate(x_train_tensor, y_train_tensor, verbose = 0)

evaluation_test <- model %>% evaluate(x_test_tensor, y_test_tensor, verbose = 0)

cat("Training MSE (TD):", valuation_train[[2]], "\n")

cat("Testing MSE (TD):", evaluation_test[[2]], "\n")

library(dplyr)

library(Metrics)

y_true_array <- as.array(y_test_tensor)

y_pred_array <- as.array(y_test_pred)

y_true_1th <- y_true_array[, 1, ] # dim: num_eyes x 52

y_pred_1th <- y_pred_array[, 1, ]

denorm_true1 <- matrix(NA, nrow = nrow(y_true_1th), ncol = num_features)

denorm_pred1 <- matrix(NA, nrow = nrow(y_pred_1th), ncol = num_features)

for (i in 1:num_features) {

col_name <- paste0("TD_", i)

min_val <- min(data[[col_name]], na.rm = TRUE)

max_val <- max(data[[col_name]], na.rm = TRUE)

denorm_true1[, i] <- y_true_1th[, i] * (max_val - min_val) + min_val

denorm_pred1[, i] <- y_pred_1th[, i] * (max_val - min_val) + min_val

{

rmse_per_eye1 <- sqrt(rowMeans((denorm_pred1 - denorm_true1)^2, na.rm = TRUE))

data_rmse_window1 <- data.frame(

eye_id = test_indices,

rmse = rmse_per_eye1

(

mae_per_feature1 <- colMeans(abs(denorm_pred1 - denorm_true1), na.rm = TRUE)

data_mae_window1 <- data.frame(

location = paste0("TD_", 1:num_features),

MAE = mae_per_feature1

(

head(data_mae_window1)

write_csv(data_rmse_window1, "D:/slow_rmse_LSTM-5.csv")

write_csv(data_mae_window1, "D:/slow_mae_LSTM-5.csv")

mae_per_eye_loc <- as.data.frame(mae_matrix_window1)

colnames(mae_per_eye_loc) <- paste0("TD_", 1:num_features)

rownames(mae_per_eye_loc) <- test_indices

write_csv(mae_per_eye_loc, "D:/slow_mae_per_eye_loc_lstm_5.csv")

Supplement Material- LSTM Model Code

S2. LSTM Model Implementation for Examination 7 Under the Slow Progression Rate Scenario

rm(list = ls())

gc()

library(dplyr)

library(caret)

library(readr)

library(tensorflow)

library(keras)

library(openxlsx)

tensorflow::set_random_seed(123)

data <- read_csv("D:/felash10/USB Drive/phd/payanname phd/thesis/final.simulation/TD/models in slow progression_td/data_slow_ecc.csv")

nvisit <- 11

neye <- 12000

num_features <- 52

start_col_TD <- 5

start_col_PD <- start_col_TD + num_features

data$eye <- rep(1:neye, each = nvisit)

normalize <- function(x) {

(x - min(x, na.rm = TRUE)) / (max(x, na.rm = TRUE) - min(x, na.rm = TRUE))

}

normalized_TD_columns <- paste0("TD_", 1:num_features, "_norm")

normalized_PD_columns <- paste0("PD_", 1:num_features, "_norm")

data[normalized_TD_columns] <- lapply(data[start_col_TD:(start_col_TD + num_features - 1)], normalize)

data[normalized_PD_columns] <- lapply(data[start_col_PD:(start_col_PD + num_features - 1)], normalize)

set.seed(123)

test_indices <- sample(1:neye, size = 0.15 * neye)

train_indices <- setdiff(1:neye, test_indices)

inputs_list <- list()

outputs_list <- list()

create_windows <- function(eye_data, is_test = FALSE) {

input_list <- list()

output_list <- list()

if (is_test) {

input <- as.matrix(eye_data[2:6, c(normalized_TD_columns, normalized_PD_columns)])

output <- as.matrix(eye_data[7, normalized_TD_columns])

input_list[[1]] <- input

output_list[[1]] <- output

}else {

for (i in 1:2) {

input <- as.matrix(eye_data[i:(i+4), c(normalized_TD_columns, normalized_PD_columns)])

output <- as.matrix(eye_data[i+5, normalized_TD_columns])

input_list[[i]] <- input

output_list[[i]] <- output

}

}

return(list(inputs = input_list, outputs = output_list))

}

for (eye_id in 1:neye) {

eye_data <- data[data$eye == eye_id, ]

windows <- create_windows(eye_data, is_test = (eye_id %in% test_indices))

inputs_list[[eye_id]] <- windows$inputs

outputs_list[[eye_id]] <- windows$outputs

}

train_inputs <- inputs_list[train_indices]

train_outputs <- outputs_list[train_indices]

test_inputs <- inputs_list[test_indices]

test_outputs <- outputs_list[test_indices]

x_train <- as.array(train_inputs, dim = c(length(train_indices), 2, 5, num_features * 2))

y_train <- as.array(train_outputs, dim = c(length(train_indices), 2, num_features))

x_test <- as.array(test_inputs, dim = c(length(test_indices), 1, 5, num_features * 2))

y_test <- as.array(test_outputs, dim = c(length(test_indices), 1, num_features))

x_train_tensor <- as_tensor(unlist(x_train),shape=c(10200,2,5,104))

y_train_tensor <- as_tensor(unlist(y_train),shape=c(10200,2,52))

x_test_tensor <- as_tensor(unlist(x_test),shape=c(1800,1,5,104))

y_test_tensor <- as_tensor(unlist(y_test),shape=c(1800,1,52))

build_model <- function() {

input <- layer_input(shape = c(NA, 5, 104))

model <- input %>%

time_distributed(layer_lstm(units = 64, return_sequences = FALSE)) %>%

layer_lstm(units = 75, return_sequences = TRUE) %>%

time_distributed(layer_dense(units = 52, activation = "linear",

kernel_regularizer = regularizer_l2(0.0001)))

model_final <- keras_model(inputs = input, outputs = model)

model_final %>% compile(

loss = "mean_squared_error",

optimizer = optimizer_adam(learning_rate = 0.0001),

metrics = c("mean_squared_error", "mean_absolute_error")

)

return(model_final)

}

model <- build_model()

early_stopping <- callback_early_stopping(

monitor = "val_loss",

patience = 4,

min_delta = 0.001,

restore_best_weights = TRUE

)

history <- model %>% fit(

x_train_tensor, y_train_tensor,

validation_split = 0.1,

epochs = 100,

batch_size = 512,

verbose = 2,

callbacks = list(early_stopping)

)

y_test_pred <- model %>% predict(x_test_tensor)

valuation_train <- model %>% evaluate(x_train_tensor, y_train_tensor, verbose = 0)

evaluation_test <- model %>% evaluate(x_test_tensor, y_test_tensor, verbose = 0)

cat("Training MSE (TD):", valuation_train[[2]], "\n")

cat("Testing MSE (TD):", evaluation_test[[2]], "\n")

library(dplyr)

library(Metrics)

y_true_array <- as.array(y_test_tensor)

y_pred_array <- as.array(y_test_pred)

y_true_2th <- y_true_array[, 1, ] # dim: num_eyes x 52

y_pred_2th <- y_pred_array[, 1, ]

denorm_true2 <- matrix(NA, nrow = nrow(y_true_2th), ncol = num_features)

denorm_pred2 <- matrix(NA, nrow = nrow(y_pred_2th), ncol = num_features)

for (i in 1:num_features) {

col_name <- paste0("TD_", i)

min_val <- min(data[[col_name]], na.rm = TRUE)

max_val <- max(data[[col_name]], na.rm = TRUE)

denorm_true2[, i] <- y_true_2th[, i] * (max_val - min_val) + min_val

denorm_pred2[, i] <- y_pred_2th[, i] * (max_val - min_val) + min_val

{

rmse_per_eye2 <- sqrt(rowMeans((denorm_pred2 - denorm_true2)^2, na.rm = TRUE))

data_rmse_window2 <- data.frame(

eye_id = test_indices,

rmse = rmse_per_eye2

(

head(data_rmse_window2)

mae_per_feature2 <- colMeans(abs(denorm_pred2 - denorm_true2), na.rm = TRUE)

data_mae_window2 <- data.frame(

location = paste0("TD_", 1:num_features),

MAE = mae_per_feature2

(

head(data_mae_window2)

write_csv(data_rmse_window2, "D:/slow_rmse_LSTM-6.csv")

write_csv(data_mae_window2, "D:/slow_mae_LSTM-6.csv")

mae_matrix_window2 <- abs(denorm_pred2 - denorm_true2) #

mae_per_eye_loc <- as.data.frame(mae_matrix_window2)

colnames(mae_per_eye_loc) <- paste0("TD_", 1:num_features)

rownames(mae_per_eye_loc) <- test_indices

write_csv(mae_per_eye_loc, "D:/slow_mae_per_eye_loc_lstm_6.csv")

Supplement Material- LSTM Model Code

S2. LSTM Model Implementation for Examination 8 Under the Slow Progression Rate Scenario

rm(list = ls())

gc()

library(dplyr)

library(caret)

library(readr)

library(tensorflow)

library(keras)

library(openxlsx)

tensorflow::set_random_seed(123)

data <- read_csv("D:/felash10/USB Drive/phd/payanname phd/thesis/final.simulation/TD/models in slow progression_td/data_slow_ecc.csv")

nvisit <- 11

neye <- 12000

num_features <- 52

start_col_TD <- 5

start_col_PD <- start_col_TD + num_features

data$eye <- rep(1:neye, each = nvisit)

normalize <- function(x) {

(x - min(x, na.rm = TRUE)) / (max(x, na.rm = TRUE) - min(x, na.rm = TRUE))

{

normalized_TD_columns <- paste0("TD_", 1:num_features, "_norm")

normalized_PD_columns <- paste0("PD_", 1:num_features, "_norm")

data[normalized_TD_columns] <- lapply(data[start_col_TD:(start_col_TD + num_features - 1)], normalize)

data[normalized_PD_columns] <- lapply(data[start_col_PD:(start_col_PD + num_features - 1)], normalize)

set.seed(123)

test_indices <- sample(1:neye, size = 0.15 * neye)

train_indices <- setdiff(1:neye, test_indices)

inputs_list <- list()

outputs_list <- list()

create_windows <- function(eye_data, is_test = FALSE) {

input_list <- list()

output_list <- list()

if (is_test) {

input <- as.matrix(eye_data[3:7, c(normalized_TD_columns, normalized_PD_columns)])

output <- as.matrix(eye_data[8, normalized_TD_columns])

input_list[[1]] <- input

output_list[[1]] <- output

}else {

for (i in 1:3) {

input <- as.matrix(eye_data[i:(i+4), c(normalized_TD_columns, normalized_PD_columns)])

output <- as.matrix(eye_data[i+5, normalized_TD_columns])

input_list[[i]] <- input

output_list[[i]] <- output

{

{

return(list(inputs = input_list, outputs = output_list))

{

for (eye_id in 1:neye) {

eye_data <- data[data$eye == eye_id, ]

windows <- create_windows(eye_data, is_test = (eye_id %in% test_indices))

inputs_list[[eye_id]] <- windows$inputs

outputs_list[[eye_id]] <- windows$outputs

{

train_inputs <- inputs_list[train_indices]

train_outputs <- outputs_list[train_indices]

test_inputs <- inputs_list[test_indices]

test_outputs <- outputs_list[test_indices]

x_train <- as.array(train_inputs, dim = c(length(train_indices), 3, 5, num_features * 2))

y_train <- as.array(train_outputs, dim = c(length(train_indices), 3, num_features))

x_test <- as.array(test_inputs, dim = c(length(test_indices), 1, 5, num_features * 2))

y_test <- as.array(test_outputs, dim = c(length(test_indices), 1, num_features))

x_train_tensor <- as_tensor(unlist(x_train),shape=c(10200,3,5,104))

y_train_tensor <- as_tensor(unlist(y_train),shape=c(10200,3,52))

x_test_tensor <- as_tensor(unlist(x_test),shape=c(1800,1,5,104))

y_test_tensor <- as_tensor(unlist(y_test),shape=c(1800,1,52))

build_model <- function() {

input <- layer_input(shape = c(NA, 5, 104))

model <- input %>%

time_distributed(layer_lstm(units = 64, return_sequences = FALSE)) %>%

layer_lstm(units = 85, return_sequences = TRUE) %>%

time_distributed(layer_dense(units = 52, activation = "linear",

kernel_regularizer = regularizer_l2(0.0001)))

model_final <- keras_model(inputs = input, outputs = model)

model_final %>% compile(

loss = "mean_squared_error",

optimizer = optimizer_adam(learning_rate = 0.0001),

metrics = c("mean_squared_error", "mean_absolute_error")

(

return(model_final)

{

model <- build_model()

early_stopping <- callback_early_stopping(

monitor = "val_loss",

patience = 4,

min_delta = 0.001,

restore_best_weights = TRUE

(

history <- model %>% fit(

x_train_tensor, y_train_tensor,

validation_split = 0.1,

epochs = 100,

batch_size = 512,

verbose = 2,

callbacks = list(early_stopping)

(

y_test_pred <- model %>% predict(x_test_tensor)

valuation_train <- model %>% evaluate(x_train_tensor, y_train_tensor, verbose = 0)

evaluation_test <- model %>% evaluate(x_test_tensor, y_test_tensor, verbose = 0)

cat("Training MSE (TD):", valuation_train[[2]], "\n")

cat("Testing MSE (TD):", evaluation_test[[2]], "\n")

library(dplyr)

library(Metrics)

y_true_array <- as.array(y_test_tensor)

y_pred_array <- as.array(y_test_pred)

y_true_3th <- y_true_array[, 1, ]

y_pred_3th <- y_pred_array[, 1, ]

denorm_true3 <- matrix(NA, nrow = nrow(y_true_3th), ncol = num_features)

denorm_pred3 <- matrix(NA, nrow = nrow(y_pred_3th), ncol = num_features)

for (i in 1:num_features) {

col_name <- paste0("TD_", i)

min_val <- min(data[[col_name]], na.rm = TRUE)

max_val <- max(data[[col_name]], na.rm = TRUE)

denorm_true3[, i] <- y_true_3th[, i] * (max_val - min_val) + min_val

denorm_pred3[, i] <- y_pred_3th[, i] * (max_val - min_val) + min_val

{

rmse_per_eye3 <- sqrt(rowMeans((denorm_pred3 - denorm_true3)^2, na.rm = TRUE))

data_rmse_window3 <- data.frame(

eye_id = test_indices,

rmse = rmse_per_eye3

(

head(data_rmse_window3)

mae_per_feature3 <- colMeans(abs(denorm_pred3 - denorm_true3), na.rm = TRUE)

data_mae_window3 <- data.frame(

location = paste0("TD_", 1:num_features),

MAE = mae_per_feature3

(

head(data_mae_window3)

write_csv(data_rmse_window3, "D:/slow_rmse_LSTM-7.csv")

write_csv(data_mae_window3, "D:/slow_mae_LSTM-7.csv")

mae_per_eye_loc <- as.data.frame(mae_matrix_window3)

colnames(mae_per_eye_loc) <- paste0("TD_", 1:num_features)

write_csv(mae_per_eye_loc, "D:/slow_mae_per_eye_loc_lstm_7.csv")

Supplement Material- LSTM Model Code

S2. LSTM Model Implementation for Examination 9 Under the Slow Progression Rate Scenario

rm(list = ls())

gc()

library(dplyr)

library(caret)

library(readr)

library(tensorflow)

library(keras)

library(openxlsx)

tensorflow::set_random_seed(123)

data <- read_csv("D:/felash10/USB Drive/phd/payanname phd/thesis/final.simulation/TD/models in slow progression_td/data_slow_ecc.csv")

nvisit <- 11

neye <- 12000

num_features <- 52

start_col_TD <- 5

start_col_PD <- start_col_TD + num_features

data$eye <- rep(1:neye, each = nvisit)

normalize <- function(x) {

(x - min(x, na.rm = TRUE)) / (max(x, na.rm = TRUE) - min(x, na.rm = TRUE))

}

normalized_TD_columns <- paste0("TD_", 1:num_features, "_norm")

normalized_PD_columns <- paste0("PD_", 1:num_features, "_norm")

data[normalized_TD_columns] <- lapply(data[start_col_TD:(start_col_TD + num_features - 1)], normalize)

data[normalized_PD_columns] <- lapply(data[start_col_PD:(start_col_PD + num_features - 1)], normalize)

set.seed(123)

test_indices <- sample(1:neye, size = 0.15 * neye)

train_indices <- setdiff(1:neye, test_indices)

inputs_list <- list()

outputs_list <- list()

create_windows <- function(eye_data, is_test = FALSE) {

input_list <- list()

output_list <- list()

if (is_test) {

input <- as.matrix(eye_data[4:8, c(normalized_TD_columns, normalized_PD_columns)])

output <- as.matrix(eye_data[9, normalized_TD_columns])

input_list[[1]] <- input

output_list[[1]] <- output

else {{

for (i in 1:4) {

input <- as.matrix(eye_data[i:(i+4), c(normalized_TD_columns, normalized_PD_columns)])

output <- as.matrix(eye_data[i+5, normalized_TD_columns])

input_list[[i]] <- input

output_list[[i]] <- output

{

{

return(list(inputs = input_list, outputs = output_list))

{

for (eye_id in 1:neye) {

eye_data <- data[data$eye == eye_id, ]

windows <- create_windows(eye_data, is_test = (eye_id %in% test_indices))

inputs_list[[eye_id]] <- windows$inputs

outputs_list[[eye_id]] <- windows$outputs

{

train_inputs <- inputs_list[train_indices]

train_outputs <- outputs_list[train_indices]

test_inputs <- inputs_list[test_indices]

test_outputs <- outputs_list[test_indices]

x_train <- as.array(train_inputs, dim = c(length(train_indices), 4, 5, num_features * 2))

y_train <- as.array(train_outputs, dim = c(length(train_indices), 4, num_features))

x_test <- as.array(test_inputs, dim = c(length(test_indices), 1, 5, num_features * 2))

y_test <- as.array(test_outputs, dim = c(length(test_indices), 1, num_features))

x_train_tensor <- as_tensor(unlist(x_train),shape=c(10200,4,5,104))

y_train_tensor <- as_tensor(unlist(y_train),shape=c(10200,4,52))

x_test_tensor <- as_tensor(unlist(x_test),shape=c(1800,1,5,104))

y_test_tensor <- as_tensor(unlist(y_test),shape=c(1800,1,52))

build_model <- function() {

input <- layer_input(shape = c(NA, 5, 104))

model <- input %>%

time_distributed(layer_lstm(units = 64, return_sequences = FALSE)) %>%

layer_lstm(units = 115, return_sequences = TRUE) %>%

layer_dropout(rate = 0.1) %>%

time_distributed(layer_dense(units = 52, activation = "linear",

kernel_regularizer = regularizer_l2(0.0001)))

model_final <- keras_model(inputs = input, outputs = model)

model_final %>% compile(

loss = "mean_squared_error",

optimizer = optimizer_adam(learning_rate = 0.0001),

metrics = c("mean_squared_error", "mean_absolute_error")

(

return(model_final)

{

model <- build_model()

early_stopping <- callback_early_stopping(

monitor = "val_loss",

patience = 4,

min_delta = 0.001,

restore_best_weights = TRUE

(

history <- model %>% fit(

x_train_tensor, y_train_tensor,

validation_split = 0.1,

epochs = 100,

batch_size = 512,

verbose = 2,

callbacks = list(early_stopping)

(

y_test_pred <- model %>% predict(x_test_tensor)

valuation_train <- model %>% evaluate(x_train_tensor, y_train_tensor, verbose = 0)

evaluation_test <- model %>% evaluate(x_test_tensor, y_test_tensor, verbose = 0)

cat("Training MSE (TD):", valuation_train[[2]], "\n")

cat("Testing MSE (TD):", evaluation_test[[2]], "\n")

library(dplyr)

library(Metrics)

y_true_array <- as.array(y_test_tensor)

y_pred_array <- as.array(y_test_pred)

y_true_4th <- y_true_array[, 1, ] # dim: num_eyes x 52

y_pred_4th <- y_pred_array[, 1, ]

denorm_true4 <- matrix(NA, nrow = nrow(y_true_4th), ncol = num_features)

denorm_pred4 <- matrix(NA, nrow = nrow(y_pred_4th), ncol = num_features)

for (i in 1:num_features) {

col_name <- paste0("TD_", i)

min_val <- min(data[[col_name]], na.rm = TRUE)

max_val <- max(data[[col_name]], na.rm = TRUE)

denorm_true4[, i] <- y_true_4th[, i] * (max_val - min_val) + min_val

denorm_pred4[, i] <- y_pred_4th[, i] * (max_val - min_val) + min_val

{

rmse_per_eye4 <- sqrt(rowMeans((denorm_pred4 - denorm_true4)^2, na.rm = TRUE))

data_rmse_window4 <- data.frame(

eye_id = test_indices,

rmse = rmse_per_eye4

(

head(data_rmse_window4)

mae_per_feature4 <- colMeans(abs(denorm_pred4 - denorm_true4), na.rm = TRUE)

data_mae_window4 <- data.frame(

location = paste0("TD_", 1:num_features),

MAE = mae_per_feature4

(

head(data_mae_window4)

write_csv(data_rmse_window4, "D:/slow_rmse_LSTM-8.csv")

write_csv(data_mae_window4, "D:/slow_mae_LSTM-8.csv")

mae_matrix_window4 <- abs(denorm_pred4 - denorm_true4)

mae_per_eye_loc <- as.data.frame(mae_matrix_window4)

colnames(mae_per_eye_loc) <- paste0("TD_", 1:num_features)

rownames(mae_per_eye_loc) <- test_indices

write_csv(mae_per_eye_loc, "D:/slow_mae_per_eye_loc_lstm_8.csv")

Supplement Material- LSTM Model Code

S2. LSTM Model Implementation for Examination 10 Under the Slow Progression Rate Scenario

rm(list = ls())

gc()

library(dplyr)

library(caret)

library(readr)

library(tensorflow)

library(keras)

library(openxlsx)

tensorflow::set_random_seed(123)

data <- read_csv("D:/felash10/USB Drive/phd/payanname phd/thesis/final.simulation/TD/models in slow progression_td/data_slow_ecc.csv")

nvisit <- 11

neye <- 12000

num_features <- 52

start_col_TD <- 5

start_col_PD <- start_col_TD + num_features

data$eye <- rep(1:neye, each = nvisit)

normalize <- function(x) {

(x - min(x, na.rm = TRUE)) / (max(x, na.rm = TRUE) - min(x, na.rm = TRUE))

{

normalized_TD_columns <- paste0("TD_", 1:num_features, "_norm")

normalized_PD_columns <- paste0("PD_", 1:num_features, "_norm")

data[normalized_TD_columns] <- lapply(data[start_col_TD:(start_col_TD + num_features - 1)], normalize)

data[normalized_PD_columns] <- lapply(data[start_col_PD:(start_col_PD + num_features - 1)], normalize)

set.seed(123)

test_indices <- sample(1:neye, size = 0.15 * neye)

train_indices <- setdiff(1:neye, test_indices)

inputs_list <- list()

outputs_list <- list()

create_windows <- function(eye_data, is_test = FALSE) {

input_list <- list()

output_list <- list()

if (is_test) {

input <- as.matrix(eye_data[5:9, c(normalized_TD_columns, normalized_PD_columns)])

output <- as.matrix(eye_data[10, normalized_TD_columns])

input_list[[1]] <- input

output_list[[1]] <- output

else {{

for (i in 1:5) {

input <- as.matrix(eye_data[i:(i+4), c(normalized_TD_columns, normalized_PD_columns)])

output <- as.matrix(eye_data[i+5, normalized_TD_columns])

input_list[[i]] <- input

output_list[[i]] <- output

}

}

return(list(inputs = input_list, outputs = output_list))

}

for (eye_id in 1:neye) {

eye_data <- data[data$eye == eye_id, ]

windows <- create_windows(eye_data, is_test = (eye_id %in% test_indices))

inputs_list[[eye_id]] <- windows$inputs

outputs_list[[eye_id]] <- windows$outputs

}

train_inputs <- inputs_list[train_indices]

train_outputs <- outputs_list[train_indices]

test_inputs <- inputs_list[test_indices]

test_outputs <- outputs_list[test_indices]

x_train <- as.array(train_inputs, dim = c(length(train_indices), 5, 5, num_features * 2))

y_train <- as.array(train_outputs, dim = c(length(train_indices), 5, num_features))

x_test <- as.array(test_inputs, dim = c(length(test_indices), 1, 5, num_features * 2))

y_test <- as.array(test_outputs, dim = c(length(test_indices), 1, num_features))

x_train_tensor <- as_tensor(unlist(x_train),shape=c(10200,5,5,104))

y_train_tensor <- as_tensor(unlist(y_train),shape=c(10200,5,52))

x_test_tensor <- as_tensor(unlist(x_test),shape=c(1800,1,5,104))

y_test_tensor <- as_tensor(unlist(y_test),shape=c(1800,1,52))

build_model <- function() {

input <- layer_input(shape = c(NA, 5, 104))

model <- input %>%

time_distributed(layer_lstm(units = 64, return_sequences = FALSE)) %>%

layer_lstm(units = 115, return_sequences = TRUE) %>%

layer_dropout(rate = 0.1) %>%

time_distributed(layer_dense(units = 52, activation = "linear",

kernel_regularizer = regularizer_l2(0.0001)))

model_final <- keras_model(inputs = input, outputs = model)

model_final %>% compile(

loss = "mean_squared_error",

optimizer = optimizer_adam(learning_rate = 0.0001),

metrics = c("mean_squared_error", "mean_absolute_error")

)

return(model_final)

}

model <- build_model()

early_stopping <- callback_early_stopping(

monitor = "val_loss",

patience = 4,

min_delta = 0.001,

restore_best_weights = TRUE

)

history <- model %>% fit(

x_train_tensor, y_train_tensor,

validation_split = 0.1,

epochs = 100,

batch_size = 512,

verbose = 2,

callbacks = list(early_stopping)

)

y_test_pred <- model %>% predict(x_test_tensor)

valuation_train <- model %>% evaluate(x_train_tensor, y_train_tensor, verbose = 0)

evaluation_test <- model %>% evaluate(x_test_tensor, y_test_tensor, verbose = 0)

cat("Training MSE (TD):", valuation_train[[2]], "\n")

cat("Testing MSE (TD):", evaluation_test[[2]], "\n")

library(dplyr)

library(Metrics)

y_true_array <- as.array(y_test_tensor)

y_pred_array <- as.array(y_test_pred)

y_true_5th <- y_true_array[, 1, ] # dim: num_eyes x 52

y_pred_5th <- y_pred_array[, 1, ]

denorm_true5 <- matrix(NA, nrow = nrow(y_true_5th), ncol = num_features)

denorm_pred5 <- matrix(NA, nrow = nrow(y_pred_5th), ncol = num_features)

for (i in 1:num_features) {

col_name <- paste0("TD_", i)

min_val <- min(data[[col_name]], na.rm = TRUE)

max_val <- max(data[[col_name]], na.rm = TRUE)

denorm_true5[, i] <- y_true_5th[, i] * (max_val - min_val) + min_val

denorm_pred5[, i] <- y_pred_5th[, i] * (max_val - min_val) + min_val

}

rmse_per_eye5 <- sqrt(rowMeans((denorm_pred5 - denorm_true5)^2, na.rm = TRUE))

data_rmse_window5 <- data.frame(

eye_id = test_indices,

rmse = rmse_per_eye5

)

head(data_rmse_window5)

mae_per_feature5 <- colMeans(abs(denorm_pred5 - denorm_true5), na.rm = TRUE)

data_mae_window5 <- data.frame(

location = paste0("TD_", 1:num_features),

MAE = mae_per_feature5

)

head(data_mae_window5)

write_csv(data_rmse_window5, "D:/slow_rmse_LSTM-9.csv")

write_csv(data_mae_window5, "D:/slow_mae_LSTM-9.csv")

mae_matrix_window5 <- abs(denorm_pred5 - denorm_true5) #

mae_per_eye_loc <- as.data.frame(mae_matrix_window5)

colnames(mae_per_eye_loc) <- paste0("TD_", 1:num_features)

rownames(mae_per_eye_loc) <- test_indices

write_csv(mae_per_eye_loc, "D:/slow_mae_per_eye_loc_lstm_9.csv")

Supplement Material- LSTM Model Code

S2. LSTM Model Implementation for Examination 11 Under the Slow Progression Rate Scenario

rm(list = ls())

gc()

library(dplyr)

library(caret)

library(readr)

library(tensorflow)

library(keras)

library(openxlsx)

tensorflow::set_random_seed(123)

data <- read_csv("D:/felash10/USB Drive/phd/payanname phd/thesis/final.simulation/TD/models in slow progression_td/data_slow_ecc.csv")

nvisit <- 11

neye <- 12000

num_features <- 52

start_col_TD <- 5

start_col_PD <- start_col_TD + num_features

data$eye <- rep(1:neye, each = nvisit)

normalize <- function(x) {

(x - min(x, na.rm = TRUE)) / (max(x, na.rm = TRUE) - min(x, na.rm = TRUE))

}

normalized_TD_columns <- paste0("TD_", 1:num_features, "_norm")

normalized_PD_columns <- paste0("PD_", 1:num_features, "_norm")

data[normalized_TD_columns] <- lapply(data[start_col_TD:(start_col_TD + num_features - 1)], normalize)

data[normalized_PD_columns] <- lapply(data[start_col_PD:(start_col_PD + num_features - 1)], normalize)

set.seed(123)

test_indices <- sample(1:neye, size = 0.15 * neye)

train_indices <- setdiff(1:neye, test_indices)

inputs_list <- list()

outputs_list <- list()

create_windows <- function(eye_data, is_test = FALSE) {

input_list <- list()

output_list <- list()

if (is_test) {

input <- as.matrix(eye_data[6:10, c(normalized_TD_columns, normalized_PD_columns)])

output <- as.matrix(eye_data[11, normalized_TD_columns])

input_list[[1]] <- input

output_list[[1]] <- output

}else {

for (i in 1:6) {

input <- as.matrix(eye_data[i:(i+4), c(normalized_TD_columns, normalized_PD_columns)])

output <- as.matrix(eye_data[i+5, normalized_TD_columns])

input_list[[i]] <- input

output_list[[i]] <- output

{

{

return(list(inputs = input_list, outputs = output_list))

{

for (eye_id in 1:neye) {

eye_data <- data[data$eye == eye_id, ]

windows <- create_windows(eye_data, is_test = (eye_id %in% test_indices))

inputs_list[[eye_id]] <- windows$inputs

outputs_list[[eye_id]] <- windows$outputs

{

train_inputs <- inputs_list[train_indices]

train_outputs <- outputs_list[train_indices]

test_inputs <- inputs_list[test_indices]

test_outputs <- outputs_list[test_indices]

x_train_tensor <- as_tensor(unlist(x_train),shape=c(10200,6,5,104))

y_train_tensor <- as_tensor(unlist(y_train),shape=c(10200,6,52))

x_test_tensor <- as_tensor(unlist(x_test),shape=c(1800,1,5,104))

y_test_tensor <- as_tensor(unlist(y_test),shape=c(1800,1,52))

build_model <- function() {

input <- layer_input(shape = c(NA, 5, 104))

model <- input %>%

time_distributed(layer_lstm(units = 64, return_sequences = FALSE)) %>%

layer_lstm(units = 140, return_sequences = TRUE) %>%

layer_dropout(rate = 0.1) %>%

time_distributed(layer_dense(units = 52, activation = "linear",

kernel_regularizer = regularizer_l2(0.0001)))

model_final <- keras_model(inputs = input, outputs = model)

model_final %>% compile(

loss = "mean_squared_error",

optimizer = optimizer_adam(learning_rate = 0.0001),

metrics = c("mean_squared_error", "mean_absolute_error")

(

return(model_final)

{

model <- build_model()

early_stopping <- callback_early_stopping(

monitor = "val_loss",

patience = 4,

min_delta = 0.001,

restore_best_weights = TRUE

(

history <- model %>% fit(

x_train_tensor, y_train_tensor,

validation_split = 0.1,

epochs = 100,

batch_size = 512,

verbose = 2,

callbacks = list(early_stopping)

(

y_test_pred <- model %>% predict(x_test_tensor)

valuation_train <- model %>% evaluate(x_train_tensor, y_train_tensor, verbose = 0)

evaluation_test <- model %>% evaluate(x_test_tensor, y_test_tensor, verbose = 0)

cat("Training MSE (TD):", valuation_train[[2]], "\n")

cat("Testing MSE (TD):", evaluation_test[[2]], "\n")

x_train <- as.array(train_inputs, dim = c(length(train_indices), 6, 5, num_features * 2))

y_train <- as.array(train_outputs, dim = c(length(train_indices), 6, num_features))

x_test <- as.array(test_inputs, dim = c(length(test_indices), 6, 1, num_features * 2))

y_test <- as.array(test_outputs, dim = c(length(test_indices), 1, num_features))

library(dplyr)

library(Metrics)

y_true_array <- as.array(y_test_tensor)

y_pred_array <- as.array(y_test_pred)

y_true_6th <- y_true_array[, 1, ] # dim: num_eyes x 52

y_pred_6th <- y_pred_array[, 1, ]

denorm_true6 <- matrix(NA, nrow = nrow(y_true_6th), ncol = num_features)

denorm_pred6 <- matrix(NA, nrow = nrow(y_pred_6th), ncol = num_features)

for (i in 1:num_features) {

col_name <- paste0("TD_", i)

min_val <- min(data[[col_name]], na.rm = TRUE)

max_val <- max(data[[col_name]], na.rm = TRUE)

denorm_true6[, i] <- y_true_6th[, i] * (max_val - min_val) + min_val

denorm_pred6[, i] <- y_pred_6th[, i] * (max_val - min_val) + min_val

{

rmse_per_eye6 <- sqrt(rowMeans((denorm_pred6 - denorm_true6)^2, na.rm = TRUE))

data_rmse_window6 <- data.frame(

eye_id = test_indices,

rmse = rmse_per_eye6

(

head(data_rmse_window6)

mae_per_feature6 <- colMeans(abs(denorm_pred6 - denorm_true6), na.rm = TRUE)

data_mae_window6 <- data.frame(

location = paste0("TD_", 1:num_features),

MAE = mae_per_feature6

(

head(data_mae_window6)

write_csv(data_rmse_window6, "D:/slow_rmse_LSTM-10.csv")

write_csv(data_mae_window6, "D:/slow_mae_LSTM-10.csv")

mae_matrix_window6 <- abs(denorm_pred6 - denorm_true6)

mae_per_eye_loc <- as.data.frame(mae_matrix_window6)

colnames(mae_per_eye_loc) <- paste0("TD_", 1:num_features)

rownames(mae_per_eye_loc) <- test_indices

write_csv(mae_per_eye_loc, "D:/slow_mae_per_eye_loc_lstm_10.csv")

Supplement Material- LSTM Model Code

S2. LSTM Model Implementation for Examination 6 Under the Moderate Progression Rate Scenario

rm(list = ls())

gc()

library(dplyr)

library(caret)

library(readr)

library(tensorflow)

library(keras)

library(openxlsx)

tensorflow::set_random_seed(123)

data <- read_csv("D:/felash10/USB Drive/phd/payanname phd/thesis/final.simulation/TD/models in average progression_td/data_average_ecc.csv")

nvisit <- 11

neye <- 12000

num_features <- 52

start_col_TD <- 5

start_col_PD <- start_col_TD + num_features

data$eye <- rep(1:neye, each = nvisit)

normalize <- function(x) {

(x - min(x, na.rm = TRUE)) / (max(x, na.rm = TRUE) - min(x, na.rm = TRUE))

{

normalized_TD_columns <- paste0("TD_", 1:num_features, "_norm")

normalized_PD_columns <- paste0("PD_", 1:num_features, "_norm")

data[normalized_TD_columns] <- lapply(data[start_col_TD:(start_col_TD + num_features - 1)], normalize)

data[normalized_PD_columns] <- lapply(data[start_col_PD:(start_col_PD + num_features - 1)], normalize)

set.seed(123)

test_indices <- sample(1:neye, size = 0.15 * neye)

train_indices <- setdiff(1:neye, test_indices)

inputs_list <- list()

outputs_list <- list()

create_windows <- function(eye_data, is_test = FALSE) {

input_list <- list()

output_list <- list()

if (is_test) {

input <- as.matrix(eye_data[1:5, c(normalized_TD_columns, normalized_PD_columns)])

output <- as.matrix(eye_data[6, normalized_TD_columns])

input_list[[1]] <- input

output_list[[1]] <- output

}else {

for (i in 1:1) {

input <- as.matrix(eye_data[i:(i+4), c(normalized_TD_columns, normalized_PD_columns)])

output <- as.matrix(eye_data[i+5, normalized_TD_columns])

input_list[[i]] <- input

output_list[[i]] <- output

}

}

return(list(inputs = input_list, outputs = output_list))

}

for (eye_id in 1:neye) {

eye_data <- data[data$eye == eye_id, ]

windows <- create_windows(eye_data, is_test = (eye_id %in% test_indices))

inputs_list[[eye_id]] <- windows$inputs

outputs_list[[eye_id]] <- windows$outputs

}

train_inputs <- inputs_list[train_indices]

train_outputs <- outputs_list[train_indices]

test_inputs <- inputs_list[test_indices]

test_outputs <- outputs_list[test_indices]

x_train <- as.array(train_inputs, dim = c(length(train_indices), 1, 5, num_features * 2))

y_train <- as.array(train_outputs, dim = c(length(train_indices), 1, num_features))

x_test <- as.array(test_inputs, dim = c(length(test_indices), 1, 5, num_features * 2))

y_test <- as.array(test_outputs, dim = c(length(test_indices), 1, num_features))

x_train_tensor <- as_tensor(unlist(x_train),shape=c(10200,1,5,104))

y_train_tensor <- as_tensor(unlist(y_train),shape=c(10200,1,52))

x_test_tensor <- as_tensor(unlist(x_test),shape=c(1800,1,5,104))

y_test_tensor <- as_tensor(unlist(y_test),shape=c(1800,1,52))

build_model <- function() {

input <- layer_input(shape = c(NA, 5, 104))

model <- input %>%

time_distributed(layer_lstm(units = 64, return_sequences = FALSE)) %>%

layer_lstm(units = 30, return_sequences = TRUE) %>%

layer_dropout(rate = 0.1) %>%

time_distributed(layer_dense(units = 52, activation = "linear",

kernel_regularizer = regularizer_l2(0.0001)))

model_final <- keras_model(inputs = input, outputs = model)

model_final %>% compile(

loss = "mean_squared_error",

optimizer = optimizer_adam(learning_rate = 0.0001),

metrics = c("mean_squared_error", "mean_absolute_error")

)

return(model_final)

}

model <- build_model()

early_stopping <- callback_early_stopping(

monitor = "val_loss",

patience = 4,

min_delta = 0.001,

restore_best_weights = TRUE

)

history <- model %>% fit(

x_train_tensor, y_train_tensor,

validation_split = 0.1,

epochs = 100,

batch_size = 512,

verbose = 2,

callbacks = list(early_stopping)

)

y_test_pred <- model %>% predict(x_test_tensor)

valuation_train <- model %>% evaluate(x_train_tensor, y_train_tensor, verbose = 0)

evaluation_test <- model %>% evaluate(x_test_tensor, y_test_tensor, verbose = 0)

cat("Training MSE (TD):", valuation_train[[2]], "\n")

cat("Testing MSE (TD):", evaluation_test[[2]], "\n")

library(dplyr)

library(Metrics)

y_true_array <- as.array(y_test_tensor)

y_pred_array <- as.array(y_test_pred)

y_true_1th <- y_true_array[, 1, ] # dim: num_eyes x 52

y_pred_1th <- y_pred_array[, 1, ]

denorm_true1 <- matrix(NA, nrow = nrow(y_true_1th), ncol = num_features)

denorm_pred1 <- matrix(NA, nrow = nrow(y_pred_1th), ncol = num_features)

for (i in 1:num_features) {

col_name <- paste0("TD_", i)

min_val <- min(data[[col_name]], na.rm = TRUE)

max_val <- max(data[[col_name]], na.rm = TRUE)

denorm_true1[, i] <- y_true_1th[, i] * (max_val - min_val) + min_val

denorm_pred1[, i] <- y_pred_1th[, i] * (max_val - min_val) + min_val

{

rmse_per_eye1 <- sqrt(rowMeans((denorm_pred1 - denorm_true1)^2, na.rm = TRUE))

data_rmse_window1 <- data.frame(

eye_id = test_indices,

rmse = rmse_per_eye1

(

head(data_rmse_window1)

mae_per_feature1 <- colMeans(abs(denorm_pred1 - denorm_true1), na.rm = TRUE)

data_mae_window1 <- data.frame(

location = paste0("TD_", 1:num_features),

MAE = mae_per_feature1

(

head(data_mae_window1)

write_csv(data_rmse_window1, "D:/average_rmse_LSTM-5.csv")

write_csv(data_mae_window1, "D:/average_mae_LSTM-5.csv")

mae_per_eye_loc <- as.data.frame(mae_matrix_window1)

colnames(mae_per_eye_loc) <- paste0("TD_", 1:num_features)

write_csv(mae_per_eye_loc, "D:/average_mae_per_eye_loc_lstm_5.csv")

Supplement Material- LSTM Model Code

S2. LSTM Model Implementation for Examination 7 Under the Moderate Progression Rate Scenario

rm(list = ls())

gc()

library(dplyr)

library(caret)

library(readr)

library(tensorflow)

library(keras)

library(openxlsx)

tensorflow::set_random_seed(123)

data <- read_csv("D:/felash10/USB Drive/phd/payanname phd/thesis/final.simulation/TD/models in average progression_td/data_average_ecc.csv")

nvisit <- 11

neye <- 12000

num_features <- 52

start_col_TD <- 5

start_col_PD <- start_col_TD + num_features

data$eye <- rep(1:neye, each = nvisit)

normalize <- function(x) {

(x - min(x, na.rm = TRUE)) / (max(x, na.rm = TRUE) - min(x, na.rm = TRUE))

}

normalized_TD_columns <- paste0("TD_", 1:num_features, "_norm")

normalized_PD_columns <- paste0("PD_", 1:num_features, "_norm")

data[normalized_TD_columns] <- lapply(data[start_col_TD:(start_col_TD + num_features - 1)], normalize)

data[normalized_PD_columns] <- lapply(data[start_col_PD:(start_col_PD + num_features - 1)], normalize)

set.seed(123)

test_indices <- sample(1:neye, size = 0.15 * neye)

train_indices <- setdiff(1:neye, test_indices)

inputs_list <- list()

outputs_list <- list()

create_windows <- function(eye_data, is_test = FALSE) {

input_list <- list()

output_list <- list()

if (is_test) {

input <- as.matrix(eye_data[2:6, c(normalized_TD_columns, normalized_PD_columns)])

output <- as.matrix(eye_data[7, normalized_TD_columns])

input_list[[1]] <- input

output_list[[1]] <- output

}else {

for (i in 1:2) {

input <- as.matrix(eye_data[i:(i+4), c(normalized_TD_columns, normalized_PD_columns)])

output <- as.matrix(eye_data[i+5, normalized_TD_columns])

input_list[[i]] <- input

output_list[[i]] <- output

{

{

return(list(inputs = input_list, outputs = output_list))

{

for (eye_id in 1:neye) {

eye_data <- data[data$eye == eye_id, ]

windows <- create_windows(eye_data, is_test = (eye_id %in% test_indices))

inputs_list[[eye_id]] <- windows$inputs

outputs_list[[eye_id]] <- windows$outputs

{

train_inputs <- inputs_list[train_indices]

train_outputs <- outputs_list[train_indices]

test_inputs <- inputs_list[test_indices]

test_outputs <- outputs_list[test_indices]

x_train <- as.array(train_inputs, dim = c(length(train_indices), 2, 5, num_features * 2))

y_train <- as.array(train_outputs, dim = c(length(train_indices), 2, num_features))

x_test <- as.array(test_inputs, dim = c(length(test_indices), 1, 5, num_features * 2))

y_test <- as.array(test_outputs, dim = c(length(test_indices), 1, num_features))

x_train_tensor <- as_tensor(unlist(x_train),shape=c(10200,2,5,104))

y_train_tensor <- as_tensor(unlist(y_train),shape=c(10200,2,52))

x_test_tensor <- as_tensor(unlist(x_test),shape=c(1800,1,5,104))

y_test_tensor <- as_tensor(unlist(y_test),shape=c(1800,1,52))

build_model <- function() {

input <- layer_input(shape = c(NA, 5, 104))

model <- input %>%

time_distributed(layer_lstm(units = 64, return_sequences = FALSE)) %>%

layer_lstm(units = 60, return_sequences = TRUE) %>%

layer_dropout(rate = 0.1) %>%

time_distributed(layer_dense(units = 52, activation = "linear",

kernel_regularizer = regularizer_l2(0.0001)))

model_final <- keras_model(inputs = input, outputs = model)

model_final %>% compile(

loss = "mean_squared_error",

optimizer = optimizer_adam(learning_rate = 0.0001),

metrics = c("mean_squared_error", "mean_absolute_error")

(

return(model_final)

{

model <- build_model()

early_stopping <- callback_early_stopping(

monitor = "val_loss",

patience = 4,

min_delta = 0.001,

restore_best_weights = TRUE

(

history <- model %>% fit(

x_train_tensor, y_train_tensor,

validation_split = 0.1,

epochs = 100,

batch_size = 512,

verbose = 2,

callbacks = list(early_stopping)

(

y_test_pred <- model %>% predict(x_test_tensor)

valuation_train <- model %>% evaluate(x_train_tensor, y_train_tensor, verbose = 0)

evaluation_test <- model %>% evaluate(x_test_tensor, y_test_tensor, verbose = 0)

cat("Training MSE (TD):", valuation_train[[2]], "\n")

cat("Testing MSE (TD):", evaluation_test[[2]], "\n")

library(dplyr)

library(Metrics)

y_true_array <- as.array(y_test_tensor)

y_pred_array <- as.array(y_test_pred)

y_true_2th <- y_true_array[, 1, ] # dim: num_eyes x 52

y_pred_2th <- y_pred_array[, 1, ]

denorm_true2 <- matrix(NA, nrow = nrow(y_true_2th), ncol = num_features)

denorm_pred2 <- matrix(NA, nrow = nrow(y_pred_2th), ncol = num_features)

for (i in 1:num_features) {

col_name <- paste0("TD_", i)

min_val <- min(data[[col_name]], na.rm = TRUE)

max_val <- max(data[[col_name]], na.rm = TRUE)

denorm_true2[, i] <- y_true_2th[, i] * (max_val - min_val) + min_val

denorm_pred2[, i] <- y_pred_2th[, i] * (max_val - min_val) + min_val

{

rmse_per_eye2 <- sqrt(rowMeans((denorm_pred2 - denorm_true2)^2, na.rm = TRUE))

data_rmse_window2 <- data.frame(

eye_id = test_indices,

rmse = rmse_per_eye2

(

head(data_rmse_window2)

mae_per_feature2 <- colMeans(abs(denorm_pred2 - denorm_true2), na.rm = TRUE)

data_mae_window2 <- data.frame(

location = paste0("TD_", 1:num_features),

MAE = mae_per_feature2

(

head(data_mae_window2)

write_csv(data_rmse_window2, "D:/average_rmse_LSTM-6.csv")

write_csv(data_mae_window2, "D:/average_mae_LSTM-6.csv")

mae_matrix_window2 <- abs(denorm_pred2 - denorm_true2) #

mae_per_eye_loc <- as.data.frame(mae_matrix_window2)

colnames(mae_per_eye_loc) <- paste0("TD_", 1:num_features)

rownames(mae_per_eye_loc) <- test_indices

write_csv(mae_per_eye_loc, "D:/average_mae_per_eye_loc_lstm_6.csv")

Supplement Material- LSTM Model Code

S2. LSTM Model Implementation for Examination 8 Under the Moderate Progression Rate Scenario

rm(list = ls())

gc()

library(dplyr)

library(caret)

library(readr)

library(tensorflow)

library(keras)

library(openxlsx)

tensorflow::set_random_seed(123)

data <- read_csv("D:/felash10/USB Drive/phd/payanname phd/thesis/final.simulation/TD/models in average progression_td/data_average_ecc.csv")

nvisit <- 11

neye <- 12000

num_features <- 52

start_col_TD <- 5

start_col_PD <- start_col_TD + num_features

data$eye <- rep(1:neye, each = nvisit)

normalize <- function(x) {

(x - min(x, na.rm = TRUE)) / (max(x, na.rm = TRUE) - min(x, na.rm = TRUE))

}

normalized_TD_columns <- paste0("TD_", 1:num_features, "_norm")

normalized_PD_columns <- paste0("PD_", 1:num_features, "_norm")

data[normalized_TD_columns] <- lapply(data[start_col_TD:(start_col_TD + num_features - 1)], normalize)

data[normalized_PD_columns] <- lapply(data[start_col_PD:(start_col_PD + num_features - 1)], normalize)

set.seed(123)

test_indices <- sample(1:neye, size = 0.15 * neye)

train_indices <- setdiff(1:neye, test_indices)

inputs_list <- list()

outputs_list <- list()

create_windows <- function(eye_data, is_test = FALSE) {

input_list <- list()

output_list <- list()

if (is_test) {

input <- as.matrix(eye_data[3:7, c(normalized_TD_columns, normalized_PD_columns)])

output <- as.matrix(eye_data[8, normalized_TD_columns])

input_list[[1]] <- input

output_list[[1]] <- output

}else {

for (i in 1:3) {

input <- as.matrix(eye_data[i:(i+4), c(normalized_TD_columns, normalized_PD_columns)])

output <- as.matrix(eye_data[i+5, normalized_TD_columns])

input_list[[i]] <- input

output_list[[i]] <- output

{

{

return(list(inputs = input_list, outputs = output_list))

{

for (eye_id in 1:neye) {

eye_data <- data[data$eye == eye_id, ]

windows <- create_windows(eye_data, is_test = (eye_id %in% test_indices))

inputs_list[[eye_id]] <- windows$inputs

outputs_list[[eye_id]] <- windows$outputs

{

train_inputs <- inputs_list[train_indices]

train_outputs <- outputs_list[train_indices]

test_inputs <- inputs_list[test_indices]

test_outputs <- outputs_list[test_indices]

x_train <- as.array(train_inputs, dim = c(length(train_indices), 3, 5, num_features * 2))

y_train <- as.array(train_outputs, dim = c(length(train_indices), 3, num_features))

x_test <- as.array(test_inputs, dim = c(length(test_indices), 1, 5, num_features * 2))

y_test <- as.array(test_outputs, dim = c(length(test_indices), 1, num_features))

x_train_tensor <- as_tensor(unlist(x_train),shape=c(10200,3,5,104))

y_train_tensor <- as_tensor(unlist(y_train),shape=c(10200,3,52))

x_test_tensor <- as_tensor(unlist(x_test),shape=c(1800,1,5,104))

y_test_tensor <- as_tensor(unlist(y_test),shape=c(1800,1,52))

build_model <- function() {

input <- layer_input(shape = c(NA, 5, 104))

model <- input %>%

time_distributed(layer_lstm(units = 64, return_sequences = FALSE)) %>%

layer_lstm(units = 70, return_sequences = TRUE) %>%

layer_dropout(rate = 0.1) %>%

time_distributed(layer_dense(units = 52, activation = "linear",

kernel_regularizer = regularizer_l2(0.0001)))

model_final <- keras_model(inputs = input, outputs = model)

model_final %>% compile(

loss = "mean_squared_error",

optimizer = optimizer_adam(learning_rate = 0.0001),

metrics = c("mean_squared_error", "mean_absolute_error")

(

return(model_final)

{

model <- build_model()

early_stopping <- callback_early_stopping(

monitor = "val_loss",

patience = 4,

min_delta = 0.001,

restore_best_weights = TRUE

(

history <- model %>% fit(

x_train_tensor, y_train_tensor,

validation_split = 0.1,

epochs = 100,

batch_size = 512,

verbose = 2,

callbacks = list(early_stopping)

)

y_test_pred <- model %>% predict(x_test_tensor)

valuation_train <- model %>% evaluate(x_train_tensor, y_train_tensor, verbose = 0)

evaluation_test <- model %>% evaluate(x_test_tensor, y_test_tensor, verbose = 0)

cat("Training MSE (TD):", valuation_train[[2]], "\n")

cat("Testing MSE (TD):", evaluation_test[[2]], "\n")

library(dplyr)

library(Metrics)

y_true_array <- as.array(y_test_tensor)

y_pred_array <- as.array(y_test_pred)

y_true_3th <- y_true_array[, 1, ] # dim: num_eyes x 52

y_pred_3th <- y_pred_array[, 1, ]

denorm_true3 <- matrix(NA, nrow = nrow(y_true_3th), ncol = num_features)

denorm_pred3 <- matrix(NA, nrow = nrow(y_pred_3th), ncol = num_features)

for (i in 1:num_features) {

col_name <- paste0("TD_", i)

min_val <- min(data[[col_name]], na.rm = TRUE)

max_val <- max(data[[col_name]], na.rm = TRUE)

denorm_true3[, i] <- y_true_3th[, i] * (max_val - min_val) + min_val

denorm_pred3[, i] <- y_pred_3th[, i] * (max_val - min_val) + min_val

{

rmse_per_eye3 <- sqrt(rowMeans((denorm_pred3 - denorm_true3)^2, na.rm = TRUE))

data_rmse_window3 <- data.frame(

eye_id = test_indices,

rmse = rmse_per_eye3

(

head(data_rmse_window3)

mae_per_feature3 <- colMeans(abs(denorm_pred3 - denorm_true3), na.rm = TRUE)

data_mae_window3 <- data.frame(

location = paste0("TD_", 1:num_features),

MAE = mae_per_feature3

)

head(data_mae_window3)

write_csv(data_rmse_window3, "D:/average_rmse_LSTM-7.csv")

write_csv(data_mae_window3, "D:/average_mae_LSTM-7.csv")

mae_matrix_window3 <- abs(denorm_pred3 - denorm_true3)

mae_per_eye_loc <- as.data.frame(mae_matrix_window3)

colnames(mae_per_eye_loc) <- paste0("TD_", 1:num_features)

rownames(mae_per_eye_loc) <- test_indices

head(mae_per_eye_loc[, 1:5])

write_csv(mae_per_eye_loc, "D:/average_mae_per_eye_loc_lstm_7.csv")

Supplement Material- LSTM Model Code

S2. LSTM Model Implementation for Examination 9 Under the Moderate Progression Rate Scenario

rm(list = ls())

gc()

library(dplyr)

library(caret)

library(readr)

library(tensorflow)

library(keras)

library(openxlsx)

tensorflow::set_random_seed(123)

data <- read_csv("D:/felash10/USB Drive/phd/payanname phd/thesis/final.simulation/TD/models in average progression_td/data_average_ecc.csv")

nvisit <- 11

neye <- 12000

num_features <- 52

start_col_TD <- 5

start_col_PD <- start_col_TD + num_features

data$eye <- rep(1:neye, each = nvisit)

normalize <- function(x) {

(x - min(x, na.rm = TRUE)) / (max(x, na.rm = TRUE) - min(x, na.rm = TRUE))

}

normalized_TD_columns <- paste0("TD_", 1:num_features, "_norm")

normalized_PD_columns <- paste0("PD_", 1:num_features, "_norm")

data[normalized_TD_columns] <- lapply(data[start_col_TD:(start_col_TD + num_features - 1)], normalize)

data[normalized_PD_columns] <- lapply(data[start_col_PD:(start_col_PD + num_features - 1)], normalize)

set.seed(123)

test_indices <- sample(1:neye, size = 0.15 * neye)

train_indices <- setdiff(1:neye, test_indices)

inputs_list <- list()

outputs_list <- list()

create_windows <- function(eye_data, is_test = FALSE) {

input_list <- list()

output_list <- list()

input <- as.matrix(eye_data[4:8, c(normalized_TD_columns, normalized_PD_columns)])

output <- as.matrix(eye_data[9, normalized_TD_columns])

input_list[[1]] <- input

output_list[[1]] <- output

else {{

for (i in 1:4) {

input <- as.matrix(eye_data[i:(i+4), c(normalized_TD_columns, normalized_PD_columns)])

output <- as.matrix(eye_data[i+5, normalized_TD_columns])

input_list[[i]] <- input

output_list[[i]] <- output

}

}

return(list(inputs = input_list, outputs = output_list))

}

for (eye_id in 1:neye) {

eye_data <- data[data$eye == eye_id, ]

windows <- create_windows(eye_data, is_test = (eye_id %in% test_indices))

inputs_list[[eye_id]] <- windows$inputs

outputs_list[[eye_id]] <- windows$outputs

}

train_inputs <- inputs_list[train_indices]

train_outputs <- outputs_list[train_indices]

test_inputs <- inputs_list[test_indices]

test_outputs <- outputs_list[test_indices]

x_train <- as.array(train_inputs, dim = c(length(train_indices), 4, 5, num_features * 2))

y_train <- as.array(train_outputs, dim = c(length(train_indices), 4, num_features))

x_test <- as.array(test_inputs, dim = c(length(test_indices), 1, 5, num_features * 2))

y_test <- as.array(test_outputs, dim = c(length(test_indices), 1, num_features))

x_train_tensor <- as_tensor(unlist(x_train),shape=c(10200,4,5,104))

y_train_tensor <- as_tensor(unlist(y_train),shape=c(10200,4,52))

x_test_tensor <- as_tensor(unlist(x_test),shape=c(1800,1,5,104))

y_test_tensor <- as_tensor(unlist(y_test),shape=c(1800,1,52))

build_model <- function() {

input <- layer_input(shape = c(NA, 5, 104))

model <- input %>%

time_distributed(layer_lstm(units = 64, return_sequences = FALSE)) %>%

layer_lstm(units = 85, return_sequences = TRUE) %>%

layer_dropout(rate = 0.1) %>%

time_distributed(layer_dense(units = 52, activation = "linear",

kernel_regularizer = regularizer_l2(0.0001)))

model_final <- keras_model(inputs = input, outputs = model)

model_final %>% compile(

loss = "mean_squared_error",

optimizer = optimizer_adam(learning_rate = 0.0001),

metrics = c("mean_squared_error", "mean_absolute_error")

)

return(model_final)

}

model <- build_model()

early_stopping <- callback_early_stopping(

monitor = "val_loss",

patience = 4,

min_delta = 0.001,

restore_best_weights = TRUE

)

history <- model %>% fit(

x_train_tensor, y_train_tensor,

validation_split = 0.1,

epochs = 100,

batch_size = 512,

verbose = 2,

callbacks = list(early_stopping)

)

y_test_pred <- model %>% predict(x_test_tensor)

valuation_train <- model %>% evaluate(x_train_tensor, y_train_tensor, verbose = 0)

evaluation_test <- model %>% evaluate(x_test_tensor, y_test_tensor, verbose = 0)

cat("Training MSE (TD):", valuation_train[[2]], "\n")

cat("Testing MSE (TD):", evaluation_test[[2]], "\n")

library(dplyr)

library(Metrics)

y_true_array <- as.array(y_test_tensor)

y_pred_array <- as.array(y_test_pred)

\# پنجره ششم برای هر چشم (یعنی index = 4)

y_true_4th <- y_true_array[, 1, ] # dim: num_eyes x 52

y_pred_4th <- y_pred_array[, 1, ]

denorm_true4 <- matrix(NA, nrow = nrow(y_true_4th), ncol = num_features)

denorm_pred4 <- matrix(NA, nrow = nrow(y_pred_4th), ncol = num_features)

for (i in 1:num_features) {

col_name <- paste0("TD_", i)

min_val <- min(data[[col_name]], na.rm = TRUE)

max_val <- max(data[[col_name]], na.rm = TRUE)

denorm_true4[, i] <- y_true_4th[, i] * (max_val - min_val) + min_val

denorm_pred4[, i] <- y_pred_4th[, i] * (max_val - min_val) + min_val

}

rmse_per_eye4 <- sqrt(rowMeans((denorm_pred4 - denorm_true4)^2, na.rm = TRUE))

data_rmse_window4 <- data.frame(

eye_id = test_indices,

rmse = rmse_per_eye4

)

head(data_rmse_window4)

mae_per_feature4 <- colMeans(abs(denorm_pred4 - denorm_true4), na.rm = TRUE)

data_mae_window4 <- data.frame(

location = paste0("TD_", 1:num_features),

MAE = mae_per_feature4

)

head(data_mae_window4)

write_csv(data_rmse_window4, "D:/average_rmse_LSTM-8.csv")

write_csv(data_mae_window4, "D:/average_mae_LSTM-8.csv")

mae_matrix_window4 <- abs(denorm_pred4 - denorm_true4)

mae_per_eye_loc <- as.data.frame(mae_matrix_window4)

colnames(mae_per_eye_loc) <- paste0("TD_", 1:num_features)

rownames(mae_per_eye_loc) <- test_indices

dim(mae_per_eye_loc)

head(mae_per_eye_loc[, 1:5])

write_csv(mae_per_eye_loc, "D:/average_mae_per_eye_loc_lstm_8.csv")

Supplement Material- LSTM Model Code

S2. LSTM Model Implementation for Examination 10 Under the Moderate Progression Rate Scenario

rm(list = ls())

gc()

library(dplyr)

library(caret)

library(readr)

library(tensorflow)

library(keras)

library(openxlsx)

tensorflow::set_random_seed(123)

data <- read_csv("D:/felash10/USB Drive/phd/payanname phd/thesis/final.simulation/TD/models in average progression_td/data_average_ecc.csv")

nvisit <- 11

neye <- 12000

num_features <- 52

start_col_TD <- 5

start_col_PD <- start_col_TD + num_features

data$eye <- rep(1:neye, each = nvisit)

normalize <- function(x) {

(x - min(x, na.rm = TRUE)) / (max(x, na.rm = TRUE) - min(x, na.rm = TRUE))

}

normalized_TD_columns <- paste0("TD_", 1:num_features, "_norm")

normalized_PD_columns <- paste0("PD_", 1:num_features, "_norm")

data[normalized_TD_columns] <- lapply(data[start_col_TD:(start_col_TD + num_features - 1)], normalize)

data[normalized_PD_columns] <- lapply(data[start_col_PD:(start_col_PD + num_features - 1)], normalize)

set.seed(123)

test_indices <- sample(1:neye, size = 0.15 * neye)

train_indices <- setdiff(1:neye, test_indices)

inputs_list <- list()

outputs_list <- list()

create_windows <- function(eye_data, is_test = FALSE) {

input_list <- list()

output_list <- list()

if (is_test) {

input <- as.matrix(eye_data[5:9, c(normalized_TD_columns, normalized_PD_columns)])

output <- as.matrix(eye_data[10, normalized_TD_columns])

input_list[[1]] <- input

output_list[[1]] <- output

}else {

for (i in 1:5) {

input <- as.matrix(eye_data[i:(i+4), c(normalized_TD_columns, normalized_PD_columns)])

output <- as.matrix(eye_data[i+5, normalized_TD_columns])

input_list[[i]] <- input

output_list[[i]] <- output

}

{

return(list(inputs = input_list, outputs = output_list))

{

for (eye_id in 1:neye) {

eye_data <- data[data$eye == eye_id, ]

windows <- create_windows(eye_data, is_test = (eye_id %in% test_indices))

inputs_list[[eye_id]] <- windows$inputs

outputs_list[[eye_id]] <- windows$outputs

{

train_inputs <- inputs_list[train_indices]

train_outputs <- outputs_list[train_indices]

test_inputs <- inputs_list[test_indices]

test_outputs <- outputs_list[test_indices]

x_train <- as.array(train_inputs, dim = c(length(train_indices), 5, 5, num_features * 2))

y_train <- as.array(train_outputs, dim = c(length(train_indices), 5, num_features))

x_test <- as.array(test_inputs, dim = c(length(test_indices), 1, 5, num_features * 2))

y_test <- as.array(test_outputs, dim = c(length(test_indices), 1, num_features))

x_train_tensor <- as_tensor(unlist(x_train),shape=c(10200,5,5,104))

y_train_tensor <- as_tensor(unlist(y_train),shape=c(10200,5,52))

x_test_tensor <- as_tensor(unlist(x_test),shape=c(1800,1,5,104))

y_test_tensor <- as_tensor(unlist(y_test),shape=c(1800,1,52))

build_model <- function() {

input <- layer_input(shape = c(NA, 5, 104))

model <- input %>%

time_distributed(layer_lstm(units = 64, return_sequences = FALSE)) %>%

layer_lstm(units = 100, return_sequences = TRUE) %>%

layer_dropout(rate = 0.1) %>%

time_distributed(layer_dense(units = 52, activation = "linear",

kernel_regularizer = regularizer_l2(0.0001)))

model_final <- keras_model(inputs = input, outputs = model)

model_final %>% compile(

loss = "mean_squared_error",

optimizer = optimizer_adam(learning_rate = 0.0001),

metrics = c("mean_squared_error", "mean_absolute_error")

(

return(model_final)

{

model <- build_model()

early_stopping <- callback_early_stopping(

monitor = "val_loss",

patience = 4,

min_delta = 0.001,

restore_best_weights = TRUE

(

history <- model %>% fit(

x_train_tensor, y_train_tensor,

validation_split = 0.1,

epochs = 100,

batch_size = 512,

verbose = 2,

callbacks = list(early_stopping)

(

y_test_pred <- model %>% predict(x_test_tensor)

valuation_train <- model %>% evaluate(x_train_tensor, y_train_tensor, verbose = 0)

evaluation_test <- model %>% evaluate(x_test_tensor, y_test_tensor, verbose = 0)

cat("Training MSE (TD):", valuation_train[[2]], "\n")

cat("Testing MSE (TD):", evaluation_test[[2]], "\n")

library(dplyr)

library(Metrics)

y_true_array <- as.array(y_test_tensor)

y_pred_array <- as.array(y_test_pred)

y_true_5th <- y_true_array[, 1, ]

y_pred_5th <- y_pred_array[, 1, ]

denorm_true5 <- matrix(NA, nrow = nrow(y_true_5th), ncol = num_features)

denorm_pred5 <- matrix(NA, nrow = nrow(y_pred_5th), ncol = num_features)

for (i in 1:num_features) {

col_name <- paste0("TD_", i)

min_val <- min(data[[col_name]], na.rm = TRUE)

max_val <- max(data[[col_name]], na.rm = TRUE)

denorm_true5[, i] <- y_true_5th[, i] * (max_val - min_val) + min_val

denorm_pred5[, i] <- y_pred_5th[, i] * (max_val - min_val) + min_val

{

rmse_per_eye5 <- sqrt(rowMeans((denorm_pred5 - denorm_true5)^2, na.rm = TRUE))

data_rmse_window5 <- data.frame(

eye_id = test_indices,

rmse = rmse_per_eye5

(

head(data_rmse_window5)

mae_per_feature5 <- colMeans(abs(denorm_pred5 - denorm_true5), na.rm = TRUE)

data_mae_window5 <- data.frame(

location = paste0("TD_", 1:num_features),

MAE = mae_per_feature5

(

head(data_mae_window5)

write_csv(data_rmse_window5, "D:/average_rmse_LSTM-9.csv")

write_csv(data_mae_window5, "D:/average_mae_LSTM-9.csv")

mae_matrix_window5 <- abs(denorm_pred5 - denorm_true5)

mae_per_eye_loc <- as.data.frame(mae_matrix_window5)

colnames(mae_per_eye_loc) <- paste0("TD_", 1:num_features)

rownames(mae_per_eye_loc) <- test_indices

write_csv(mae_per_eye_loc, "D:/average_mae_per_eye_loc_lstm_9.csv")

Supplement Material- LSTM Model Code

S2. LSTM Model Implementation for Examination 11 Under the Moderate Progression Rate Scenario

rm(list = ls())

gc()

library(dplyr)

library(caret)

library(readr)

library(tensorflow)

library(keras)

library(openxlsx)

tensorflow::set_random_seed(123)

data <- read_csv("D:/felash10/USB Drive/phd/payanname phd/thesis/final.simulation/TD/models in average progression_td/data_average_ecc.csv")

nvisit <- 11

neye <- 12000

num_features <- 52

start_col_TD <- 5

start_col_PD <- start_col_TD + num_features

data$eye <- rep(1:neye, each = nvisit)

normalize <- function(x) {

(x - min(x, na.rm = TRUE)) / (max(x, na.rm = TRUE) - min(x, na.rm = TRUE))

{

normalized_TD_columns <- paste0("TD_", 1:num_features, "_norm")

normalized_PD_columns <- paste0("PD_", 1:num_features, "_norm")

data[normalized_TD_columns] <- lapply(data[start_col_TD:(start_col_TD + num_features - 1)], normalize)

data[normalized_PD_columns] <- lapply(data[start_col_PD:(start_col_PD + num_features - 1)], normalize)

set.seed(123)

test_indices <- sample(1:neye, size = 0.15 * neye)

train_indices <- setdiff(1:neye, test_indices)

inputs_list <- list()

outputs_list <- list()

create_windows <- function(eye_data, is_test = FALSE) {

input_list <- list()

output_list <- list()

if (is_test) {

input <- as.matrix(eye_data[6:10, c(normalized_TD_columns, normalized_PD_columns)])

output <- as.matrix(eye_data[11, normalized_TD_columns])

input_list[[1]] <- input

output_list[[1]] <- output

}else {

for (i in 1:6) {

input <- as.matrix(eye_data[i:(i+4), c(normalized_TD_columns, normalized_PD_columns)])

output <- as.matrix(eye_data[i+5, normalized_TD_columns])

input_list[[i]] <- input

output_list[[i]] <- output

{

{

return(list(inputs = input_list, outputs = output_list))

{

for (eye_id in 1:neye) {

eye_data <- data[data$eye == eye_id, ]

windows <- create_windows(eye_data, is_test = (eye_id %in% test_indices))

inputs_list[[eye_id]] <- windows$inputs

outputs_list[[eye_id]] <- windows$outputs

{

train_inputs <- inputs_list[train_indices]

train_outputs <- outputs_list[train_indices]

test_inputs <- inputs_list[test_indices]

test_outputs <- outputs_list[test_indices]

x_train <- as.array(train_inputs, dim = c(length(train_indices), 6, 5, num_features * 2))

y_train <- as.array(train_outputs, dim = c(length(train_indices), 6, num_features))

x_test <- as.array(test_inputs, dim = c(length(test_indices), 1, 5, num_features * 2))

y_test <- as.array(test_outputs, dim = c(length(test_indices), 1, num_features))

x_train_tensor <- as_tensor(unlist(x_train),shape=c(10200,6,5,104))

y_train_tensor <- as_tensor(unlist(y_train),shape=c(10200,6,52))

x_test_tensor <- as_tensor(unlist(x_test),shape=c(1800,1,5,104))

y_test_tensor <- as_tensor(unlist(y_test),shape=c(1800,1,52))

build_model <- function() {

input <- layer_input(shape = c(NA, 5, 104))

model <- input %>%

time_distributed(layer_lstm(units = 64, return_sequences = FALSE)) %>%

layer_lstm(units = 120, return_sequences = TRUE) %>%

layer_dropout(rate = 0.1) %>%

time_distributed(layer_dense(units = 52, activation = "linear",

kernel_regularizer = regularizer_l2(0.0001)))

model_final <- keras_model(inputs = input, outputs = model)

model_final %>% compile(

loss = "mean_squared_error",

optimizer = optimizer_adam(learning_rate = 0.0001),

metrics = c("mean_squared_error", "mean_absolute_error")

(

return(model_final)

{

model <- build_model()

early_stopping <- callback_early_stopping(

monitor = "val_loss",

patience = 4,

min_delta = 0.001,

restore_best_weights = TRUE

(

history <- model %>% fit(

x_train_tensor, y_train_tensor,

validation_split = 0.1,

epochs = 100,

batch_size = 512,

verbose = 2,

callbacks = list(early_stopping)

(

y_test_pred <- model %>% predict(x_test_tensor)

valuation_train <- model %>% evaluate(x_train_tensor, y_train_tensor, verbose = 0)

evaluation_test <- model %>% evaluate(x_test_tensor, y_test_tensor, verbose = 0)

cat("Training MSE (TD):", valuation_train[[2]], "\n")

cat("Testing MSE (TD):", evaluation_test[[2]], "\n")

library(dplyr)

library(Metrics)

y_true_array <- as.array(y_test_tensor)

y_pred_array <- as.array(y_test_pred)

y_true_6th <- y_true_array[, 1, ] # dim: num_eyes x 52

y_pred_6th <- y_pred_array[, 1, ]

denorm_true6 <- matrix(NA, nrow = nrow(y_true_6th), ncol = num_features)

denorm_pred6 <- matrix(NA, nrow = nrow(y_pred_6th), ncol = num_features)

for (i in 1:num_features) {

col_name <- paste0("TD_", i)

min_val <- min(data[[col_name]], na.rm = TRUE)

max_val <- max(data[[col_name]], na.rm = TRUE)

denorm_true6[, i] <- y_true_6th[, i] * (max_val - min_val) + min_val

denorm_pred6[, i] <- y_pred_6th[, i] * (max_val - min_val) + min_val

{

rmse_per_eye6 <- sqrt(rowMeans((denorm_pred6 - denorm_true6)^2, na.rm = TRUE))

data_rmse_window6 <- data.frame(

eye_id = test_indices,

rmse = rmse_per_eye6

(

head(data_rmse_window6)

mae_per_feature6 <- colMeans(abs(denorm_pred6 - denorm_true6), na.rm = TRUE)

data_mae_window6 <- data.frame(

location = paste0("TD_", 1:num_features),

MAE = mae_per_feature6

)

head(data_mae_window6)

write_csv(data_rmse_window6, "D:/average_rmse_LSTM-10.csv")

write_csv(data_mae_window6, "D:/average_mae_LSTM-10.csv")

mae_matrix_window6 <- abs(denorm_pred6 - denorm_true6)

mae_per_eye_loc <- as.data.frame(mae_matrix_window6)

colnames(mae_per_eye_loc) <- paste0("TD_", 1:num_features)

rownames(mae_per_eye_loc) <- test_indices

write_csv(mae_per_eye_loc, "D:/average_mae_per_eye_loc_lstm_10.csv")

Supplement Material- LSTM Model Code

S2. LSTM Model Implementation for Examination 6 Under the Fast Progression Rate Scenario

rm(list = ls())

gc()

library(dplyr)

library(caret)

library(readr)

library(tensorflow)

library(keras)

library(openxlsx)

tensorflow::set_random_seed(123)

data <- read_csv("D:/felash10/USB Drive/phd/payanname phd/thesis/final.simulation/TD/models in quick progression_td/data_quick_ecc.csv")

nvisit <- 11

neye <- 12000

num_features <- 52

start_col_TD <- 5

start_col_PD <- start_col_TD + num_features

data$eye <- rep(1:neye, each = nvisit)

normalize <- function(x) {

(x - min(x, na.rm = TRUE)) / (max(x, na.rm = TRUE) - min(x, na.rm = TRUE))

}

normalized_TD_columns <- paste0("TD_", 1:num_features, "_norm")

normalized_PD_columns <- paste0("PD_", 1:num_features, "_norm")

data[normalized_TD_columns] <- lapply(data[start_col_TD:(start_col_TD + num_features - 1)], normalize)

data[normalized_PD_columns] <- lapply(data[start_col_PD:(start_col_PD + num_features - 1)], normalize)

set.seed(123)

test_indices <- sample(1:neye, size = 0.15 * neye)

train_indices <- setdiff(1:neye, test_indices)

inputs_list <- list()

outputs_list <- list()

create_windows <- function(eye_data, is_test = FALSE) {

input_list <- list()

output_list <- list()

if (is_test) {

input <- as.matrix(eye_data[1:5, c(normalized_TD_columns, normalized_PD_columns)])

output <- as.matrix(eye_data[6, normalized_TD_columns])

input_list[[1]] <- input

output_list[[1]] <- output

}else {

for (i in 1:1) {

input <- as.matrix(eye_data[i:(i+4), c(normalized_TD_columns, normalized_PD_columns)])

output <- as.matrix(eye_data[i+5, normalized_TD_columns])

input_list[[i]] <- input

output_list[[i]] <- output

{

{

return(list(inputs = input_list, outputs = output_list))

{

for (eye_id in 1:neye) {

eye_data <- data[data$eye == eye_id, ]

windows <- create_windows(eye_data, is_test = (eye_id %in% test_indices))

inputs_list[[eye_id]] <- windows$inputs

outputs_list[[eye_id]] <- windows$outputs

{

train_inputs <- inputs_list[train_indices]

train_outputs <- outputs_list[train_indices]

test_inputs <- inputs_list[test_indices]

test_outputs <- outputs_list[test_indices]

x_train <- as.array(train_inputs, dim = c(length(train_indices), 1, 5, num_features * 2))

y_train <- as.array(train_outputs, dim = c(length(train_indices), 1, num_features))

x_test <- as.array(test_inputs, dim = c(length(test_indices), 1, 5, num_features * 2))

y_test <- as.array(test_outputs, dim = c(length(test_indices), 1, num_features))

x_train_tensor <- as_tensor(unlist(x_train),shape=c(10200,1,5,104))

y_train_tensor <- as_tensor(unlist(y_train),shape=c(10200,1,52))

x_test_tensor <- as_tensor(unlist(x_test),shape=c(1800,1,5,104))

y_test_tensor <- as_tensor(unlist(y_test),shape=c(1800,1,52))

build_model <- function() {

input <- layer_input(shape = c(NA, 5, 104))

model <- input %>%

time_distributed(layer_lstm(units = 64, return_sequences = FALSE)) %>%

layer_lstm(units = 60, return_sequences = TRUE) %>%

layer_dropout(rate = 0.1) %>%

time_distributed(layer_dense(units = 52, activation = "linear",

kernel_regularizer = regularizer_l2(0.0001)))

model_final <- keras_model(inputs = input, outputs = model)

model_final %>% compile(

loss = "mean_squared_error",

optimizer = optimizer_adam(learning_rate = 0.0001),

metrics = c("mean_squared_error", "mean_absolute_error")

(

return(model_final)

{

model <- build_model()

early_stopping <- callback_early_stopping(

monitor = "val_loss",

patience = 4,

min_delta = 0.001,

restore_best_weights = TRUE

(

history <- model %>% fit(

x_train_tensor, y_train_tensor,

validation_split = 0.1,

epochs = 100,

batch_size = 512,

verbose = 2,

callbacks = list(early_stopping)

(

y_test_pred <- model %>% predict(x_test_tensor)

valuation_train <- model %>% evaluate(x_train_tensor, y_train_tensor, verbose = 0)

evaluation_test <- model %>% evaluate(x_test_tensor, y_test_tensor, verbose = 0)

cat("Training MSE (TD):", valuation_train[[2]], "\n")

cat("Testing MSE (TD):", evaluation_test[[2]], "\n")

library(dplyr)

library(Metrics)

y_true_array <- as.array(y_test_tensor)

y_pred_array <- as.array(y_test_pred)

y_true_1th <- y_true_array[, 1, ]

y_pred_1th <- y_pred_array[, 1, ]

denorm_true1 <- matrix(NA, nrow = nrow(y_true_1th), ncol = num_features)

denorm_pred1 <- matrix(NA, nrow = nrow(y_pred_1th), ncol = num_features)

for (i in 1:num_features) {

col_name <- paste0("TD_", i)

min_val <- min(data[[col_name]], na.rm = TRUE)

max_val <- max(data[[col_name]], na.rm = TRUE)

denorm_true1[, i] <- y_true_1th[, i] * (max_val - min_val) + min_val

denorm_pred1[, i] <- y_pred_1th[, i] * (max_val - min_val) + min_val

{

rmse_per_eye1 <- sqrt(rowMeans((denorm_pred1 - denorm_true1)^2, na.rm = TRUE))

data_rmse_window1 <- data.frame(

eye_id = test_indices,

rmse = rmse_per_eye1

(

head(data_rmse_window1)

mae_per_feature1 <- colMeans(abs(denorm_pred1 - denorm_true1), na.rm = TRUE)

data_mae_window1 <- data.frame(

location = paste0("TD_", 1:num_features),

MAE = mae_per_feature1

(

head(data_mae_window1)

write_csv(data_rmse_window1, "D:/quick_rmse_LSTM-5.csv")

write_csv(data_mae_window1, "D:/quick_mae_LSTM-5.csv")

mae_matrix_window1 <- abs(denorm_pred1 - denorm_true1)

mae_per_eye_loc <- as.data.frame(mae_matrix_window1)

colnames(mae_per_eye_loc) <- paste0("TD_", 1:num_features)

rownames(mae_per_eye_loc) <- test_indices

write_csv(mae_per_eye_loc, "D:/quick_mae_per_eye_loc_lstm_5.csv")

Supplement Material- LSTM Model Code

S2. LSTM Model Implementation for Examination 7 Under the Fast Progression Rate Scenario

rm(list = ls())

gc()

library(dplyr)

library(caret)

library(readr)

library(tensorflow)

library(keras)

library(openxlsx)

tensorflow::set_random_seed(123)

data <- read_csv("D:/felash10/USB Drive/phd/payanname phd/thesis/final.simulation/TD/models in quick progression_td/data_quick_ecc.csv")

nvisit <- 11

neye <- 12000

num_features <- 52

start_col_TD <- 5

start_col_PD <- start_col_TD + num_features

data$eye <- rep(1:neye, each = nvisit)

normalize <- function(x) {

(x - min(x, na.rm = TRUE)) / (max(x, na.rm = TRUE) - min(x, na.rm = TRUE))

{

normalized_TD_columns <- paste0("TD_", 1:num_features, "_norm")

normalized_PD_columns <- paste0("PD_", 1:num_features, "_norm")

data[normalized_TD_columns] <- lapply(data[start_col_TD:(start_col_TD + num_features - 1)], normalize)

data[normalized_PD_columns] <- lapply(data[start_col_PD:(start_col_PD + num_features - 1)], normalize)

set.seed(123)

test_indices <- sample(1:neye, size = 0.15 * neye)

train_indices <- setdiff(1:neye, test_indices)

inputs_list <- list()

outputs_list <- list()

create_windows <- function(eye_data, is_test = FALSE) {

input_list <- list()

output_list <- list()

if (is_test) {

input <- as.matrix(eye_data[2:6, c(normalized_TD_columns, normalized_PD_columns)])

output <- as.matrix(eye_data[7, normalized_TD_columns])

input_list[[1]] <- input

output_list[[1]] <- output

else {{

for (i in 1:2) {

input <- as.matrix(eye_data[i:(i+4), c(normalized_TD_columns, normalized_PD_columns)])

output <- as.matrix(eye_data[i+5, normalized_TD_columns])

input_list[[i]] <- input

output_list[[i]] <- output

{

{

return(list(inputs = input_list, outputs = output_list))

{

for (eye_id in 1:neye) {

eye_data <- data[data$eye == eye_id, ]

windows <- create_windows(eye_data, is_test = (eye_id %in% test_indices))

inputs_list[[eye_id]] <- windows$inputs

outputs_list[[eye_id]] <- windows$outputs

{

train_inputs <- inputs_list[train_indices]

train_outputs <- outputs_list[train_indices]

test_inputs <- inputs_list[test_indices]

test_outputs <- outputs_list[test_indices]

x_train <- as.array(train_inputs, dim = c(length(train_indices), 2, 5, num_features * 2))

y_train <- as.array(train_outputs, dim = c(length(train_indices), 2, num_features))

x_test <- as.array(test_inputs, dim = c(length(test_indices), 1, 5, num_features * 2))

y_test <- as.array(test_outputs, dim = c(length(test_indices), 1, num_features))

x_train_tensor <- as_tensor(unlist(x_train),shape=c(10200,2,5,104))

y_train_tensor <- as_tensor(unlist(y_train),shape=c(10200,2,52))

x_test_tensor <- as_tensor(unlist(x_test),shape=c(1800,1,5,104))

y_test_tensor <- as_tensor(unlist(y_test),shape=c(1800,1,52))

build_model <- function() {

input <- layer_input(shape = c(NA, 5, 104))

model <- input %>%

time_distributed(layer_lstm(units = 64, return_sequences = FALSE)) %>%

layer_lstm(units = 70, return_sequences = TRUE) %>%

layer_dropout(rate = 0.1) %>%

time_distributed(layer_dense(units = 52, activation = "linear",

kernel_regularizer = regularizer_l2(0.0001)))

model_final <- keras_model(inputs = input, outputs = model)

model_final %>% compile(

loss = "mean_squared_error",

optimizer = optimizer_adam(learning_rate = 0.0001),

metrics = c("mean_squared_error", "mean_absolute_error")

(

return(model_final)

{

model <- build_model()

early_stopping <- callback_early_stopping(

monitor = "val_loss",

patience = 4,

min_delta = 0.001,

restore_best_weights = TRUE

(

history <- model %>% fit(

x_train_tensor, y_train_tensor,

validation_split = 0.1,

epochs = 100,

batch_size = 512,

verbose = 2,

callbacks = list(early_stopping)

(

y_test_pred <- model %>% predict(x_test_tensor)

valuation_train <- model %>% evaluate(x_train_tensor, y_train_tensor, verbose = 0)

evaluation_test <- model %>% evaluate(x_test_tensor, y_test_tensor, verbose = 0)

cat("Training MSE (TD):", valuation_train[[2]], "\n")

cat("Testing MSE (TD):", evaluation_test[[2]], "\n")

library(dplyr)

library(Metrics)

y_true_array <- as.array(y_test_tensor)

y_pred_array <- as.array(y_test_pred)

y_true_2th <- y_true_array[, 1, ] # dim: num_eyes x 52

y_pred_2th <- y_pred_array[, 1, ]

denorm_true2 <- matrix(NA, nrow = nrow(y_true_2th), ncol = num_features)

denorm_pred2 <- matrix(NA, nrow = nrow(y_pred_2th), ncol = num_features)

for (i in 1:num_features) {

col_name <- paste0("TD_", i)

min_val <- min(data[[col_name]], na.rm = TRUE)

max_val <- max(data[[col_name]], na.rm = TRUE)

denorm_true2[, i] <- y_true_2th[, i] * (max_val - min_val) + min_val

denorm_pred2[, i] <- y_pred_2th[, i] * (max_val - min_val) + min_val

{

rmse_per_eye2 <- sqrt(rowMeans((denorm_pred2 - denorm_true2)^2, na.rm = TRUE))

data_rmse_window2 <- data.frame(

eye_id = test_indices,

rmse = rmse_per_eye2

(

head(data_rmse_window2)

mae_per_feature2 <- colMeans(abs(denorm_pred2 - denorm_true2), na.rm = TRUE)

data_mae_window2 <- data.frame(

location = paste0("TD_", 1:num_features),

MAE = mae_per_feature2

(

head(data_mae_window2)

write_csv(data_rmse_window2, "D:/quick_rmse_LSTM-6.csv")

write_csv(data_mae_window2, "D:/quick_mae_LSTM-6.csv")

mae_matrix_window2 <- abs(denorm_pred2 - denorm_true2)

mae_per_eye_loc <- as.data.frame(mae_matrix_window2)

colnames(mae_per_eye_loc) <- paste0("TD_", 1:num_features)

rownames(mae_per_eye_loc) <- test_indices

dim(mae_per_eye_loc)

head(mae_per_eye_loc[, 1:5])

write_csv(mae_per_eye_loc, "D:/quick_mae_per_eye_loc_lstm_6.csv")

Supplement Material- LSTM Model Code

S2. LSTM Model Implementation for Examination 8 Under the Fast Progression Rate Scenario

rm(list = ls())

gc()

library(dplyr)

library(caret)

library(readr)

library(tensorflow)

library(keras)

library(openxlsx)

tensorflow::set_random_seed(123)

data <- read_csv("D:/felash10/USB Drive/phd/payanname phd/thesis/final.simulation/TD/models in quick progression_td/data_quick_ecc.csv")

nvisit <- 11

neye <- 12000

num_features <- 52

start_col_TD <- 5

start_col_PD <- start_col_TD + num_features

data$eye <- rep(1:neye, each = nvisit)

normalize <- function(x) {

(x - min(x, na.rm = TRUE)) / (max(x, na.rm = TRUE) - min(x, na.rm = TRUE))

}

normalized_TD_columns <- paste0("TD_", 1:num_features, "_norm")

normalized_PD_columns <- paste0("PD_", 1:num_features, "_norm")

data[normalized_TD_columns] <- lapply(data[start_col_TD:(start_col_TD + num_features - 1)], normalize)

data[normalized_PD_columns] <- lapply(data[start_col_PD:(start_col_PD + num_features - 1)], normalize)

set.seed(123)

test_indices <- sample(1:neye, size = 0.15 * neye)

train_indices <- setdiff(1:neye, test_indices)

inputs_list <- list()

outputs_list <- list()

create_windows <- function(eye_data, is_test = FALSE) {

input_list <- list()

output_list <- list()

if (is_test) {

input <- as.matrix(eye_data[3:7, c(normalized_TD_columns, normalized_PD_columns)])

output <- as.matrix(eye_data[8, normalized_TD_columns])

input_list[[1]] <- input

output_list[[1]] <- output

else {{

for (i in 1:3) {

input <- as.matrix(eye_data[i:(i+4), c(normalized_TD_columns, normalized_PD_columns)])

output <- as.matrix(eye_data[i+5, normalized_TD_columns])

input_list[[i]] <- input

output_list[[i]] <- output

}

}

return(list(inputs = input_list, outputs = output_list))

}

for (eye_id in 1:neye) {

eye_data <- data[data$eye == eye_id, ]

windows <- create_windows(eye_data, is_test = (eye_id %in% test_indices))

inputs_list[[eye_id]] <- windows$inputs

outputs_list[[eye_id]] <- windows$outputs

}

train_inputs <- inputs_list[train_indices]

train_outputs <- outputs_list[train_indices]

test_inputs <- inputs_list[test_indices]

test_outputs <- outputs_list[test_indices]

x_train <- as.array(train_inputs, dim = c(length(train_indices), 3, 5, num_features * 2))

y_train <- as.array(train_outputs, dim = c(length(train_indices), 3, num_features))

x_test <- as.array(test_inputs, dim = c(length(test_indices), 1, 5, num_features * 2))

y_test <- as.array(test_outputs, dim = c(length(test_indices), 1, num_features))

x_train_tensor <- as_tensor(unlist(x_train),shape=c(10200,3,5,104))

y_train_tensor <- as_tensor(unlist(y_train),shape=c(10200,3,52))

x_test_tensor <- as_tensor(unlist(x_test),shape=c(1800,1,5,104))

y_test_tensor <- as_tensor(unlist(y_test),shape=c(1800,1,52))

build_model <- function() {

input <- layer_input(shape = c(NA, 5, 104))

model <- input %>%

time_distributed(layer_lstm(units = 64, return_sequences = FALSE)) %>%

layer_lstm(units = 85, return_sequences = TRUE) %>%

layer_dropout(rate = 0.1) %>%

time_distributed(layer_dense(units = 52, activation = "linear",

kernel_regularizer = regularizer_l2(0.0001)))

model_final <- keras_model(inputs = input, outputs = model)

model_final %>% compile(

loss = "mean_squared_error",

optimizer = optimizer_adam(learning_rate = 0.0001),

metrics = c("mean_squared_error", "mean_absolute_error")

)

return(model_final)

}

model <- build_model()

early_stopping <- callback_early_stopping(

monitor = "val_loss",

patience = 4,

min_delta = 0.001,

restore_best_weights = TRUE

)

history <- model %>% fit(

x_train_tensor, y_train_tensor,

validation_split = 0.1,

epochs = 100,

batch_size = 512,

verbose = 2,

callbacks = list(early_stopping)

)

y_test_pred <- model %>% predict(x_test_tensor)

valuation_train <- model %>% evaluate(x_train_tensor, y_train_tensor, verbose = 0)

evaluation_test <- model %>% evaluate(x_test_tensor, y_test_tensor, verbose = 0)

cat("Training MSE (TD):", valuation_train[[2]], "\n")

cat("Testing MSE (TD):", evaluation_test[[2]], "\n")

library(dplyr)

library(Metrics)

y_true_array <- as.array(y_test_tensor)

y_pred_array <- as.array(y_test_pred)

y_true_3th <- y_true_array[, 1, ] # dim: num_eyes x 52

y_pred_3th <- y_pred_array[, 1, ]

denorm_true3 <- matrix(NA, nrow = nrow(y_true_3th), ncol = num_features)

denorm_pred3 <- matrix(NA, nrow = nrow(y_pred_3th), ncol = num_features)

for (i in 1:num_features) {

col_name <- paste0("TD_", i)

min_val <- min(data[[col_name]], na.rm = TRUE)

max_val <- max(data[[col_name]], na.rm = TRUE)

denorm_true3[, i] <- y_true_3th[, i] * (max_val - min_val) + min_val

denorm_pred3[, i] <- y_pred_3th[, i] * (max_val - min_val) + min_val

}

rmse_per_eye3 <- sqrt(rowMeans((denorm_pred3 - denorm_true3)^2, na.rm = TRUE))

data_rmse_window3 <- data.frame(

eye_id = test_indices,

rmse = rmse_per_eye3

)

head(data_rmse_window3)

mae_per_feature3 <- colMeans(abs(denorm_pred3 - denorm_true3), na.rm = TRUE)

data_mae_window3 <- data.frame(

location = paste0("TD_", 1:num_features),

MAE = mae_per_feature3

)

head(data_mae_window3)

write_csv(data_rmse_window3, "D:/quick_rmse_LSTM-7.csv")

write_csv(data_mae_window3, "D:/quick_mae_LSTM-7.csv")

mae_matrix_window3 <- abs(denorm_pred3 - denorm_true3)

mae_per_eye_loc <- as.data.frame(mae_matrix_window3)

colnames(mae_per_eye_loc) <- paste0("TD_", 1:num_features)

rownames(mae_per_eye_loc) <- test_indices

write_csv(mae_per_eye_loc, "D:/quick_mae_per_eye_loc_lstm_7.csv")

Supplement Material- LSTM Model Code

S2. LSTM Model Implementation for Examination 9 Under the Fast Progression Rate Scenario

rm(list = ls())

gc()

library(dplyr)

library(caret)

library(readr)

library(tensorflow)

library(keras)

library(openxlsx)

tensorflow::set_random_seed(123)

data <- read_csv("D:/felash10/USB Drive/phd/payanname phd/thesis/final.simulation/TD/models in quick progression_td/data_quick_ecc.csv")

nvisit <- 11

neye <- 12000

num_features <- 52

start_col_TD <- 5

start_col_PD <- start_col_TD + num_features

data$eye <- rep(1:neye, each = nvisit)

normalize <- function(x) {

(x - min(x, na.rm = TRUE)) / (max(x, na.rm = TRUE) - min(x, na.rm = TRUE))

{

normalized_TD_columns <- paste0("TD_", 1:num_features, "_norm")

normalized_PD_columns <- paste0("PD_", 1:num_features, "_norm")

data[normalized_TD_columns] <- lapply(data[start_col_TD:(start_col_TD + num_features - 1)], normalize)

data[normalized_PD_columns] <- lapply(data[start_col_PD:(start_col_PD + num_features - 1)], normalize)

set.seed(123)

test_indices <- sample(1:neye, size = 0.15 * neye)

train_indices <- setdiff(1:neye, test_indices)

inputs_list <- list()

outputs_list <- list()

create_windows <- function(eye_data, is_test = FALSE) {

input_list <- list()

output_list <- list()

if (is_test) {

input <- as.matrix(eye_data[4:8, c(normalized_TD_columns, normalized_PD_columns)])

output <- as.matrix(eye_data[9, normalized_TD_columns])

input_list[[1]] <- input

output_list[[1]] <- output

}else {

for (i in 1:4) {

input <- as.matrix(eye_data[i:(i+4), c(normalized_TD_columns, normalized_PD_columns)])

output <- as.matrix(eye_data[i+5, normalized_TD_columns])

input_list[[i]] <- input

output_list[[i]] <- output

{

{

return(list(inputs = input_list, outputs = output_list))

{

for (eye_id in 1:neye) {

eye_data <- data[data$eye == eye_id, ]

windows <- create_windows(eye_data, is_test = (eye_id %in% test_indices))

inputs_list[[eye_id]] <- windows$inputs

outputs_list[[eye_id]] <- windows$outputs

{

train_inputs <- inputs_list[train_indices]

train_outputs <- outputs_list[train_indices]

test_inputs <- inputs_list[test_indices]

test_outputs <- outputs_list[test_indices]

x_train <- as.array(train_inputs, dim = c(length(train_indices), 4, 5, num_features * 2))

y_train <- as.array(train_outputs, dim = c(length(train_indices), 4, num_features))

x_test <- as.array(test_inputs, dim = c(length(test_indices), 1, 5, num_features * 2))

y_test <- as.array(test_outputs, dim = c(length(test_indices), 1, num_features))

x_train_tensor <- as_tensor(unlist(x_train),shape=c(10200,4,5,104))

y_train_tensor <- as_tensor(unlist(y_train),shape=c(10200,4,52))

x_test_tensor <- as_tensor(unlist(x_test),shape=c(1800,1,5,104))

y_test_tensor <- as_tensor(unlist(y_test),shape=c(1800,1,52))

build_model <- function() {

input <- layer_input(shape = c(NA, 5, 104))

model <- input %>%

time_distributed(layer_lstm(units = 64, return_sequences = FALSE)) %>%

layer_lstm(units = 100, return_sequences = TRUE) %>%

layer_dropout(rate = 0.1) %>%

time_distributed(layer_dense(units = 52, activation = "linear",

kernel_regularizer = regularizer_l2(0.0001)))

model_final <- keras_model(inputs = input, outputs = model)

model_final %>% compile(

loss = "mean_squared_error",

optimizer = optimizer_adam(learning_rate = 0.0001),

metrics = c("mean_squared_error", "mean_absolute_error")

(

return(model_final)

{

model <- build_model()

early_stopping <- callback_early_stopping(

monitor = "val_loss",

patience = 4,

min_delta = 0.001,

restore_best_weights = TRUE

(

history <- model %>% fit(

x_train_tensor, y_train_tensor,

validation_split = 0.1,

epochs = 100,

batch_size = 512,

verbose = 2,

callbacks = list(early_stopping)

(

y_test_pred <- model %>% predict(x_test_tensor)

valuation_train <- model %>% evaluate(x_train_tensor, y_train_tensor, verbose = 0)

evaluation_test <- model %>% evaluate(x_test_tensor, y_test_tensor, verbose = 0)

cat("Training MSE (TD):", valuation_train[[2]], "\n")

cat("Testing MSE (TD):", evaluation_test[[2]], "\n")

library(dplyr)

library(Metrics)

y_true_array <- as.array(y_test_tensor)

y_pred_array <- as.array(y_test_pred)

y_true_4th <- y_true_array[, 1, ] # dim: num_eyes x 52

y_pred_4th <- y_pred_array[, 1, ]

denorm_true4 <- matrix(NA, nrow = nrow(y_true_4th), ncol = num_features)

denorm_pred4 <- matrix(NA, nrow = nrow(y_pred_4th), ncol = num_features)

for (i in 1:num_features) {

col_name <- paste0("TD_", i)

min_val <- min(data[[col_name]], na.rm = TRUE)

max_val <- max(data[[col_name]], na.rm = TRUE)

denorm_true4[, i] <- y_true_4th[, i] * (max_val - min_val) + min_val

denorm_pred4[, i] <- y_pred_4th[, i] * (max_val - min_val) + min_val

{

rmse_per_eye4 <- sqrt(rowMeans((denorm_pred4 - denorm_true4)^2, na.rm = TRUE))

data_rmse_window4 <- data.frame(

eye_id = test_indices,

rmse = rmse_per_eye4

(

head(data_rmse_window4)

mae_per_feature4 <- colMeans(abs(denorm_pred4 - denorm_true4), na.rm = TRUE)

data_mae_window4 <- data.frame(

location = paste0("TD_", 1:num_features),

MAE = mae_per_feature4

)

head(data_mae_window4)

write_csv(data_rmse_window4, "D:/quick_rmse_LSTM-8.csv")

write_csv(data_mae_window4, "D:/quick_mae_LSTM-8.csv")

mae_matrix_window4 <- abs(denorm_pred4 - denorm_true4)

mae_per_eye_loc <- as.data.frame(mae_matrix_window4)

colnames(mae_per_eye_loc) <- paste0("TD_", 1:num_features)

rownames(mae_per_eye_loc) <- test_indices

write_csv(mae_per_eye_loc, "D:/quick_mae_per_eye_loc_lstm_8.csv")

Supplement Material- LSTM Model Code

S2. LSTM Model Implementation for Examination 10 Under the Fast Progression Rate Scenario

rm(list = ls())

gc()

library(dplyr)

library(caret)

library(readr)

library(tensorflow)

library(keras)

library(openxlsx)

tensorflow::set_random_seed(123)

data <- read_csv("D:/felash10/USB Drive/phd/payanname phd/thesis/final.simulation/TD/models in quick progression_td/data_quick_ecc.csv")

nvisit <- 11

neye <- 12000

num_features <- 52

start_col_TD <- 5

start_col_PD <- start_col_TD + num_features

data$eye <- rep(1:neye, each = nvisit)

normalize <- function(x) {

(x - min(x, na.rm = TRUE)) / (max(x, na.rm = TRUE) - min(x, na.rm = TRUE))

}

normalized_TD_columns <- paste0("TD_", 1:num_features, "_norm")

normalized_PD_columns <- paste0("PD_", 1:num_features, "_norm")

data[normalized_TD_columns] <- lapply(data[start_col_TD:(start_col_TD + num_features - 1)], normalize)

data[normalized_PD_columns] <- lapply(data[start_col_PD:(start_col_PD + num_features - 1)], normalize)

set.seed(123)

test_indices <- sample(1:neye, size = 0.15 * neye)

train_indices <- setdiff(1:neye, test_indices)

inputs_list <- list()

outputs_list <- list()

create_windows <- function(eye_data, is_test = FALSE) {

input_list <- list()

output_list <- list()

if (is_test) {

input <- as.matrix(eye_data[5:9, c(normalized_TD_columns, normalized_PD_columns)])

output <- as.matrix(eye_data[10, normalized_TD_columns])

input_list[[1]] <- input

output_list[[1]] <- output

}else {

for (i in 1:5) {

input <- as.matrix(eye_data[i:(i+4), c(normalized_TD_columns, normalized_PD_columns)])

output <- as.matrix(eye_data[i+5, normalized_TD_columns])

input_list[[i]] <- input

output_list[[i]] <- output

}

}

return(list(inputs = input_list, outputs = output_list))

}

for (eye_id in 1:neye) {

eye_data <- data[data$eye == eye_id, ]

windows <- create_windows(eye_data, is_test = (eye_id %in% test_indices))

inputs_list[[eye_id]] <- windows$inputs

outputs_list[[eye_id]] <- windows$outputs

}

train_inputs <- inputs_list[train_indices]

train_outputs <- outputs_list[train_indices]

test_inputs <- inputs_list[test_indices]

test_outputs <- outputs_list[test_indices]

x_train <- as.array(train_inputs, dim = c(length(train_indices), 5, 5, num_features * 2))

y_train <- as.array(train_outputs, dim = c(length(train_indices), 5, num_features))

x_test <- as.array(test_inputs, dim = c(length(test_indices), 1, 5, num_features * 2))

y_test <- as.array(test_outputs, dim = c(length(test_indices), 1, num_features))

x_train_tensor <- as_tensor(unlist(x_train),shape=c(10200,5,5,104))

y_train_tensor <- as_tensor(unlist(y_train),shape=c(10200,5,52))

x_test_tensor <- as_tensor(unlist(x_test),shape=c(1800,1,5,104))

y_test_tensor <- as_tensor(unlist(y_test),shape=c(1800,1,52))

build_model <- function() {

input <- layer_input(shape = c(NA, 5, 104))

model <- input %>%

time_distributed(layer_lstm(units = 64, return_sequences = FALSE)) %>%

layer_lstm(units = 130, return_sequences = TRUE) %>%

layer_dropout(rate = 0.1) %>%

time_distributed(layer_dense(units = 52, activation = "linear",

kernel_regularizer = regularizer_l2(0.0001)))

model_final <- keras_model(inputs = input, outputs = model)

model_final %>% compile(

loss = "mean_squared_error",

optimizer = optimizer_adam(learning_rate = 0.0001),

metrics = c("mean_squared_error", "mean_absolute_error")

)

return(model_final)

}

model <- build_model()

early_stopping <- callback_early_stopping(

monitor = "val_loss",

patience = 4,

min_delta = 0.001,

restore_best_weights = TRUE

(

history <- model %>% fit(

x_train_tensor, y_train_tensor,

validation_split = 0.1,

epochs = 100,

batch_size = 512,

verbose = 2,

callbacks = list(early_stopping)

(

y_test_pred <- model %>% predict(x_test_tensor)

valuation_train <- model %>% evaluate(x_train_tensor, y_train_tensor, verbose = 0)

evaluation_test <- model %>% evaluate(x_test_tensor, y_test_tensor, verbose = 0)

cat("Training MSE (TD):", valuation_train[[2]], "\n")

cat("Testing MSE (TD):", evaluation_test[[2]], "\n")

library(dplyr)

library(Metrics)

y_true_array <- as.array(y_test_tensor)

y_pred_array <- as.array(y_test_pred)

y_true_5th <- y_true_array[, 1, ] # dim: num_eyes x 52

y_pred_5th <- y_pred_array[, 1, ]

denorm_true5 <- matrix(NA, nrow = nrow(y_true_5th), ncol = num_features)

denorm_pred5 <- matrix(NA, nrow = nrow(y_pred_5th), ncol = num_features)

for (i in 1:num_features) {

col_name <- paste0("TD_", i)

min_val <- min(data[[col_name]], na.rm = TRUE)

max_val <- max(data[[col_name]], na.rm = TRUE)

denorm_true5[, i] <- y_true_5th[, i] * (max_val - min_val) + min_val

denorm_pred5[, i] <- y_pred_5th[, i] * (max_val - min_val) + min_val

{

rmse_per_eye5 <- sqrt(rowMeans((denorm_pred5 - denorm_true5)^2, na.rm = TRUE))

data_rmse_window5 <- data.frame(

eye_id = test_indices,

rmse = rmse_per_eye5

(

head(data_rmse_window5)

mae_per_feature5 <- colMeans(abs(denorm_pred5 - denorm_true5), na.rm = TRUE)

data_mae_window5 <- data.frame(

location = paste0("TD_", 1:num_features),

MAE = mae_per_feature5

(

head(data_mae_window5)

write_csv(data_rmse_window5, "D:/quick_rmse_LSTM-9.csv")

write_csv(data_mae_window5, "D:/quick_mae_LSTM-9.csv")

mae_matrix_window5 <- abs(denorm_pred5 - denorm_true5)

mae_per_eye_loc <- as.data.frame(mae_matrix_window5)

colnames(mae_per_eye_loc) <- paste0("TD_", 1:num_features)

rownames(mae_per_eye_loc) <- test_indices

write_csv(mae_per_eye_loc, "D:/quick_mae_per_eye_loc_lstm_9.csv")

Supplement Material- LSTM Model Code

S2. LSTM Model Implementation for Examination 11 Under the Fast Progression Rate Scenario

rm(list = ls())

gc()

library(dplyr)

library(caret)

library(readr)

library(tensorflow)

library(keras)

library(openxlsx)

tensorflow::set_random_seed(123)

data <- read_csv("D:/felash10/USB Drive/phd/payanname phd/thesis/final.simulation/TD/models in quick progression_td/data_quick_ecc.csv")

nvisit <- 11

neye <- 12000

num_features <- 52

start_col_TD <- 5

start_col_PD <- start_col_TD + num_features

data$eye <- rep(1:neye, each = nvisit)

normalize <- function(x) {

(x - min(x, na.rm = TRUE)) / (max(x, na.rm = TRUE) - min(x, na.rm = TRUE))

}

normalized_TD_columns <- paste0("TD_", 1:num_features, "_norm")

normalized_PD_columns <- paste0("PD_", 1:num_features, "_norm")

data[normalized_TD_columns] <- lapply(data[start_col_TD:(start_col_TD + num_features - 1)], normalize)

data[normalized_PD_columns] <- lapply(data[start_col_PD:(start_col_PD + num_features - 1)], normalize)

set.seed(123)

test_indices <- sample(1:neye, size = 0.15 * neye)

train_indices <- setdiff(1:neye, test_indices)

inputs_list <- list()

outputs_list <- list()

create_windows <- function(eye_data, is_test = FALSE) {

input_list <- list()

output_list <- list()

if (is_test) {

input <- as.matrix(eye_data[6:10, c(normalized_TD_columns, normalized_PD_columns)])

output <- as.matrix(eye_data[11, normalized_TD_columns])

input_list[[1]] <- input

output_list[[1]] <- output

else {{

for (i in 1:6) {

input <- as.matrix(eye_data[i:(i+4), c(normalized_TD_columns, normalized_PD_columns)])

output <- as.matrix(eye_data[i+5, normalized_TD_columns])

input_list[[i]] <- input

output_list[[i]] <- output

}

}

return(list(inputs = input_list, outputs = output_list))

}

for (eye_id in 1:neye) {

eye_data <- data[data$eye == eye_id, ]

windows <- create_windows(eye_data, is_test = (eye_id %in% test_indices))

inputs_list[[eye_id]] <- windows$inputs

outputs_list[[eye_id]] <- windows$outputs

}

train_inputs <- inputs_list[train_indices]

train_outputs <- outputs_list[train_indices]

test_inputs <- inputs_list[test_indices]

test_outputs <- outputs_list[test_indices]

x_train <- as.array(train_inputs, dim = c(length(train_indices), 6, 5, num_features * 2))

y_train <- as.array(train_outputs, dim = c(length(train_indices), 6, num_features))

x_test <- as.array(test_inputs, dim = c(length(test_indices), 1, 5, num_features * 2))

y_test <- as.array(test_outputs, dim = c(length(test_indices), 1, num_features))

x_train_tensor <- as_tensor(unlist(x_train),shape=c(10200,6,5,104))

y_train_tensor <- as_tensor(unlist(y_train),shape=c(10200,6,52))

x_test_tensor <- as_tensor(unlist(x_test),shape=c(1800,1,5,104))

y_test_tensor <- as_tensor(unlist(y_test),shape=c(1800,1,52))

build_model <- function() {

input <- layer_input(shape = c(NA, 5, 104))

model <- input %>%

time_distributed(layer_lstm(units = 64, return_sequences = FALSE)) %>%

layer_lstm(units = 170, return_sequences = TRUE) %>%

layer_dropout(rate = 0.1) %>%

time_distributed(layer_dense(units = 52, activation = "linear",

kernel_regularizer = regularizer_l2(0.0001)))

model_final <- keras_model(inputs = input, outputs = model)

model_final %>% compile(

loss = "mean_squared_error",

optimizer = optimizer_adam(learning_rate = 0.0001),

metrics = c("mean_squared_error", "mean_absolute_error")

)

return(model_final)

}

model <- build_model()

early_stopping <- callback_early_stopping(

monitor = "val_loss",

patience = 4,

min_delta = 0.001,

restore_best_weights = TRUE

)

history <- model %>% fit(

x_train_tensor, y_train_tensor,

validation_split = 0.1,

epochs = 100,

batch_size = 512,

verbose = 2,

callbacks = list(early_stopping)

)

y_test_pred <- model %>% predict(x_test_tensor)

valuation_train <- model %>% evaluate(x_train_tensor, y_train_tensor, verbose = 0)

evaluation_test <- model %>% evaluate(x_test_tensor, y_test_tensor, verbose = 0)

cat("Training MSE (TD):", valuation_train[[2]], "\n")

cat("Testing MSE (TD):", evaluation_test[[2]], "\n")

library(dplyr)

library(Metrics)

y_true_array <- as.array(y_test_tensor)

y_pred_array <- as.array(y_test_pred)

y_true_6th <- y_true_array[, 1, ] # dim: num_eyes x 52

y_pred_6th <- y_pred_array[, 1, ]

denorm_true6 <- matrix(NA, nrow = nrow(y_true_6th), ncol = num_features)

denorm_pred6 <- matrix(NA, nrow = nrow(y_pred_6th), ncol = num_features)

for (i in 1:num_features) {

col_name <- paste0("TD_", i)

min_val <- min(data[[col_name]], na.rm = TRUE)

max_val <- max(data[[col_name]], na.rm = TRUE)

denorm_true6[, i] <- y_true_6th[, i] * (max_val - min_val) + min_val

denorm_pred6[, i] <- y_pred_6th[, i] * (max_val - min_val) + min_val

}

rmse_per_eye6 <- sqrt(rowMeans((denorm_pred6 - denorm_true6)^2, na.rm = TRUE))

data_rmse_window6 <- data.frame(

eye_id = test_indices,

rmse = rmse_per_eye6

)

head(data_rmse_window6)

mae_per_feature6 <- colMeans(abs(denorm_pred6 - denorm_true6), na.rm = TRUE)

data_mae_window6 <- data.frame(

location = paste0("TD_", 1:num_features),

MAE = mae_per_feature6

)

head(data_mae_window6)

write_csv(data_rmse_window6, "D:/quick_rmse_LSTM-10.csv")

write_csv(data_mae_window6, "D:/quick_mae_LSTM-10.csv")

mae_matrix_window6 <- abs(denorm_pred6 - denorm_true6)

mae_per_eye_loc <- as.data.frame(mae_matrix_window6)

colnames(mae_per_eye_loc) <- paste0("TD_", 1:num_features)

rownames(mae_per_eye_loc) <- test_indices

write_csv(mae_per_eye_loc, "D:/quick_mae_per_eye_loc_lstm_10.csv")
